# Expanding the synthesizable multisubstituted benzo[*b*]thiophenes *via* 6,7-thienobenzynes generated from *o*-silylaryl triflate-type precursors†

**DOI:** 10.1039/c8ra04035d

**Published:** 2018-06-13

**Authors:** Suguru Yoshida, Tomoko Kuribara, Takamoto Morita, Tsubasa Matsuzawa, Kazushi Morimoto, Takuya Kobayashi, Takamitsu Hosoya

**Affiliations:** Laboratory of Chemical Bioscience, Institute of Biomaterials and Bioengineering, Tokyo Medical and Dental University (TMDU) 2-3-10 Kanda-Surugadai, Chiyoda-ku Tokyo 101-0062 Japan s-yoshida@tmd.ac.jp thosoya.cb@tmd.ac.jp; Department of Medical Chemistry and Cell Biology, Graduate School of Medicine, Kyoto University Konoe-cho, Yoshida, Sakyo-ku Kyoto 606-8501 Japan

## Abstract

Various 2,3-disubstituted 6,7-thienobenzynes have been efficiently generated from the corresponding *o*-silylaryl triflate-type precursors by activation with fluoride ions. The method has expanded the scope of synthesizable multisubstituted benzothiophenes, including those with various heteroatom substituents, and can be applied to the synthesis of EP4 antagonist analogs.

## Abstract 2

Various multisubstituted benzothiophenes, including those with various heteroatom substituents and EP4 antagonist analogs, have been prepared easily *via* 6,7-thienobenzynes generated from *o*-silylaryl triflate-type precursors.

## Graphical abstract

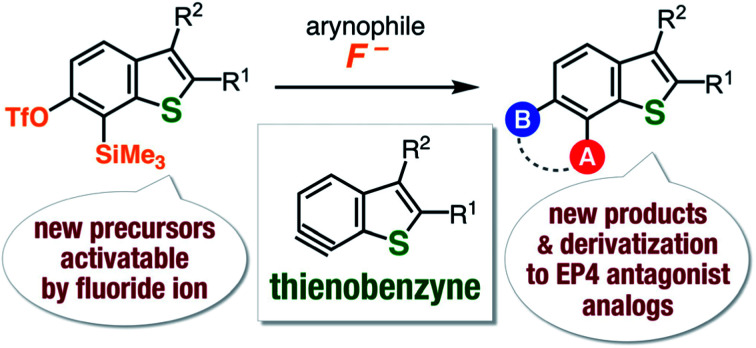

## Introduction

**Fig. 1:**
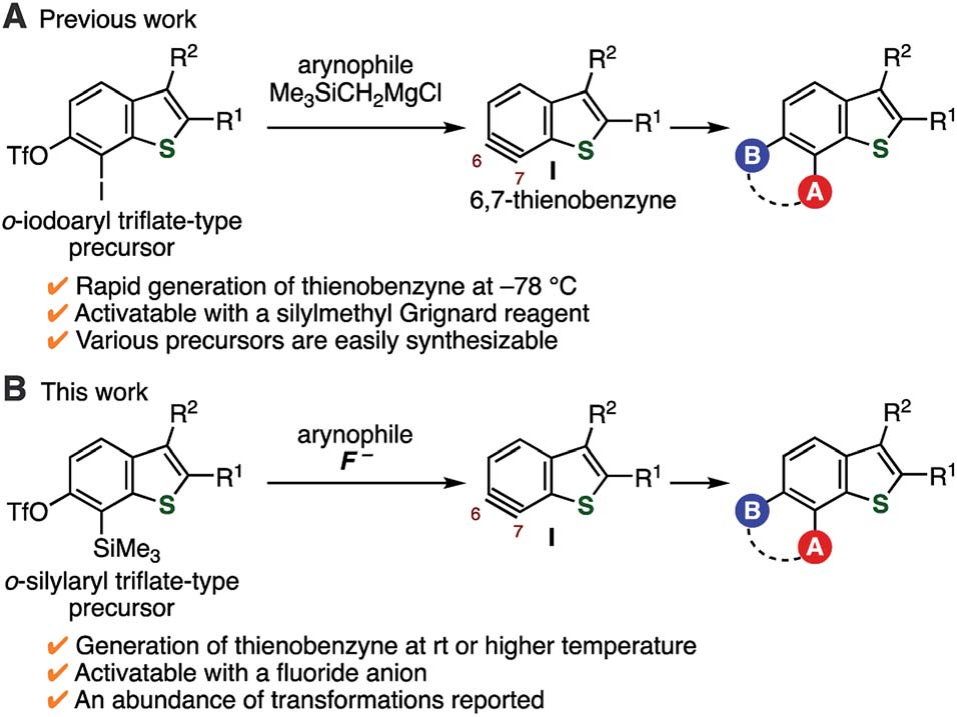
Transformations *via* thienobenzyne intermediates I. (A) Our previous work using *o*-iodoaryl triflate-type precursors. (B) This work using *o*-silylaryl triflate-type precursors.

Benzo[*b*]thiophene is one of the structural units frequently found in molecules applied in various research fields, including medicinal chemistry and materials science.^1–3^ Although multisubstituted benzothiophenes are promising compounds as pharmaceutical and organic material candidates, their synthetic approaches are limited.^4^ To improve this situation, we previously reported a facile method to prepare various tetrasubstituted benzothiophenes *via* thienobenzyne intermediates such as I (Fig. 1A).^5^ Thienobenzynes I were efficiently generated from *o*-iodoaryl triflate-type precursors by treatment with a silylmethyl Grignard reagent at −78 °C, rendering a diverse range of tetrasubstituted benzothiophenes easily available.^6^ We considered that the use of *o*-silylaryl triflate-type thienobenzyne precursors would further expand the scope of the synthesizable benzothiophenes (Fig. 1B). This is because generation of arynes from this type of precursor has been generally achieved under mild conditions using a basic activator such as the fluoride ion.^7–9^ Indeed, a wide range of aromatic compounds have become easily available *via* the transformation of arynes generated from *o*-silylaryl triflate-type precursors. Herein, we report the synthesis of *o*-silylaryl triflate-type 6,7-thienobenzyne precursors, the generation of aryne species from these precursors, and the application of the method to the synthesis of various benzothiophenes including potent analogs of a prostaglandin E receptor subtype 4 (EP4) antagonist.

## Results and discussion

### Synthesis of thienobenzyne precursors

**Scheme 1:**
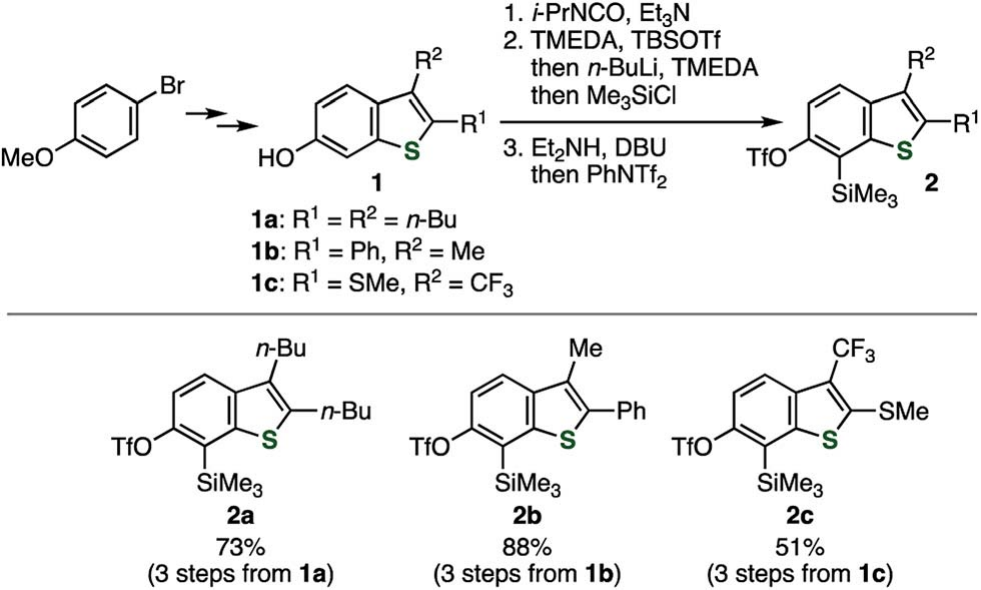
Synthesis of thienobenzyne precursors 2a–c. See the ESI† for details.

**Scheme 2:**
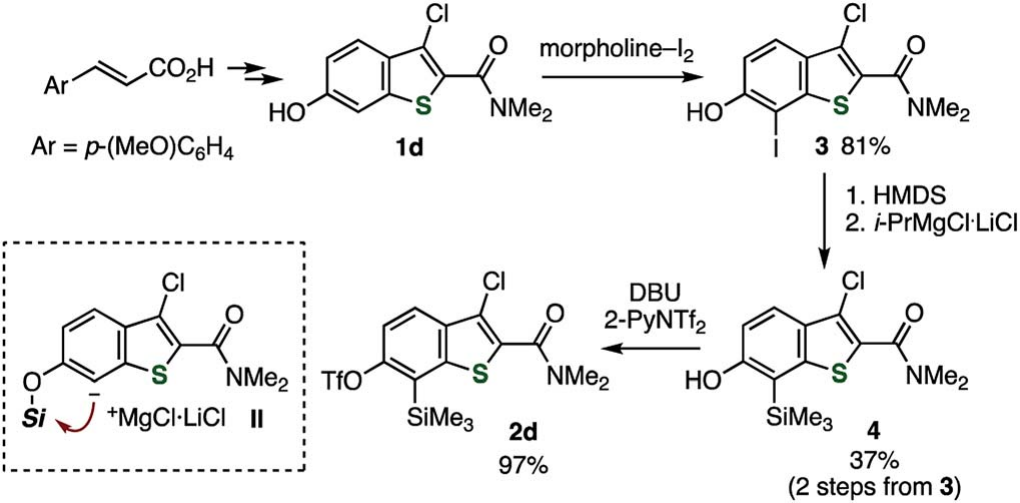
Synthesis of thienobenzyne precursor 2d. See the ESI† for details.

Similar to our previous synthesis of *o*-iodoaryl triflate-type 6,7-thienobenzyne precursors, *o*-silylaryl triflate-type precursors 2a–d were successfully prepared from the corresponding 2,3-disubstituted 6-hydroxybenzo[*b*]thiophenes 1a–d (Schemes 1 and 2).^5^ Benzothiophenes 2a–c were prepared from 6-hydroxybenzothiophenes 1a–c according to the facile synthetic method for *o*-silylaryl triflates from phenols as reported by Garg and coworkers; carbamate formation using isopropyl isocyanate, regioselective *C*-silylation *via ortho*-lithiation, removal of the directing group, and triflylation (Scheme 1).^10^ Although preparation of benzothiophene 2d, bearing a chloro and an amide group, from phenol 1d by the same method was unsuccessful at the step of *C*-silylation *via ortho*-lithiation, the *C*-silylated product was obtained by an alternative method (Scheme 2).^11^ Thus, regioselective iodination of phenol 1d with a morpholine–iodine complex, followed by *O*-silylation and treatment with the turbo Grignard reagent to promote the iodine–magnesium exchange reaction and subsequent retro-Brook rearrangement *via* the anionic intermediate II, afforded *o*-silylphenol 4, leaving the chloro and amide groups untouched. Finally, triflylation of 4 afforded the desired 2d.^12,13^ Performing the retro-Brook rearrangement and subsequent *O*-triflylation in one-pot procedure^12*a*^ afforded 2d in 13% yield.

### Optimization of the reaction conditions for generation of thienobenzynes

The efficient conditions for generating 6,7-thienobenzyne were screened for the reaction between precursor 2a and azide 5a in tetrahydrofuran (THF) at room temperature, which revealed that various fluoride sources or cesium carbonate with 18-crown-6 were effective as an activator (Table 1). For example, the activation of 2a with potassium fluoride in the presence of 18-crown-6 afforded the desired cycloadduct 6a with a small amount of regioisomer 6a′ (entry 1). The regioselectivity was slightly lower than that observed in the reaction using *o*-iodoaryl triflate-type 6,7-thienobenzyne precursor probably because the reaction triggered by silicate formation was conducted at a higher temperature. Tetra(*n*-butyl)ammonium difluoro(triphenyl)silicate and tetra(*n*-butyl)ammonium fluoride also served as good activators without any additives (entries 2 and 3). While using potassium fluoride alone was ineffective (entry 4), 2a was efficiently activated with cesium fluoride, resulting in the highest combined yield of cycloadducts 6a and 6a′ (entry 5). Considering that the generation of benzyne from *o*-(trimethylsilyl)phenyl triflate with cesium fluoride in THF was reported as inefficient,^9*a*^ this result suggests that thienobenzyne precursor 2a is more easily activatable than the simple *o*-silylphenyl triflate. Decreasing the amount of azide 5a to 2.0 equiv. slightly lowered the yield of 6a/6a′ (entry 6). In addition, 6,7-thienobenzyne was also generated efficiently under fluoride-free conditions using cesium carbonate and 18-crown-6 (entry 7).^9*a*^

**Table 1:** Optimization of the reaction conditions

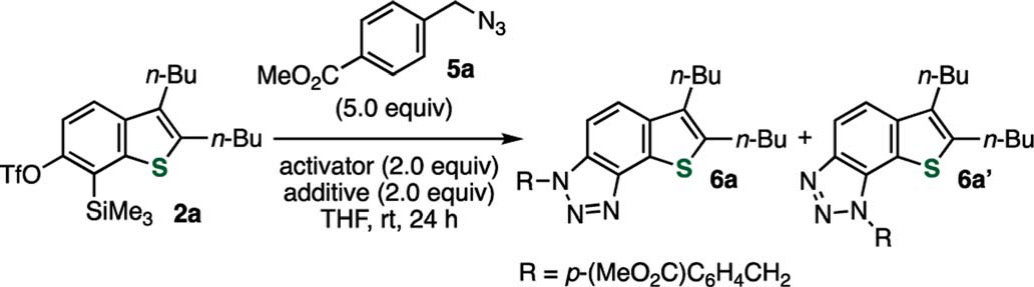			
Entry	Activator	Additive	Yield^a^ (%)
1	KF	18-Crown-6	78 (89 : 11)
2	*n*-Bu_4_N[Ph_3_SiF_2_]	—	73 (90 : 10)^b^
3^c^	*n*-Bu_4_NF	—	69 (91 : 9)
4	KF	—	0
5	CsF	—	84 (90 : 10)^b^
6^d^	CsF	—	74 (89 : 11)
7	Cs_2_CO_3_	18-Crown-6	75 (89 : 11)

a Yields were determined by ^1^H NMR analysis, unless otherwise noted.

b Isolated yield.

c Reaction was performed at 0 °C.

d Azide 5a (2.0 equiv.) was used.

### Synthesis of various multisubstituted benzothiophenes *via* thienobenzynes

**Fig. 2:**
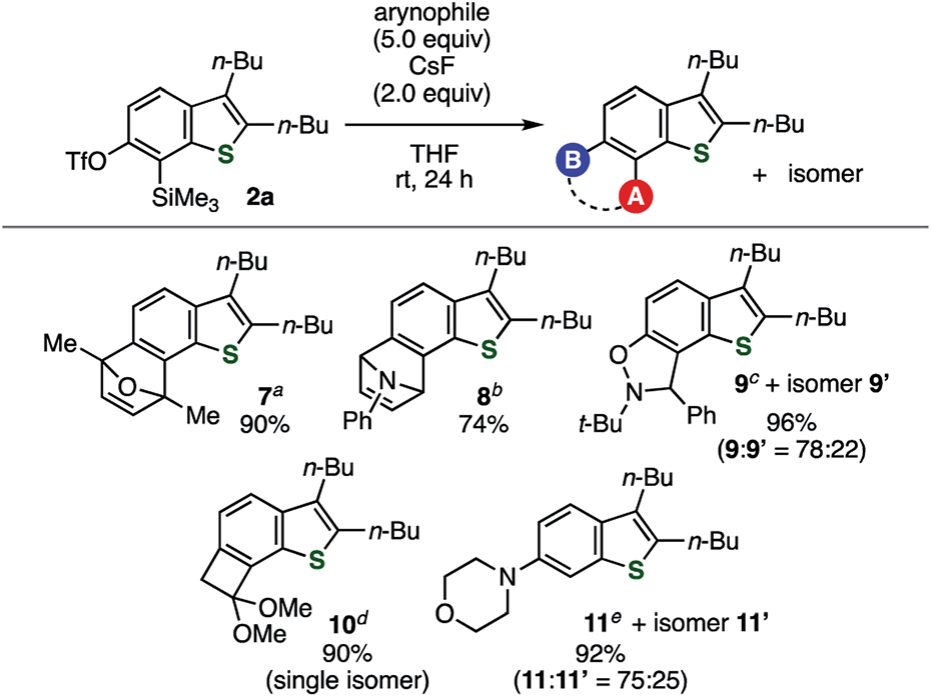
Reactions of thienobenzyne generated from 2a with various arynophiles. (a) Reaction with 2,5-dimethylfuran. (b) Reaction with *N*-phenylpyrrole. (c) Reaction with *N*-(*tert*-butyl)-α-phenylnitrone. (d) Reaction with 1,1-dimethoxyethylene. (e) Reaction with morpholine.

Under the optimal conditions, various arynophiles reacted efficiently with thienobenzyne generated from 2a to afford multisubstituted benzothiophenes in high yields (Fig. 2). These include cycloadducts 7, 8, 9/9′, and 10 obtained from the reactions with 2,5-dimethylfuran, *N*-phenylpyrrole, *N*-(*tert*-butyl)-α-phenylnitrone, and 1,1-dimethoxyethylene, respectively. The nucleophilic addition of morpholine to the 6,7-thienobenzyne also took place, affording 6-morpholinobenzothiophene 11 as the major product. The regioselectivity observed using unsymmetrical arynophiles and the nucleophile showed similar trends to their reactions with the same thienobenzyne species generated from the *o*-iodoaryl triflate-type precursor.^5^

**Fig. 3:**
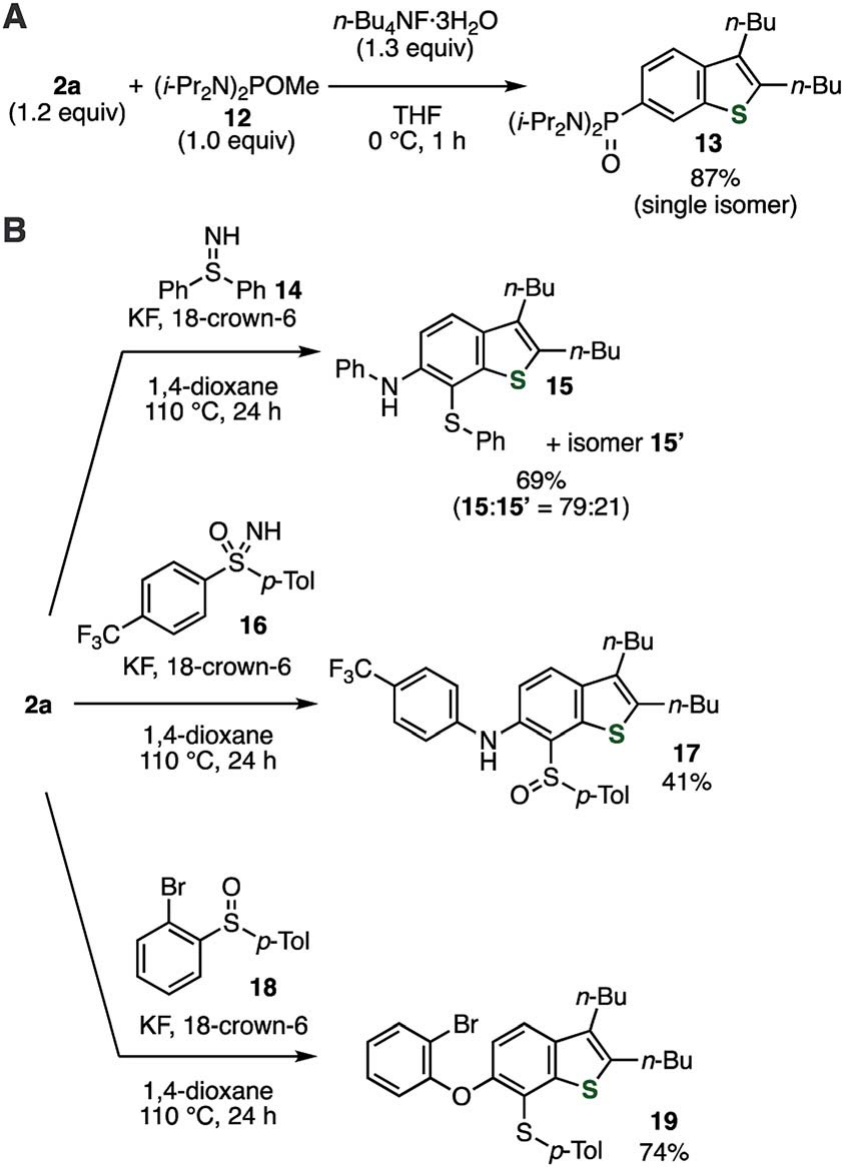
Transformations *via* thienobenzyne generated from 2a, involving C–P, C–S, C–N, and C–O bond formations. (A) Reaction with alkoxyphosphine 12. (B) Reactions with sulfilimine 14, sulfoximine 16, and sulfoxide 18. See the ESI† for details.

An abundance of utilizable transformations is a great advantage of using *o*-silylaryl triflates as aryne precursors over the other types. Indeed, the utility of *o*-silylaryl triflate-type 6,7-thienobenzyne precursor was demonstrated through several unique transformations that we recently developed (Fig. 3).^14^ For example, the Michaelis–Arbuzov-type reaction of the thienobenzyne generated from 2a with alkoxyphosphine 12 proceeded smoothly, affording a high yield of arylphosphonic diamide 13 as the sole product (Fig. 3A).^14*a*^ Furthermore, difunctionalizations of the thienobenzyne intermediate with sulfilimine 14,^14*b*^ sulfoximine 16,^14*c*^ and sulfoxide 18^14*d*^ resulted in the selective formation of thioaminated or oxythiolated benzothiophenes 15/15′, 17, and 19, respectively, which are difficult to prepare by conventional methods (Fig. 3B). The yields of thioaminated products 15/15′ and 17 were improved under modified conditions wherein the reactions were carried out at a higher temperature in 1,4-dioxane.

**Fig. 4:**
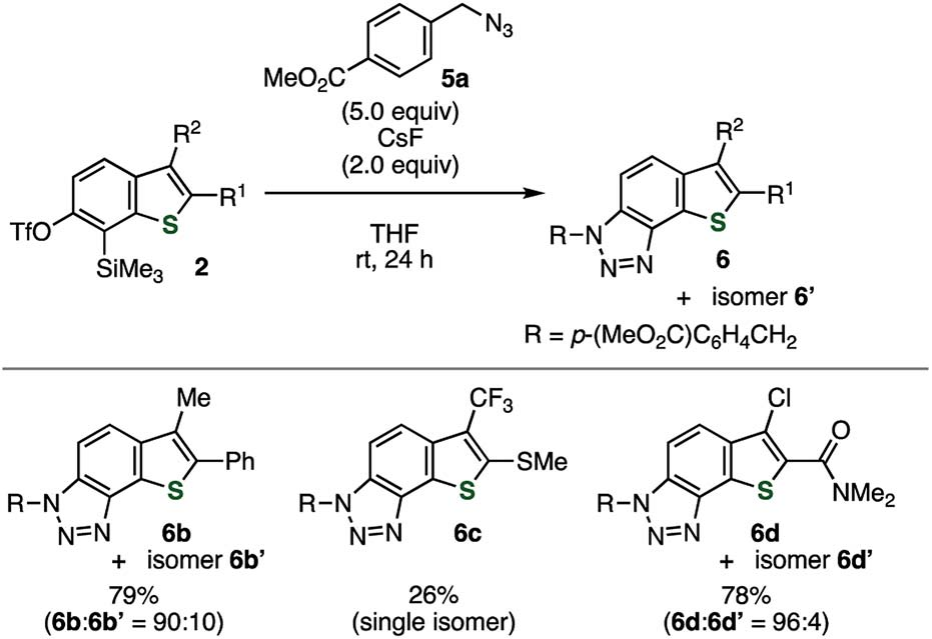
Cycloadditions of various thienobenzynes generated from precursors 2b–d with azide 5a.

Various 2,3-disubstituted 6,7-thienobenzynes were also generated from precursors 2b–d (Fig. 4). The reactions of these thienobenzynes with azide 5a afforded triazole-fused 3-methyl-2-phenyl-, 2-methylsulfanyl-3-trifluoromethyl-, and 3-chloro-2-(dimethylamino)carbonylbenzothiophene derivatives 6b/6b′, 6c, and 6d/6d′, respectively, in a regioselective manner. Cycloadduct 6c was obtained as a single isomer along with complex mixtures of side-products probably due to the effect of the electron-withdrawing trifluoromethyl group. A similar trend was observed in our previous study,^5^ wherein 6c was obtained without formation of the regioisomer using *o*-iodoaryl triflate-type aryne precursor activated with a silylmethyl Grignard reagent.

### Synthesis of the analogs of an EP4 antagonist

**Scheme 3:**
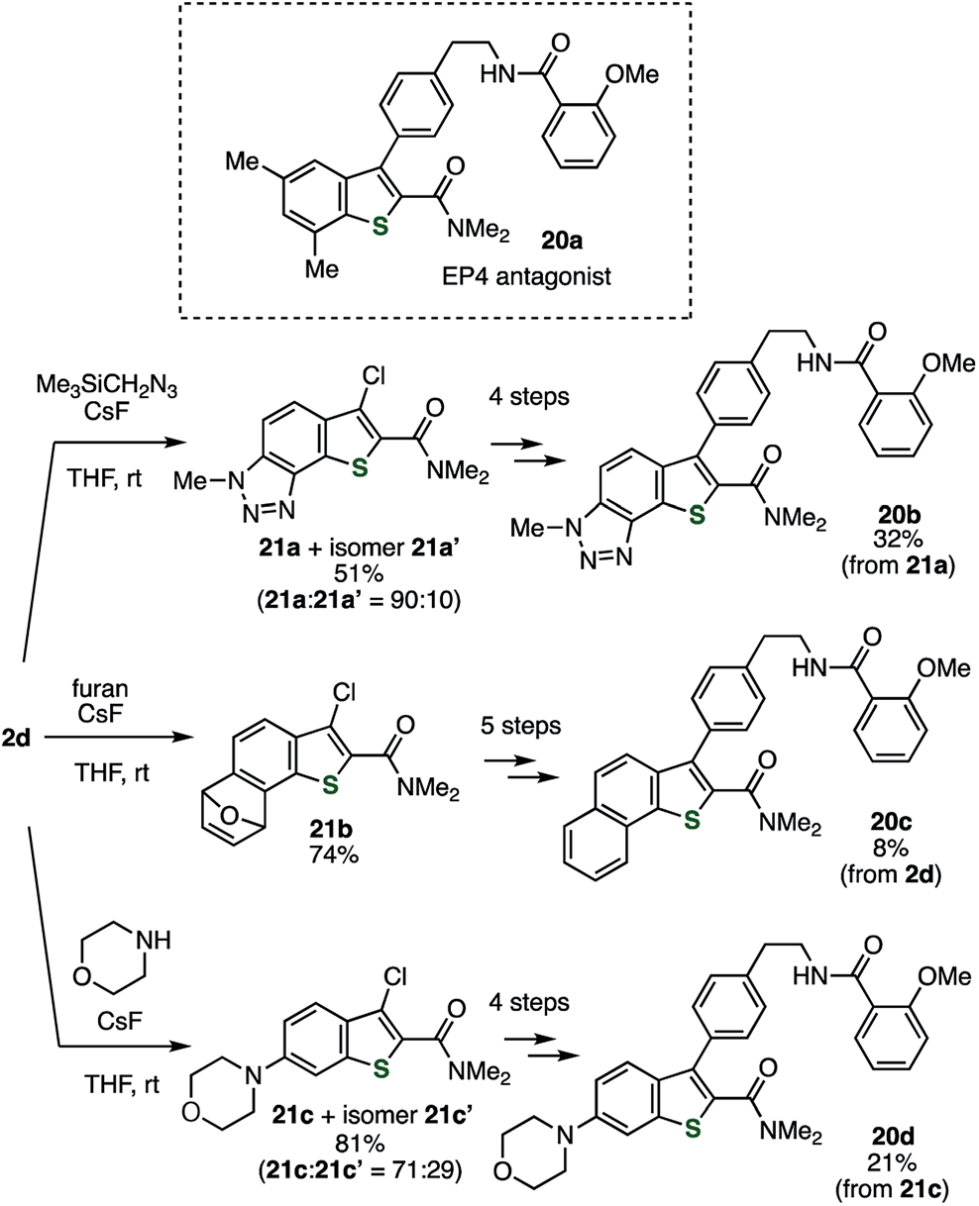
Synthesis of the analogs of EP4 antagonist 20a. See the ESI† for details.

The utility of this method was demonstrated in the facile diversification of the benzo-moiety of the EP4 antagonist 20a developed by Li and coworkers (Scheme 3).^15^ The analogs 20b–d with methyltriazole-fused, benzo-fused, or morpholino-substituted benzothiophene structure, respectively, were easily prepared *via* the reactions of the thienobenzyne intermediate generated from 2d with (trimethylsilyl)methyl azide, furan, and morpholine, affording adducts 21a–c as the major products. According to the modified method reported previously for the derivatization of 21a to 20b,^5^ EP4 antagonist analogs 20c and 20d were prepared by the Suzuki–Miyaura cross-coupling, the Mitsunobu-type C–N bond formation followed by treatment with hydrazine, and amidation. Evaluations of the EP4 receptor binding affinities showed that benzo-fused analog 20c (*K*_i_ = 0.18 μM) is a potent EP4 antagonist comparable to the original compound 20a (*K*_i_ = 0.25 μM), while methyltriazole-fused analog 20b (*K*_i_ = 0.47 μM) and morpholino-substituted analog 20d (*K*_i_ = 0.70 μM) are slightly weaker antagonists than 20a.^16^ This result suggests a possibility for developing more potent EP4 antagonists by further modification of the benzo-moiety of 20a.

## Conclusions

This study showed that 7-silyl-6-triflyloxybenzo[*b*]thiophenes served as useful precursors of 6,7-thienobenzynes, thus expanding the range of synthesizable multisubstituted benzothiophenes. The utility of the method was demonstrated for the synthesis of various heteroatom-substituted benzothiophenes and the facile structural diversification of an EP4 antagonist that resulted in identification of a potent analog.

## Conflicts of interest

There are no conflicts to declare.

## Supplementary Material

RA-008-C8RA04035D-s001
