# Supplementary material for: Expanding the synthesizable multisubstituted benzo[b]thiophenes via 6,7-thienobenzynes generated from o-silylaryl triflate-type precursors
Source: RSC Adv. 2018 Jun 13;8(39):21754–8. doi: 10.1039/c8ra04035d (PMC9081209; doi:10.1039/c8ra04035d)

Electronic Supplementary Material (ESI) for RSC Advances.
This journal is © The Royal Society of Chemistry 2018

Supporting Information

Expanding the synthesizable multisubstituted benzo[*b*]thiophenes via 6,7-thienobenzynes generated from *o*-silylaryl triflate-type precursors

Suguru Yoshida,*[1] Tomoko Kuribara,[1] Takamoto Morita,[1] Tsubasa Matsuzawa,[1] Kazushi Morimoto,[2] Takuya Kobayashi,[2] and Takamitsu Hosoya*[1]

[1]*Laboratory of Chemical Bioscience, Institute of Biomaterials and Bioengineering, Tokyo Medical and Dental University (TMDU), 2-3-10 Kanda-Surugadai, Chiyoda-ku, Tokyo 101-0062, Japan*
[2]*Department of Medical Chemistry and Cell Biology, Graduate School of Medicine, Kyoto University, Konoe-cho, Yoshida, Sakyo-ku, Kyoto 606-8501, Japan*

Contents

General Remarks **S1**
Experimental Procedures **S2**
Affinity determination of benzothiophene derivatives 20a–d to human EP4 receptor **S15**
References for Supporting Information **S17**
^{1}H and ^{13}C NMR Spectra of Compounds **S18**

General Remarks

All reactions were performed with dry glassware under atmosphere of argon, unless otherwise noted. Analytical thin-layer chromatography (TLC) was performed on precoated (0.25 mm) silica-gel plates (Merck Chemicals, Silica Gel 60 F_{254}, Cat. No. 1.05715). Column chromatography was conducted using silica-gel (Kanto Chemical Co., Inc., Silica Gel 60, spherical, particle size 40–50 μm, Cat. No. 37562-85), or Biotage® ZIP-sphere cartridge 45 g (Cat. No. 445-4500-SZ-20), 80 g (Cat. No. 445-8000-JZ-20), 120 g (Cat. No. 445-120G-UZ-20), or Biotage® SNAP Ultra HP-sphere cartridge 10 g (Cat. No. FSUL-0442-0010) or 25 g (Cat. No. FSUL-0442-0025) with medium pressure liquid chromatography (Yamazen, W-Prep 2XY A-type). Preparative thin-layer chromatography (PTLC) was performed on silica-gel (Wako Pure Chemical Industries Ltd., Wakogel® B-5F, Cat. No. 230-00043). Melting points (Mp) were measured on an OptiMelt MPA100 (Stanford Research Systems), and are uncorrected. ^{1}H NMR spectra were obtained with a Bruker AVANCE 500 spectrometer at 500 MHz. ^{13}C NMR spectra were obtained with a Bruker AVANCE 500 spectrometer at 126 MHz. ^{19}F NMR spectra (non-decoupling mode) were obtained with a Bruker AVANCE 400 spectrometer at 376 MHz. ^{31}P NMR spectra (non-decoupling mode) were obtained with a Bruker AVANCE 400 spectrometer at 162 MHz. All NMR measurements were carried out at 25 ºC. $CDCl_3$ (Kanto Chemical Co. Inc., Cat. No. 07663-23) or DMSO-d_6 (Kanto Chemical Co. Inc., Cat. No. 11560-43) was used as a solvent for obtaining NMR spectra. Chemical shifts (δ) are given in parts per million (ppm) downfield from $(CH_3)_4Si$ (δ 0.00 for ^{1}H NMR in $CDCl_3$) or the solvent peak (δ 77.0 for ^{13}C NMR in $CDCl_3$, and δ 2.49 for ^{1}H NMR and δ 39.5 for ^{13}C NMR in DMSO-d_6) as an internal reference, or α,α,α-trifluorotoluene (δ –63.0 ppm for ^{19}F NMR in $CDCl_3$) and 85%H_3PO_4 (δ 0.0 for ^{31}P NMR in $CDCl_3$) as an external standard with coupling constants (*J*) in hertz (Hz). The abbreviations s, d, t, q, sept, m, and br signify singlet, doublet, triplet, quartet, septet, multiplet, and broad, respectively. IR spectra were measured by diffuse reflectance method on a Shimadzu IRPrestige-21 spectrometer attached with DRS-8000A with the absorption band given in cm^{-1}. High-resolution mass spectra (HRMS) were measured on a Bruker micrOTOF mass spectrometer under positive elect rospray ionization (ESI^{+}) conditions. Elemental analyses were carried out at A Rabbit Science Japan Co., Ltd.

n-Butyllithium (1.6 M in *n*-hexane) was used after titrimetric determination of the concentration by the 1,10-phenanthroline method.[S1]

2,3-Dibutyl-6-hydroxybenzo[*b*]thiophene (**1a**),[S2] 6-hydroxy-3-methyl-2-phenybenzo[*b*]thiophene (**1b**),[S2] 6-hydroxy-2-methylthio-3-(trifluoromethyl)benzo[*b*]thiophene (**1c**),[S2] 3-chloro-2-dimethylaminocarbonyl-6-hydroxybenzo[*b*]thiophene (**1d**),[S2] methyl 4-(azidomethyl)benzoate (**5a**),[S3] *S*-(4-tolyl)-*S*-(4-(trifluoromethyl)-phenyl)sulfoximine (**16**),[S4] 2-bromophenyl 4-tolyl sulfoxide (**18**),[S5] and EP4 antagonist analog **20b**[S2] were prepared according to the reported methods. All other chemical reagents used were commercial grade and used as received.

Experimental Procedures

*Preparation of 2,3-dibutyl-6-triflyloxy-7-(trimethylsilyl)benzo[b]thiophene (**2a**)*

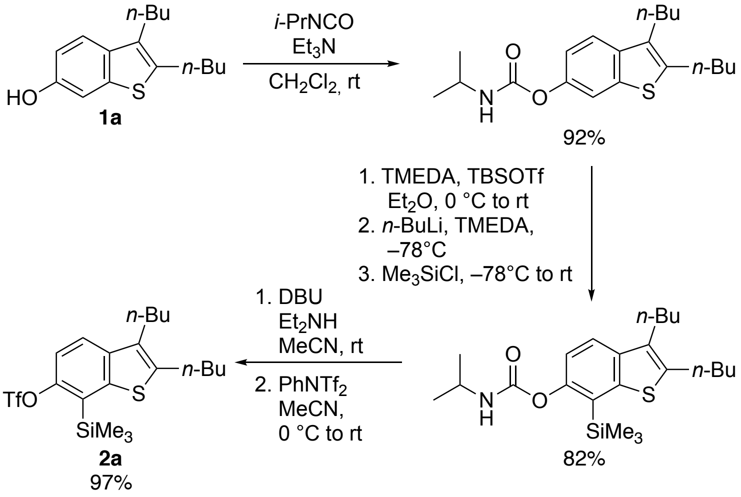

To a solution of 2,3-dibutyl-6-hydroxybenzo[*b*]thiophene (**1a**) (6.56 g, 25.0 mmol) dissolved in CH_2Cl_2 (50 mL) were slowly added isopropyl isocyanate (3.53 mL, 50.2 mmol, 2.01 equiv) and triethylamine (0.69 mL, 5.0 mmol, 20 mol %) at room temperature. After stirring for 3 h at the same temperature, the mixture was concentrated under reduced pressure. The residue was purified by flash column chromatography (Biotage® ZIP-sphere cartridge 120 g, *n*-hexane/CH_2Cl_2 = 80/20 to 50/50) to give 2,3-dibutyl-6-(isopropylcarbamyloxy)benzo[*b*]thiophene (7.95 g, 22.9 mmol, 91.6%) as a colorless solid.

Colorless solid; Mp 79–81 °C; TLC R_f 0.26 (*n*-hexane/CH_2Cl_2 = 1/1); ^{1}H NMR ($CDCl_3$, 500 MHz) δ 0.87–0.99 (m, 6H, 2 CH_3), 1.24 (d, 6H, J = 6.6 Hz, 2 CH_3), 1.35–1.46 (m, 4H, aliphatic), 1.51–1.60 (m, 2H, aliphatic), 1.62–1.71 (m, 2H, aliphatic), 2.74 (t, 2H, J = 7.8 Hz, aliphatic), 2.83 (t, 2H, J = 7.7 Hz, aliphatic), 3.91 (dsept, 1H, J = 6.6, 6.6 Hz, CH), 4.87 (d, 1H, J = 6.6 Hz, NH), 7.09 (dd, 1H, J = 8.6, 2.1 Hz, aromatic), 7.52 (d, 1H, J = 2.1 Hz, aromatic), 7.55 (d, 1H, J = 8.6 Hz, aromatic); ^{13}C NMR ($CDCl_3$, 126 MHz) δ 13.9 (1C), 14.0 (1C), 22.4 (1C), 22.8 (1C), 22.9 (2C), 26.2 (1C), 28.2 (1C), 32.2 (1C), 33.6 (1C), 43.4 (1C), 115.0 (1C), 118.3 (1C), 121.5 (1C), 131.1 (1C), 137.9 (1C), 138.8 (1C), 140.2 (1C), 147.3 (1C), 154.0 (1C); IR (KBr, cm^{-1}) 1029, 1057, 1175, 1206, 1238, 1466, 1526, 1717, 2859, 2930, 2957, 3323; Anal. calcd. for $C_{20}H_{29}NO_2S$: C, 69.12; H, 8.41; N, 4.03%; Found: C, 69.26, H, 8.39; N, 4.09%.

To a solution of 2,3-dibutyl-6-(isopropylaminocarbonyloxy)benzo[*b*]thiophene (691 mg, 2.00 mmol) dissolved in Et_2O (20 mL) were added *N,N,N',N'*-tetramethylethylenediamine (TMEDA) (0.33 mL, 2.2 mmol, 1.1 equiv) and *tert*-butyldimethylsilyl trifluoromethanesulfonate (TBSOTf) (0.51 mL, 2.2 mmol, 1.1 equiv) at 0 °C. After stirring for 5 min at the same temperature, the mixture was gradually warmed to room temperature, and to the mixture was added TMEDA (0.60 mL, 4.0 mmol, 2.0 equiv). After cooling to –78 °C, to the mixture was slowly added *n*-butyllithium (1.6 M in *n*-hexane, 2.5 mL, 4.0 mmol, 2.0 equiv), and stirred for 1 h at the same temperature. Then, to the mixture was added trimethylsilyl chloride (Me_3SiCl) (0.90 mL, 7.0 mmol, 3.5 equiv) at –78 °C, and the mixture was gradually warmed to room temperature. After stirring for 1.5 h at the same temperature, to the mixture was added saturated aqueous $NaHCO_3$ (20 mL). The mixture was extracted with CH_2Cl_2 (20 mL × 3), and the combined organic extract was dried (Na_2SO_4), and after filtration, the filtrate was concentrated under reduced pressure. The residue was purified by flash column chromatography (silica-gel 10 g, *n*-hexane/EtOAc = 5/1) to give 2,3-dibutyl-6-isopropylaminocarbonyloxy-7-(trimethylsilyl)benzo[*b*]thiophene (689 mg, 1.64 mmol, 82.0%) as a colorless solid.

Colorless solid; Mp 120–122 °C; TLC R_f 0.18 (*n*-hexane/CH_2Cl_2 = 2/1); ^{1}H NMR ($CDCl_3$, 500 MHz) δ 0.45 (s, 9H, 3 CH_3), 0.90–0.99 (m, 6H, 2 CH_3), 1.24 (d, 6H, J = 6.5 Hz, 2 CH_3), 1.35–1.47 (m, 4H, aliphatic), 1.51–1.59 (m, 2H, aliphatic), 1.64–1.72 (m, 2H, aliphatic), 2.73 (t, 2H, J = 7.8 Hz, aliphatic), 2.82 (t, 2H, J = 7.8 Hz, aliphatic), 3.93 (dsept, 1H, J = 6.5, 6.5 Hz, CH), 4.79 (br d, 1H, J = 6.5 Hz, NH), 7.05 (d, 1H, J = 8.6 Hz, aromatic), 7.58 (d, 1H, J = 8.6 Hz, aromatic); ^{13}C NMR ($CDCl_3$, 126 MHz) δ 0.8 (3C), 13.9 (1C), 14.0 (1C), 22.5 (1C), 22.8 (1C), 23.0 (2C), 26.1 (1C), 28.1 (1C), 32.2 (1C), 33.8 (1C), 43.4 (1C), 119.1 (1C), 122.9 (1C), 124.3 (1C), 130.7 (1C), 137.6 (1C), 139.7 (1C), 144.5 (1C), 152.3 (1C), 154.2 (1C); IR (KBr, cm^{-1}) 843, 1040, 1173, 1204, 1250, 1438, 1520, 1713, 2859, 2930, 2955, 3325; HRMS (ESI$^+$) *m/z* 442.2195 ([M+Na]$^+$, $C_{23}H_{37}NNaO_2SSi^+$ requires 442.2206).

To a solution of 2,3-dibutyl-6-isopropylaminocarbonyloxy-7-(trimethylsilyl)benzo[*b*]thiophene (552 mg, 1.31 mmol) dissolved in MeCN (13 mL) were added 1,8-diazabicyclo[5.4.0]undec-7-ene (DBU) (0.30 mL, 2.0 mmol, 1.5 equiv) and Et_2NH (0.16 mL, 1.6 mmol, 1.2 equiv) at room temperature. After stirring for 5 min at the same temperature, to the mixture was added a solution of *N*-phenylbis(trifluoromethanesulfonimide) ($PhNTf_2$) (702 mg, 1.96 mmol, 1.50 equiv) dissolved in MeCN (4.0 mL) at 0 °C. After warming the mixture to room temperature, it was stirred for 30 min, and to the mixture was added aqueous NH_4Cl (30 mL). The mixture was extracted with EtOAc (30 mL × 1), and the combined organic extract was washed with brine (10 mL × 3), dried (Na_2SO_4), and after filtration, the filtrate was concentrated under reduced pressure. The residue was purified by flash column chromatography (Biotage® ZIP-sphere cartridge 45 g, *n*-hexane) to give 2,3-dibutyl-6-triflyloxy-7-(trimethylsilyl)benzo[*b*]thiophene (**2a**) (594 mg, 1.27 mmol, 97.1%) as a colorless oil.

Colorless oil; TLC R_f 0.25 (*n*-hexane); ^{1}H NMR ($CDCl_3$, 500 MHz) δ 0.58 (s, 9H, 3 CH_3), 0.96–1.03 (m, 6H, 2 CH_3), 1.40–1.52 (m, 4H, aliphatic), 1.54–1.62 (m, 2H, aliphatic), 1.68–1.77 (m, 2H, aliphatic), 2.78 (t, 2H, *J* = 7.8 Hz, aliphatic), 2.89 (t, 2H, *J* = 7.8 Hz, aliphatic), 7.34 (d, 1H, *J* = 8.9 Hz, aromatic), 7.66 (d, 1H, *J* = 8.9 Hz, aromatic); ^{13}C NMR ($CDCl_3$, 126 MHz) δ 0.8 (3C), 13.9 (1C), 14.0 (1C), 22.6 (1C), 22.8 (1C), 26.1 (1C), 28.2 (1C), 32.2 (1C), 33.7 (1C), 116.3 (1C), 118.6 (q, 1C, $^1J_{C-F}$ = 321.2 Hz), 123.4 (1C), 125.6 (1C), 130.7 (1C), 139.2 (1C), 142.1 (1C), 145.2 (1C), 151.3 (1C); ^{19}F NMR ($CDCl_3$, 376 MHz) δ –73.5 (s); IR (KBr, cm^{-1}) 845, 910, 1142, 1211, 1250, 1418, 2860, 2932, 2957; Anal. calcd. for $C_{20}H_{29}F_3O_3S_2Si$: C, 51.48; H, 6.26%; Found: C, 51.40, H, 6.23%.

*Preparation of 3-methyl-2-pheny-6-triflyloxy-7-(trimethylsilyl)benzo[b]thiophene (**2b**)*

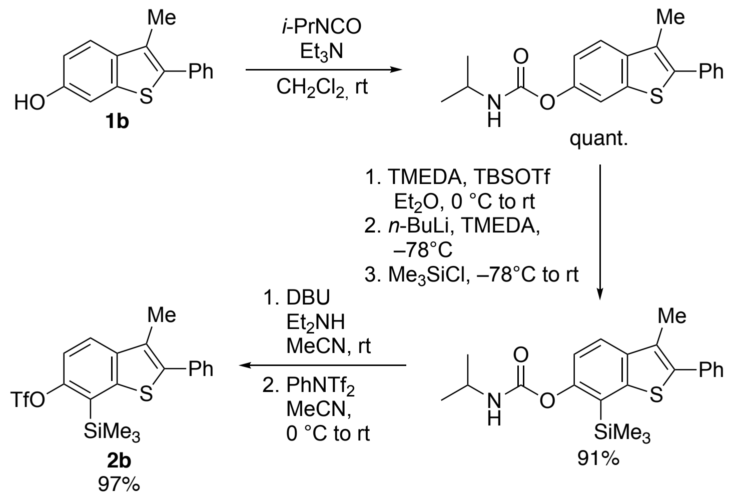

According to the synthetic procedure or **2a** from **1a**, 3-methyl-2-pheny-6-triflyloxy-7-(trimethylsilyl)-benzo[*b*]thiophene (**2b**) was prepared using 6-hydroxy-3-methyl-2-phenybenzo[*b*]thiophene (**1b**) instead of **1a**.

6-Isopropylaminocarbonyloxy-3-methyl-2-phenylbenzo[*b*]thiophene
Colorless solid; Mp 157–158 °C; TLC R_f 0.27 (*n*-hexane/CH_2Cl_2 = 2/3); ^{1}H NMR ($CDCl_3$, 500 MHz) δ 1.24 (d, 6H, *J* = 6.7 Hz, 2 CH_3), 2.44 (s, 3H, CH_3), 3.92 (dsept, 1H, *J* = 6.7, 6.7 Hz, CH), 4.92 (br d, 1H, *J* = 6.7 Hz, NH), 7.18 (dd, 1H, *J* = 8.6, 1.8 Hz, aromatic), 7.34–7.39 (AA'BB'C, 1H, aromatic), 7.42–7.48 (AA'BB'C, 2H, aromatic), 7.50–7.55 (AA'BB'C, 2H, aromatic), 7.61 (d, 1H, *J* = 1.8 Hz, aromatic), 7.66 (d, 1H, *J* = 8.6 Hz, aromatic); ^{13}C NMR ($CDCl_3$, 126 MHz) δ 12.7 (1C), 22.9 (2C), 43.5 (1C), 114.9 (1C), 118.9 (1C), 122.4 (1C), 127.0 (1C), 127.8 (1C), 128.5 (2C), 129.6 (2C), 134.6 (1C), 137.9 (1C), 138.7 (1C), 139.2 (1C), 148.2 (1C), 153.8 (1C); IR (KBr, cm^{-1}) 694, 752, 1059, 1173, 1204, 1242, 1454, 1468, 1531, 1697, 2974, 3057, 3331; HRMS (ESI$^+$) *m*/*z* 348.1028 ($[M+Na]^+$, $C_{19}H_{19}NNaO_2S^+$ requires 348.1029).

6-Isopropylaminocarbonyloxy-3-methyl-2-phenyl-7-(trimethylsilyl)benzo[*b*]thiophene
Colorless solid; Mp 158–159 °C; TLC R_f 0.18 (*n*-hexane/EtOAc = 10/1); ^{1}H NMR ($CDCl_3$, 500 MHz) δ 0.47 (s, 9H, 3 CH_3), 1.26 (d, 6H, *J* = 6.6 Hz, 2 CH_3), 2.43 (s, 3H, CH_3), 3.95 (dsept, 1H, *J* = 6.6, 6.6 Hz, CH), 4.81 (br d, 1H, *J* = 6.6 Hz, NH), 7.15 (d, 1H, *J* = 8.5 Hz, aromatic), 7.34–7.39 (AA'BB'C, 1H, aromatic), 7.42–7.48 (AA'BB'C, 2H, aromatic), 7.51–7.56 (AA'BB'C, 2H, aromatic), 7.70 (d, 1H, *J* = 8.5 Hz, aromatic); ^{13}C NMR ($CDCl_3$, 126 MHz) δ 0.8 (3C), 12.6 (1C), 23.0 (2C), 43.5 (1C), 119.8 (1C), 123.8 (1C), 124.4 (1C), 126.6 (1C), 127.7 (1C), 128.5 (2C), 129.7 (2C), 134.7 (1C), 137.7 (1C), 138.5 (1C), 145.0 (1C), 153.1 (1C), 154.1 (1C); IR (KBr, cm^{-1}) 698, 737, 845, 912, 1042, 1173, 1206, 1250, 1350, 1439, 1502, 1713, 2972, 3331; HRMS (ESI$^+$) *m*/*z* 420.1430 ($[M+Na]^+$, $C_{22}H_{27}NNaO_2SSi^+$ requires 420.1424).

3-Methyl-2-pheny-6-triflyloxy-7-(trimethylsilyl)benzo[*b*]thiophene (**2b**)
Colorless solid; Mp 75–76 °C; TLC R_f 0.30 (*n*-hexane); ^{1}H NMR ($CDCl_3$, 500 MHz) δ 0.58 (s, 9H, 3 CH_3), 2.45 (s, 3H, CH_3), 7.38–7.43 (m, 2H, aromatic), 7.45–7.51 (AA'BB'C, 2H, aromatic), 7.52–7.56 (AA'BB'C, 2H, aromatic), 7.75 (d, 1H, J = 9.0 Hz, aromatic); ^{13}C NMR ($CDCl_3$, 126 MHz) δ 0.8 (3C), 12.5 (1C), 116.8 (1C), 118.6 (q, 1C, $^1J_{C-F}$ = 321.2 Hz), 124.4 (1C), 125.8 (1C), 126.5 (1C), 128.2 (1C), 128.7 (2C), 129.7 (2C), 134.0 (1C), 140.0 (1C), 140.1 (1C), 145.6 (1C), 151.8 (1C); ^{19}F NMR ($CDCl_3$, 376 MHz) δ –73.4 (s); IR (KBr, cm^{-1}) 696, 760, 843, 910, 1140, 1211, 1248, 1350, 1418, 2953; Anal. calcd. for $C_{19}H_{19}F_3O_3S_2Si$: C, 51.33; H, 4.31%; Found: C, 51.43; H, 4.31%.

*Preparation of 2-(methylthio)-6-triflyloxy-3-(trifluoromethyl)-7-(trimethylsilyl)benzo[b]thiophene (**2c**)*

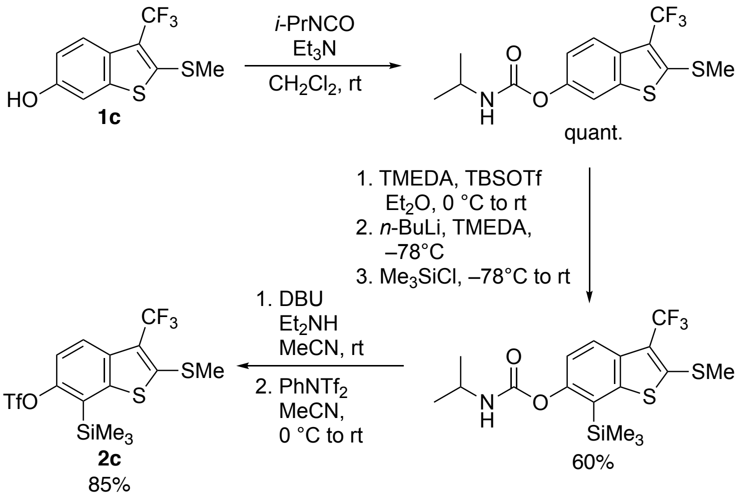

According to the synthetic procedure for **2a** from **1a**, 2-methylthio-6-triflyloxy-3-trifluoromethyl-7-(trimethylsilyl)benzo[*b*]thiophene (**2c**) was prepared using 6-hydroxy-2-methylthio-3-(trifluoromethyl)-benzo[*b*]thiophene (**1c**) instead of **1a**.

6-Isopropylaminocarbonyloxy-2-methylthio-3-(trifluoromethyl)benzo[*b*]thiophene
Colorless solid; Mp 111–112 °C; TLC R_f 0.39 (*n*-hexane/EtOAc = 3/1); ^{1}H NMR ($CDCl_3$, 500 MHz) δ 1.25 (d, 6H, J = 6.8 Hz, 2 CH_3), 2.65 (s, 3H, CH_3), 3.61 (dsept, 1H, J = 6.8, 6.8 Hz, CH), 4.91 (br d, 1H, J = 6.8 Hz, NH), 7.17 (dd, 1H, J = 9.0, 2.0 Hz, aromatic), 7.56 (d, 1H, J = 2.0 Hz, aromatic), 7.77 (d, 1H, J = 9.0 Hz, aromatic); ^{13}C NMR ($CDCl_3$, 126 MHz) δ 18.7 (1C), 22.9 (2C), 43.6 (1C), 114.3 (1C), 119.7 (q, 1C, $^2J_{C-F}$ = 34.3 Hz), 120.2 (1C), 122.3 (q, 1C, $^3J_{C-F}$ = 2.4 Hz), 123.1 (q, 1C, $^1J_{C-F}$ = 272.8 Hz), 134.3 (1C), 138.4 (1C), 145.1 (1C), 148.0 (1C), 153.4 (1C); ^{19}F NMR ($CDCl_3$, 376 MHz) δ –56.8 (s); IR (KBr, cm^{-1}) 920, 1038, 1065, 1111, 1159, 1225, 1364, 1464, 1505, 1530, 1713, 2359, 2974, 3331; HRMS (ESI$^+$) *m/z* 372.0313 ([M+Na]$^+$, $C_{14}H_{14}F_3NNaO_2S_2^+$ requires 372.0310).

6-Isopropylaminocarbonyloxy-2-methylthio-3-trifluoromethyl-7-(trimethylsilyl)benzo[*b*]thiophene
Colorless solid; Mp 121–122 °C; TLC R_f 0.24 (*n*-hexane/EtOAc = 10/1); ^{1}H NMR ($CDCl_3$, 500 MHz) δ 0.46 (s, 9H, 3 CH_3), 1.25 (d, 6H, J = 6.5 Hz, 2 CH_3), 2.66 (s, 3H, CH_3), 3.93 (dsept, 1H, J = 6.5, 6.5 Hz, CH), 4.82 (br d, 1H, J = 6.5 Hz, NH), 7.14 (d, 1H, J = 8.5 Hz, aromatic), 7.78–7.83 (m, 1H, aromatic); ^{13}C NMR ($CDCl_3$, 126 MHz) δ 0.7 (3C), 18.7 (q, 1C, J_{C-F} = 1.1 Hz), 23.0 (2C), 43.6 (1C), 119.8 (q, 1C, $^2J_{C-F}$ = 33.8 Hz), 121.1 (1C), 123.5 (q, 1C, $^3J_{C-F}$ = 2.4 Hz), 124.3 (q, 1C, $^1J_{C-F}$ = 272.9 Hz), 124.4 (1C), 134.5 (1C), 143.9 (q, 1C, $^3J_{C-F}$ = 2.1 Hz), 144.2 (1C), 152.9 (1C), 153.7 (1C); ^{19}F NMR ($CDCl_3$, 376 MHz) δ –56.6 (s); IR (KBr, cm^{-1}) 843, 941, 1040, 1123, 1146, 1173, 1207, 1234, 1344, 1371, 1441, 1504, 1713, 2972, 3327; Anal. calcd. for $C_{17}H_{22}F_3NO_2S_2Si$: C, 48.44; H, 5.26; N, 3.32%; Found: C, 48.49; H, 5.45; N, 3.28%.

2-Methylthio-6-triflyloxy-3-trifluoromethyl-7-(trimethylsilyl)benzo[*b*]thiophene (**2c**)
Colorless solid; Mp 69–70 °C; TLC R_f 0.26 (*n*-hexane); ^{1}H NMR ($CDCl_3$, 500 MHz) δ 0.56 (s, 9H, 3 CH_3), 2.70 (s, 3H, CH_3), 7.40 (d, 1H, J = 9.0 Hz, aromatic), 7.83–7.87 (m, 1H, aromatic); ^{13}C NMR ($CDCl_3$, 126 MHz) δ 0.8 (3C), 18.4 (q, 1C, J_{C-F} = 1.5 Hz), 118.2 (1C), 118.5 (q, 1C, $^1J_{C-F}$ = 321.3 Hz), 118.9 (q, 1C, $^2J_{C-F}$ = 3.6 Hz), 123.1 (q, 1C, $^1J_{C-F}$ = 272.6 Hz), 124.0 (q, 1C, $^3J_{C-F}$ = 2.7 Hz), 125.8 (1C), 136.2 (1C), 144.5 (1C), 147.1 (q, 1C, $^3J_{C-F}$ = 2.5 Hz), 151.4 (1C); ^{19}F NMR ($CDCl_3$, 376 MHz) δ –73.4 (s, 3F), –56.7 (s, 3F); IR (KBr, cm^{-1}) 613, 845, 908, 939, 1040, 1117, 1138, 1175, 1213, 1344, 1471, 1421, 1501, 2359, 2957; Anal. calcd. for $C_{14}H_{14}F_6O_3S_3Si$: C, 35.89; H, 3.01%; Found: C, 36.07; H 2.90%.

*Preparation of 3-chloro-2-dimethylaminocarbonyl-6-triflyloxy-7-(trimethylsilyl)benzo[b]thiophene (**2d**)*

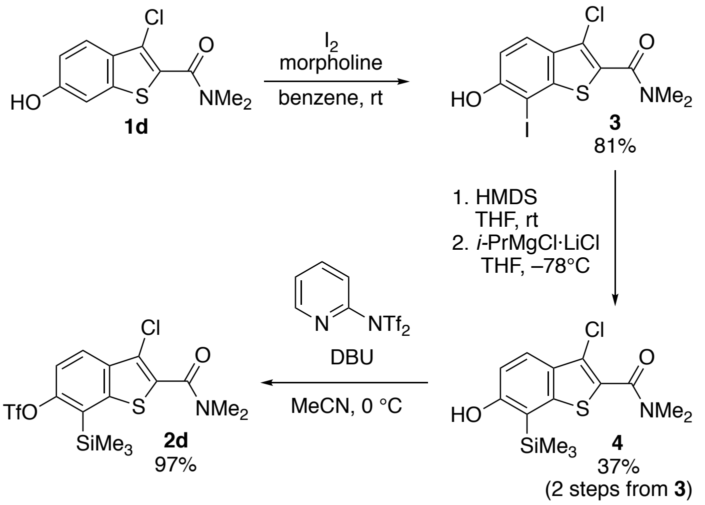

To a solution of iodine (5.08 g, 20.0 mmol, 1.0 equiv) dissolved in benzene (72 mL) was added morpholine (7.83 g, 60.0 mmol, 3.0 equiv) at room temperature. After stirring for 30 min at the same temperature, to the mixture was added 3-chloro-2-dimethylaminocarbonyl-6-hydroxybenzo[*b*]thiophene (**1d**) (5.11 g, 20.0 mmol) at room temperature. After stirring for 1 h at the same temperature, the precipitate was collected by filtration and washed on the funnel with acetone (20 mL × 3), aqueous 1 M HCl (20 mL × 3), and H_2O (20 mL × 3). The solid was dried under reduced pressure to give 3-chloro-2-dimethylaminocarbonyl-6-hydroxy-7-iodobenzo[*b*]thiophene (**3**) (6.18 g, 16.2 mmol, 81.0%) as a colorless solid.

Colorless solid; Mp 200 °C (decomposed); TLC R_f 0.43 (*n*-hexane/EtOAc = 1/1); ^{1}H NMR (DMSO-d_6, 500 MHz) δ 3.02 (br s, 6H, 2 CH_3), 7.11 (d, 1H, J = 8.5 Hz, aromatic), 7.69 (d, 1H, J = 8.5 Hz, aromatic), 10.94 (br s, 1H, OH); ^{13}C NMR (DMSO-d_6, 126 MHz) δ 34.8 (1C), 38.1 (1C), 76.8 (1C), 114.6 (1C), 118.7 (1C), 123.1 (1C), 127.4 (1C), 127.7 (1C), 144.8 (1C), 156.9 (1C), 161.5 (1C); IR (KBr, cm^{-1}) 748, 772, 806, 1283, 1379, 1531, 1603, 3011; Anal. calcd. for $C_{11}H_9ClINO_2S$: C, 34.62; H, 2.38; N, 3.67%; Found: C, 34.73; H 2.30; N, 3.62%.

To a solution of 3-chloro-2-dimethylaminocarbonyl-6-hydroxy-7-iodobenzo[*b*]thiophene (**3**) (3.81 g, 10.0 mmol) dissolved in THF (10 mL) was added bis(trimethylsilyl)amine (HMDS) (1.94 g, 12.0 mmol, 1.2 equiv) at room temperature. After stirring for 16 h at the same temperature, the mixture was concentrated under reduced pressure to give a crude product containing 3-chloro-2-dimethylaminocarbonyl-7-iodo-6-(trimethylsilyloxy)-benzo[*b*]thiophene (4.58 g, ca. 10 mmol) as a yellow oil. This crude product was used in the next step without further purification.

To a solution of the crude of 3-chloro-2-dimethylaminocarbonyl-7-iodo-6-(trimethylsilyloxy)benzo[*b*]thiophene (4.58 g, ca. 10 mmol) dissolved in THF (100 mL) was added isopropylmagnesium chloride lithium chloride complex (1.34 M in THF, 11.2 mL, 15.0 mmol, 1.5 equiv) at –78 °C. After stirring for 1 h at the same temperature, to the mixture was added aqueous saturated NH_4Cl (100 mL). The mixture was extracted with EtOAc (30 mL × 3) and the combined organic extract was washed with brine, dried (Na_2SO_4), and after filtration, the filtrate was concentrated under reduced pressure. The residue was purified by flash column chromatography (Biotage® ZIP-sphere cartridge 80 g, *n*-hexane/EtOAc = 100/0 to 60/40) to give 3-chloro-2-dimethylaminocarbonyl-6-hydroxy-7-(trimethylsilyl)benzo[*b*]thiophene (**4**) (1.20 g, 3.66 mmol, 36.6%) as a colorless solid.

Colorless solid; Mp 145–147 °C; TLC R_f 0.31 (*n*-hexane/EtOAc = 1/1); ^{1}H NMR ($CDCl_3$, 500 MHz) δ 0.49 (s, 9H, 3 CH_3), 3.13 (br s, 3H, CH_3), 3.19 (br s, 3H, CH_3), 5.67 (s, 1H, OH), 6.89 (d, 1H, J = 8.6 Hz, aromatic), 7.68 (d, 1H, J = 8.6 Hz, aromatic); ^{13}C NMR ($CDCl_3$, 126 MHz) δ 0.6 (3C), 35.4 (1C), 38.7 (1C), 115.2 (1C), 117.4 (1C), 118.7 (1C), 124.5 (1C), 127.0 (1C), 129.5 (1C), 144.9 (1C), 160.1 (1C), 163.7 (1C); IR (KBr, cm^{-1}) 772, 843, 1265, 1362, 1531, 1614, 2949, 3225; HRMS (ESI$^+$) m/z 350.0420 ($[M+Na]^+$, $C_{14}H_{18}{}^{35}ClNNaO_2SSi^+$ requires 350.0408).

To a solution of 3-chloro-2-dimethylaminocarbonyl-6-hydroxy-7-(trimethylsilyl)benzo[*b*]thiophene (**4**) (328 mg, 1.00 mmol) dissolved in MeCN (3 mL) were added DBU (183 mg, 1.20 mmol, 1.20 equiv) and *N*-(2-pyridyl)bis(trifluoromethanesulfonimide) (537 mg, 1.50 mmol, 1.50 equiv) at 0 °C. After stirring for 5 min at the same temperature, to the mixture was added water (3 mL). The mixture was extracted with EtOAc (5 mL × 3) and the combined organic extract was washed with brine, dried (Na_2SO_4), and after filtration, the filtrate was

concentrated under reduced pressure. The residue was purified by flash column chromatography (Biotage® SNAP Ultra HP-sphere cartridge 25 g, *n*-hexane/EtOAc = 100/0 to 67/33) to give 3-chloro-2-dimethylaminocarbonyl-6-triflyloxy-7-(trimethylsilyl)benzo[*b*]thiophene (**2d**) (446 mg, 0.97 mmol, 97.0%) as a colorless solid.

Colorless solid; Mp 92–93 °C; TLC R_f 0.33 (*n*-hexane/EtOAc = 3/1); ^{1}H NMR ($CDCl_3$, 500 MHz) δ 0.56 (s, 9H, 3 CH_3), 3.09 (br s, 3H, CH_3), 3.18 (br s, 3H, CH_3), 7.48 (d, 1H, J = 9.0 Hz, aromatic), 7.90 (d, 1H, J = 9.0 Hz, aromatic); ^{13}C NMR ($CDCl_3$, 126 MHz) δ 0.7 (3C), 35.4 (1C), 38.5 (1C), 118.2 (1C), 118.5 (q, 1C, $^1J_{C-F}$ = 321.4 Hz), 118.5 (1C), 125.1 (1C), 127.0 (1C), 132.3 (1C), 134.6 (1C), 144.2 (1C), 153.1 (1C), 162.4 (1C); ^{19}F NMR ($CDCl_3$, 376 MHz) δ –73.4 (s); IR (KBr, cm^{-1}) 845, 910, 980, 1144, 1211, 1250, 1402, 1422, 1643, 2953; Anal. calcd. for $C_{15}H_{17}ClF_3NO_4S_2Si$: C, 39.17; H, 3.73; N, 3.05%; Found: C, 39.19; H, 3.64; N, 3.05%.

A typical procedure for the reaction via 6,7-thienobenzyne

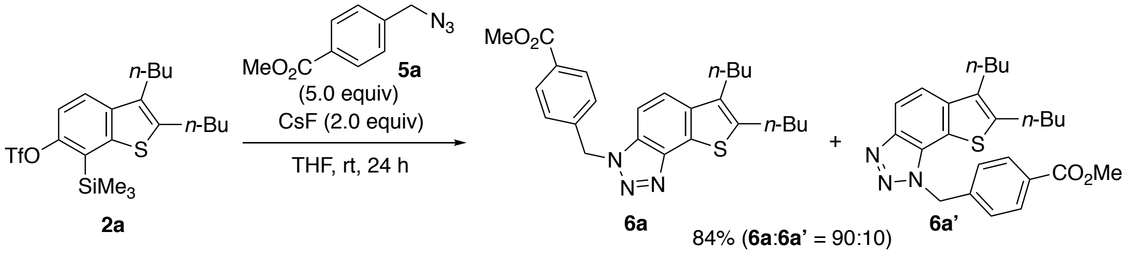

To a solution of 2,3-dibutyl-6-triflyloxy-7-(trimethylsilyl)benzo[*b*]thiophene (**2a**) (46.7 mg, 0.100 mmol) and methyl 4-(azidomethyl)benzoate (**5a**) (96.5 mg, 0.504 mmol, 5.04 equiv) dissolved in THF (1.0 mL) was added cesium fluoride (30.5 mg, 0.201 mmol, 2.01 equiv) at room temperature. After stirring for 24 h at the same temperature, the mixture was filtrated through a pad of Celite® washing with CH_2Cl_2 (0.5 mL × 5), and the filtrate was concentrated under reduced pressure. The residue was purified by preparative TLC (*n*-hexane/EtOAc = 4/1) to give a mixture of 6,7-dibutyl-3-(4-(methoxycarbonyl)benzyl)-3*H*-1,2,3-triazolo[3,4]benzo[1,2-*d*]thiophene (**6a**) and 6,7-dibutyl-1-(4-(methoxycarbonyl)benzyl)-1*H*-1,2,3-triazolo[3,4]benzo[1,2-*d*]thiophene (**6a'**) (36.8 mg, 84.4 μmol, 84.4%, **6a**:**6a'** = 90:10 as judged from ^{1}H NMR analysis) as a colorless solid. Cycloadducts **6a** and **6a'** were identical in spectra data with our previous report.[S2]

Similarly, benzothiophenes **6b**–**d** were prepared via the reaction of azide **5a** with 6,7-thienobenzynes generated from precursors **2b**–**d**, respectively. Benzothiophene derivatives **7**–**11** were also prepared via the reaction of thienobenzyne generated from **2a** with 2,5-dimethylfuran, *N*-phenylpyrrole, *N*-(*tert*-butyl)-α-phenylnitrone, 1,1-dimethoxyethylene, and morpholine, respectively. 3-(4-(Methoxycarbonyl)-benzyl)-6-methyl-7-phenyl-3*H*-1,2,3-triazolo[3,4]benzo[1,2-*d*]thiophene (**6b**), 3-(4-(methoxycarbonyl)benzyl)-7-methylthio-6-trifluoromethyl-3*H*-1,2,3-triazolo[3,4]benzo[1,2-*d*]thiophene (**6c**), 6-chloro-7-dimethylamino-carbonyl-3-(4-(methoxycarbonyl)benzyl)-3*H*-1,2,3-triazolo[3,4]benzo[1,2-*d*]thiophene (**6d**), 2,3-dibutyl-6,9-dimethyl-6,9-dihydro-6,9-epoxynaphtho[1,2-*b*]thiophene (**7**), 2,3-dibutyl-10-phenyl-6,9-dihydro-6,9-iminonaph-tho[1,2-*b*]thiophene (**8**), 7-(*tert*-butyl)-2,3-dibutyl-8-phenyl-7,8-dihydroisoxazolo[4',5':6,5]benzo[2,1-*d*]thio-phene (**9**), 2-(*tert*-butyl)-6,7-dibutyl-3-phenyl-2,3-dihydroisoxazolo[4',5':5,6]benzo[2,1-*d*]thiophene (**9'**), 2,3-dibutyl-7,7-dimethoxy-6,7-dihydrocyclobuta[3,4]benzo[1,2-*d*]thiophene (**10**), 2,3-dibutyl-6-morpholinobenzo-[*b*]thiophene (**11**) and 2,3-dibutyl-7-morpholinobenzo[*b*]thiophene (**11'**) were identical in spectra data with our previous report.[S2]

1-(4-(Methoxycarbonyl)benzyl)-6-methyl-7-phenyl-1*H*-1,2,3-triazolo[3,4]benzo[1,2-*d*]thiophene (**6b'**)

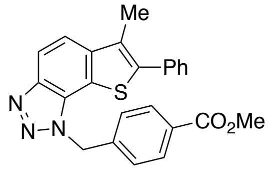

Colorless solid; Mp 207–20 °C; TLC R_f 0.40 (*n*-hexane/EtOAc = 3/1); ^{1}H NMR ($CDCl_3$, 500 MHz) δ 2.53 (s, 3H, CH_3), 3.87 (s, 3H, CH_3), 6.10 (s, 2H, aliphatic), 7.33–7.37 (AA'BB', 2H, aromatic), 7.39–7.44 (m, 1H, aromatic), 7.45–7.51 (m, 4H, aromatic), 7.71 (d, 1H, J = 8.8 Hz, aromatic), 7.96–8.00 (AA'BB', 2H, aromatic), 8.01 (d, 1H, J = 8.8 Hz, aromatic); ^{13}C NMR ($CDCl_3$, 126 MHz) δ 13.2 (1C), 52.2 (1C), 52.5 (1C), 116.5 (1C), 119.3 (1C), 119.6 (1C), 127.3 (2C), 128.3 (1C), 128.7 (2C), 128.9 (1C), 129.2 (1C), 129.8 (2C), 130.3 (2C+1C, two signals overlapped), 133.7 (1C), 139.2 (1C), 139.9 (1C), 141.0 (1C), 144.3 (1C), 166.5 (1C); IR (KBr, cm^{-1}) 739, 1109, 1281, 1435, 1721, 2920, 2951; HRMS (ESI^+) *m/z* 436.1101 ($[M+Na]^+$, $C_{24}H_{19}N_3NaO_2S^+$ requires 436.1090).

*Synthesis of arylphosphonic diamide **13** via the reaction of 6,7-thienobenzyne generated from **2a** with alkoxyphosphine **12***

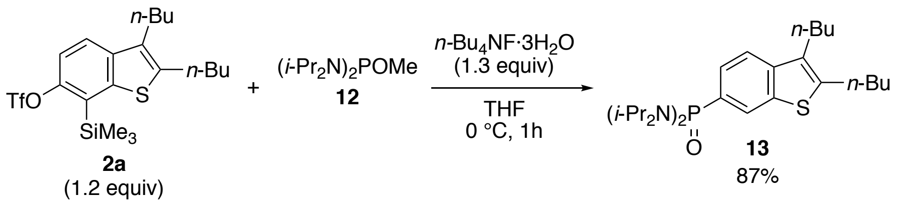

To a mixture of 2,3-dibutyl-6-triflyloxy-7-(trimethylsilyl)benzo[*b*]thiophene (**2a**) (28.1 mg, 60.2 μmol, 1.20 equiv) and methyl *N,N,N',N'*-tetraisopropylphosphorodiamidite (**12**) (13.2 mg, 50.3 μmol) dissolved in THF (1.0 mL) were added tetrabutylammonium fluoride trihydrate (20.7 mg, 65.6 μmol, 1.30 equiv) at 0 °C. After stirring for 1 h at 0 °C, the mixture was filtrated through a pad of Celite® washing with CH_2Cl_2 (0.5 mL × 5), and the filtrate was concentrated under reduced pressure. The residue was purified by preparative TLC (*n*-hexane/EtOAc = 4/1) to give *N,N,N',N'*-tetraisopropyl-*P*-(2,3-dibutylbenzo[*b*]thiophene-6-yl)phosphoric diamide (**13**) (21.6 mg, 43.8 μmol, 87.1%) as a colorless solid.

Colorless solid; Mp 82–83 °C; TLC R_f 0.35 (*n*-hexane/EtOAc = 4/1); ^{1}H NMR ($CDCl_3$, 500 MHz) δ 0.97 (t, 3H+3H, *J* = 7.3 Hz, two signals overlapped, 2 CH_3), 1.11 (d, 12H, *J* = 6.8 Hz, 4 CH_3), 1.31 (d, 12H, *J* = 6.8 Hz, 4 CH_3), 1.45–1.55 (m, 4H, aliphatic), 1.59–1.68 (m, 2H, aliphatic), 1.71–1.80 (m, 2H, aliphatic), 2.77 (t, 2H, *J* = 7.8 Hz, CH_2), 2.87 (t, 2H, *J* = 8.0 Hz, CH_2), 3.62–3.75 (m, 4H, aliphatic), 7.60 (dd, 1H, *J* = 8.4, 3.0 Hz, aromatic), 7.76 (ddd, 1H, *J* = 11.0, 8.4, 1.0 Hz, aromatic), 8.29 (dd, 1H, *J* = 13.0, 1.0 Hz, aromatic); ^{13}C NMR ($CDCl_3$, 126 MHz) δ 13.9 (1C), 14.0 (1C), 22.6 (1C), 23.0 (1C), 23.1–23.4 (m, 4C+4C, two signals overlapped), 26.3 (1C), 28.4 (1C), 32.2 (1C), 33.7 (1C), 46.2 (d, 4C, *J* = 4.9 Hz), 120.3 (d, 1C, *J* = 2.4 Hz), 126.9 (d, 1C, *J* = 10.5 Hz), 127.3 (d, 1C, *J* = 9.8 Hz), 131.4 (d, 1C, *J* = 151.6 Hz), 131.7 (1C), 137.8 (d, 1C, *J* = 16.4 Hz), 141.8 (d, 1C, *J* = 2.5 Hz), 142.9 (1C); ^{31}P NMR (162 MHz) δ 26.0–26.6 (m); IR (KBr, cm^{-1}) 980, 1113, 1138, 1155, 1173, 1188, 1211, 2872, 2930, 2961; HRMS (ESI^+) *m/z* 515.3181 ($[M+Na]^+$, $C_{28}H_{49}N_2NaOPS^+$ requires 515.3195).

The regiochemistry of **13** was determined by the COSY and NOESY experiments.

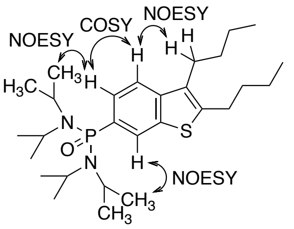

*Synthesis of thioaminated products **15/15'** via the reaction of 6,7-thienobenzyne generated from **2a** with S,S-diphenylsulfilimine **(14)***

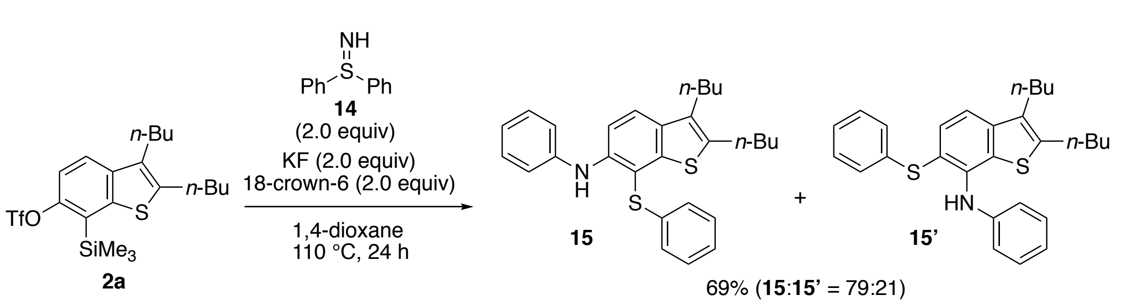

To a mixture of 2,3-dibutyl-6-triflyloxy-7-(trimethylsilyl)benzo[*b*]thiophene (**2a**) (46.6 mg, 0.100 mmol) and *S,S*-diphenylsulfilimine (**14**) (41.0 mg, 0.204 mmol, 2.04 equiv) dissolved in 1,4-dioxane (1.0 mL) were added 18-crown-6 (52.5 mg, 0.199 mmol, 1.99 equiv) and potassium fluoride (11.8 mg, 0.203 mmol, 2.03 equiv) at room temperature, and the mixture was stirred for 24 h with heating at 110 °C (oil bath temperature). After cooling to room temperature, the mixture was filtrated through a pad of Celite® washing with CH_2Cl_2 (0.5 mL × 5), and the filtrate was concentrated under reduced pressure. The residue was purified by preparative TLC (*n*-hexane/EtOAc = 40/1) to give a mixture of 2,3-dibutyl-6-phenylamino-7-(phenylthio)benzo[*b*]thiophene (**15**) and 2,3-dibutyl-7-phenylamino-6-(phenylthio)benzo[*b*]thiophene (**15'**) (30.8 mg, 69.1 μmol, 69.1%, **15**:**15'** = 79:21 as judged from ^{1}H NMR analysis) as an orange oil. For the characterization purpose, the isomers were separated by preparative TLC (*n*-hexane/EtOAc = 20/1) to afford **15** and **15'**.

2,3-Dibutyl-6-phenylamino-7-(phenylthio)benzo[*b*]thiophene (**15**)
Orange oil; TLC R_f 0.42 (*n*-hexane/EtOAc = 20/1); ^{1}H NMR ($CDCl_3$, 500 MHz) δ 0.90–0.99 (m, 6H, 2 CH_3), 1.36–1.46 (m, 4H, aliphatic), 1.52–1.60 (m, 2H, aliphatic), 1.60–1.69 (m, 2H, aliphatic), 2.71 (t, 2H, *J* = 8.0 Hz, aliphatic), 2.77 (t, 2H, *J* = 7.8 Hz, aliphatic), 6.75 (br s, 1H, NH), 6.96–7.01 (AA'BB'C, 1H, aromatic), 7.08–7.16 (m, 5H, aromatic), 7.17–7.22 (AA'BB'C, 2H, aromatic), 7.24–7.29 (AA'BB'C, 2H, aromatic), 7.37 (d, 1H, *J* = 8.8 Hz, aromatic), 7.53 (d, 1H, *J* = 8.8 Hz, aromatic); ^{13}C NMR ($CDCl_3$, 126 MHz) δ 13.9 (1C), 14.0 (1C), 22.5 (1C), 22.9 (1C), 26.3 (1C), 28.1 (1C), 32.4 (1C), 33.6 (1C), 109.2 (1C), 113.2 (1C), 119.9 (2C), 122.2 (1C), 123.4 (1C), 125.8 (1C), 126.6 (2C), 129.1 (2C), 129.3 (2C), 132.3 (1C), 133.9 (1C), 135.0 (1C), 138.1 (1C), 142.5 (1C), 142.7 (1C), 147.6 (1C); IR (KBr, cm^{-1}) 689, 737, 1312, 1377, 1454, 1477, 1504, 1589, 2359, 2857, 2928, 2953, 3057, 3368; HRMS (ESI$^+$) *m/z* 468.1793 ([M+Na]$^+$, $C_{28}H_{31}NNaS_2^+$ requires 468.1790).

The regiochemistry of **15** was determined by the NOESY and the HMBC experiments.

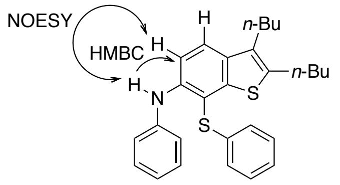

2,3-Dibutyl-7-phenylamino-6-(phenylthio)benzo[*b*]thiophene (**15'**)
Brown solid; Mp 72–74 °C; TLC R_f 0.39 (*n*-hexane/EtOAc = 20/1); ^{1}H NMR ($CDCl_3$, 500 MHz) δ 0.90 (t, 3H, *J* = 7.3 Hz, CH_3), 0.97 (t, 3H, *J* = 7.5 Hz, CH_3), 1.32–1.40 (m, 2H, aliphatic), 1.40–1.48 (m, 2H, aliphatic), 1.55–1.63 (m, 4H, aliphatic), 2.71–2.80 (m, 4H, aliphatic), 6.52 (br s, 1H, NH), 6.76-6.81 (AA'BB'C, 2H, aromatic), 6.91–6.97 (AA'BB'C, 1H, aromatic), 7.07–7.12 (AA'BB'C, 1H, aromatic), 7.12–7.16 (AA'BB'C, 2H, aromatic), 7.16–7.22 (m, 4H, aromatic), 7.35 (d, 1H, *J* = 8.5 Hz, aromatic), 7.51 (d, 1H, *J* = 8.5 Hz, aromatic); ^{13}C NMR ($CDCl_3$, 126 MHz) δ 13.8 (1C), 14.0 (1C), 22.5 (1C), 22.9 (1C), 26.3 (1C), 28.2 (1C), 32.4 (1C), 33.6 (1C), 116.8 (1C), 118.2 (1C), 119.4 (2C), 121.6 (1C), 125.8 (1C), 127.7 (2C), 128.6 (2C), 129.0 (2C), 131.3 (1C), 131.7 (1C), 131.8 (1C), 137.3 (1C), 139.1 (1C), 142.4 (1C), 142.5 (1C), 143.3 (1C); IR (KBr, cm^{-1}) 691, 743, 912, 1393, 1450, 1495, 1599, 2857, 2928, 2955, 3348; HRMS (ESI$^+$) *m/z* 468.1788 ([M+Na]$^+$, $C_{28}H_{31}NNaS_2^+$ requires 468.1790).

The regiochemistry of **15'** was determined by the NOESY and the HMBC experiments.

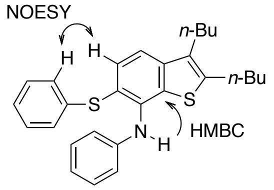

*Synthesis of thioaminated product **17** via the reaction of 6,7-thienobenzyne generated from **2a** with S-(4-tolyl)-S-(4-(trifluoromethyl)phenyl)sulfoximine (**16**)*

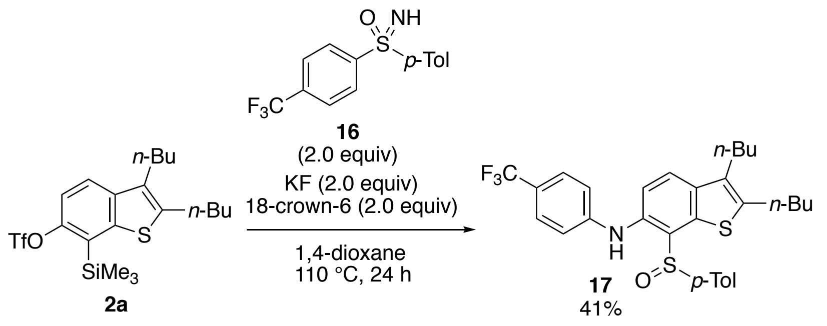

According to the synthetic procedure for **15** and **15'**, 2,3-dibutyl-7-(4-tolylsulfinyl)-6-(4-(trifluoromethyl)-phenylamino)benzo[*b*]thiophene (**17**) was prepared using *S*-(4-tolyl)-*S*-(4-(trifluoromethyl)phenyl)sulfoximine (**16**) instead of **14**.

Pale brown solid; Mp 145–147 °C; TLC R_f 0.59 (CH_2Cl_2); ^{1}H NMR ($CDCl_3$, 500 MHz) δ 0.91–1.02 (m, 6H, 2 CH_3), 1.37–1.52 (m, 4H, aliphatic), 1.51–1.61 (m, 2H, aliphatic), 1.68–1.76 (m, 2H, aliphatic), 2.24 (s, 3H, CH_3), 2.69–2.76 (m, 2H, aliphatic), 2.80–2.92 (m, 2H, aliphatic), 6.88–6.93 (AA'BB', 2H, aromatic), 7.04–7.08 (AA'BB', 2H, aromatic), 7.33 (d, 1H, *J* = 8.7 Hz, aromatic), 7.35–7.40 (AA'BB', 2H, aromatic), 7.45–7.50 (AA'BB', 2H, aromatic), 7.56 (d, 1H, *J* = 8.7 Hz, aromatic), 8.51 (s, 1H, NH); ^{13}C NMR ($CDCl_3$, 126 MHz) δ

13.9 (1C), 14.0 (1C), 21.2 (1C), 22.5 (1C), 22.8 (1C), 26.2 (1C), 28.2 (1C), 32.2 (1C), 33.6 (1C), 116.2 (2C), 117.7 (1C), 122.1 (q, 1C, $^{2}J_{C\text{–}F}$ = 32.8 Hz), 122.8 (1C), 124.1 (2C), 124.5 (q, 1C, $^{1}J_{C\text{–}F}$ = 272.2 Hz), 124.7 (1C), 126.4 (q, 2C, $^{3}J_{C\text{–}F}$ = 3.8 Hz), 129.6 (2C), 131.5 (1C), 136.2 (1C), 139.39 (1C), 139.40 (1C), 139.5 (1C), 139.9 (1C), 141.2 (1C), 145.7 (1C); ^{19}F NMR ($CDCl_3$, 376 MHz) δ –61.8 (s); IR (KBr, cm^{-1}) 1067, 1113, 1165, 1323, 1524, 1597, 1614, 2930, 2957; HRMS (ESI^+) *m/z* 566.1760 ($[M+Na]^+$, $C_{30}H_{32}F_3NNaOS_2^+$ requires 566.1770).

The regiochemistry of **17** was determined by the NOESY experiment.

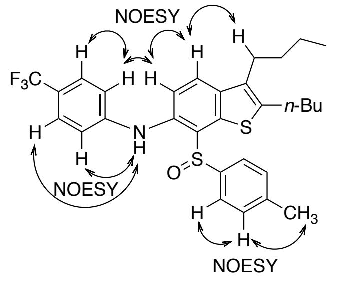

*Synthesis of oxythiolated product **19** via the reaction of 6,7-thienobenzyne generated from **2a** with 2-bromophenyl 4-tolyl sulfoxide (**18**)*

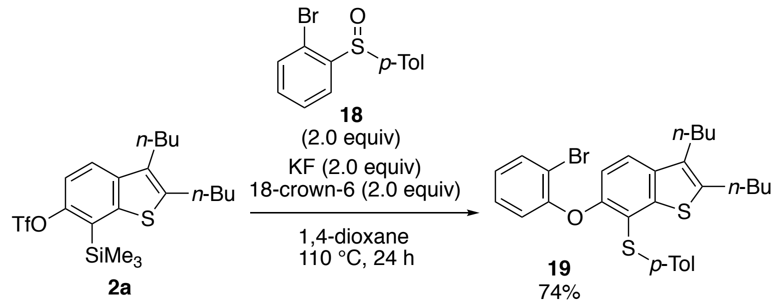

According to the synthetic procedure for **15** and **15'**, 6-(2-bromophenoxy)-2,3-dibutyl-7-(4-tolylthio)-benzo[*b*]thiophene (**19**) was prepared using 2-bromophenyl 4-tolyl sulfoxide (**18**) instead of **14**.

Yellow solid; Mp 44–46 °C; TLC R_f 0.55 (*n*-hexane/CH_2Cl_2 = 3/1); ^{1}H NMR ($CDCl_3$, 500 MHz) δ 0.95 (t, 3H+3H, *J* = 7.3 Hz, two signals overlapped, 2 CH_3), 1.37–1.47 (m, 4H, aliphatic), 1.52–1.61 (m, 2H, aliphatic), 1.64–1.73 (m, 2H, aliphatic), 2.24 (s, 3H, CH_3), 2.73 (t, 2H, *J* = 7.8 Hz, aliphatic), 2.82 (t, 2H, *J* = 7.8 Hz, aliphatic), 6.62 (dd, 1H, *J* = 8.0, 1.3 Hz, aromatic), 6.92 (ddd, 1H, *J* = 8.0, 8.0, 1.3 Hz, aromatic), 6.94 (d, 1H, *J* = 8.5 Hz, aromatic), 6.95–7.00 (AA'BB', 2H, aromatic), 7.11 (ddd, 1H, *J* = 8.0, 8.0, 1.3 Hz, aromatic), 7.22–7.28 (AA'BB', 2H, aromatic), 7.54 (d, 1H, *J* = 8.5 Hz, aromatic), 7.58 (dd, 1H, *J* = 8.0, 1.3 Hz, aromatic); ^{13}C NMR ($CDCl_3$, 126 MHz) δ 13.9 (1C), 14.0 (1C), 21.0 (1C), 22.5 (1C), 22.9 (1C), 26.4 (1C), 28.3 (1C), 32.3 (1C), 33.6 (1C), 113.5 (1C), 116.9 (1C), 118.3 (1C), 118.4 (1C), 122.8 (1C), 124.0 (1C), 128.3 (1C), 129.5 (2C), 130.0 (2C), 131.6 (1C), 132.0 (1C), 133.6 (1C), 136.3 (1C), 137.3 (1C), 141.3 (1C), 146.7 (1C), 153.2 (1C), 154.6 (1C); IR (KBr, cm^{-1}) 746, 1244, 1441, 1449, 1472, 1491, 2928, 2955; Anal. calcd. for $C_{29}H_{31}BrOS_2$: C, 64.55; H, 5.79%; Found: C, 64.63; H, 5.69%.

The regiochemistry of **19** was determined by the NOESY and the HMBC experiments.

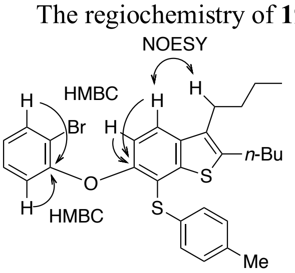

*Synthesis of 6-chloro-7-(dimethylaminocarbonyl)-3-methyl-3H-1,2,3-triazolo[3,4]benzo[1,2-d]thiophene (**21a**)*

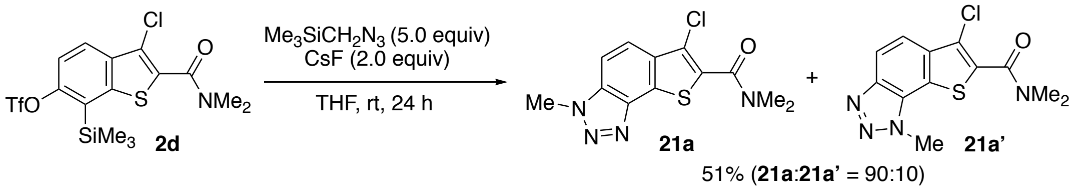

According to the synthetic procedure for **6a** and **6a'**, using 3-chloro-2-dimethylaminocarbonyl-6-triflyloxy-7-(trimethylsilyl)benzo[*b*]thiophene (**2d**) (45.8 mg, 99.6 μmol) and trimethylsilylmethyl azide (64.5 mg, 49.9 μmol) instead of **2a** and **5a**, the mixture of 6-chloro-7-dimethylaminocarbonyl-3-methyl-3*H*-1,2,3-triazolo[3,4]benzo[1,2-*d*]thiophene (**21a**) and 6-chloro-7-dimethylaminocarbonyl-1-methyl-1*H*-1,2,3-triazolo[3,4]benzo[1,2-*d*]thiophene (**21a'**) was obtained (15.1 mg, 51.2 μmol, 51.4%, **21a**:**21a'** = 90:10 as judged from ^{1}H NMR analysis) as a pale yellow solid. 6-Chloro-7-dimethylaminocarbonyl-3-methyl-3*H*-1,2,3-triazolo[3,4]benzo[1,2-*d*]thiophene (**21a**) was identical in spectra data with our previous report.[S2]

Similarly, benzothiophenes **21b** (22.3 mg, 72.9 mmol, 74.0%) and **21c/21c'** (26.5 mg, 81.6 mmol, 80.8%, **21c**:**21c'** = 71:29 as judged from ^{1}H NMR analysis) were prepared from **2d** using furan (33.9 mg, 0.498 mmol) and morpholine (44.9 mg, 0.515 mmol), respectively, instead of trimethylsilylmethyl azide.

3-Chloro-2-dimethylaminocarbonyl-6,9-dihydro-6,9-epoxynaphtho[1,2-*b*]thiophene (**21b**)

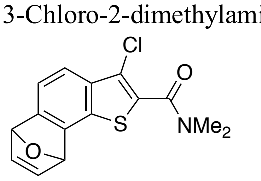

Colorless solid; Mp 49–52 °C; TLC R_f 0.36 (*n*-hexane/EtOAc = 1/2); ^{1}H NMR ($CDCl_3$, 500 MHz) δ 3.08 (br s, 3H, CH_3), 3.17 (br s, 3H, CH_3), 5.88–5.92 (m, 1H, aliphatic), 5.93–5.97 (m, 1H, aliphatic), 7.13 (dd, 1H, *J* = 5.5, 1.5 Hz, olefin), 7.17 (dd, 1H, *J* = 5.5, 1.5 Hz, olefin), 7.46 (d, 1H, *J* = 8.0 Hz, aromatic), 7.53 (d, 1H, *J* = 8.0 Hz, aromatic); ^{13}C NMR ($CDCl_3$, 126 MHz) δ 35.3 (1C), 38.5 (1C), 81.5 (1C), 82.9 (1C), 118.2 (1C), 119.8 (1C), 119.9 (1C), 129.5 (1C), 129.8 (1C), 134.6 (1C), 142.2 (1C), 144.6 (1C), 144.8 (1C), 149.0 (1C), 162.9 (1C); IR (KBr, cm^{-1}) 714, 862, 874, 1188, 1393, 1537, 1634, 1639, 2928; HRMS (ESI^{+}) *m/z* 328.0160 ([M+Na]$^{+}$, $C_{15}H_{12}{}^{35}ClNNaO_2S^{+}$ requires 328.0169).

3-Chloro-2-dimethylaminocarbonyl-6-morpholinobenzo[*b*]thiophene (**21c**)

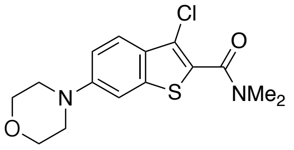

Colorless solid; Mp 165–167 °C; TLC R_f 0.48 (*n*-hexane/EtOAc = 2/1); ^{1}H NMR ($CDCl_3$, 500 MHz) δ 3.05–3.18 (br, 6H, 2 CH_3), 3.21–3.27 (AA'BB', 4H, aliphatic), 3.86–3.93 (AA'BB', 4H, aliphatic), 7.13 (dd, 1H, *J* = 8.9, 2.2 Hz, aromatic), 7.21 (d, 1H, *J* = 2.2 Hz, aromatic), 7.70 (d, 1H, *J* = 8.9 Hz, aromatic); ^{13}C NMR ($CDCl_3$, 126 MHz) δ 35.3 (1C), 38.6 (1C), 49.5 (2C), 66.7 (2C), 107.3 (1C), 116.0 (1C), 119.0 (1C), 122.8 (1C), 126.9 (1C), 129.1 (1C), 139.4 (1C), 150.5 (1C), 163.3 (1C); IR (KBr, cm^{-1}) 743, 912, 1233, 1396, 1599, 1632, 2251, 2859, 2965; Anal. calcd. for $C_{15}H_{17}{}^{35}ClN_2O_2S$: C, 55.47; H, 5.28; N, 8.26%; Found: C, 55.40; H, 5.10; N, 8.50%.

The regiochemistry of **21c** was determined by the COSY and NOESY experiments.

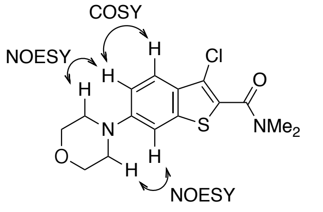

3-Chloro-2-dimethylaminocarbonyl-7-morpholinobenzo[*b*]thiophene (**21c'**)

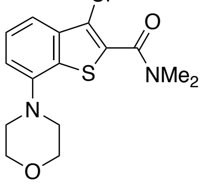

Colorless solid; Mp 114–116 °C; TLC R_f 0.65 (*n*-hexane/EtOAc = 2/1); ^{1}H NMR ($CDCl_3$, 500 MHz) δ 3.08 (br s, 3H, CH_3), 3.17 (br s, 3H, CH_3), 3.17–3.23 (AA'BB', 4H, aliphatic), 3.87–3.94 (AA'BB', 4H, aliphatic), 7.06 (dd, 1H, *J* = 7.7, 0.9 Hz, aromatic), 7.46 (dd, 1H, *J* = 7.7, 7.7 Hz, aromatic), 7.57 (dd, 1H, *J* = 7.7, 0.9 Hz, aromatic); ^{13}C NMR ($CDCl_3$, 126 MHz) δ 35.3 (1C), 38.5 (1C), 51.6 (2C), 67.2 (2C), 115.1 (1C), 117.6 (1C), 119.6 (1C), 126.7 (1C), 130.3 (1C), 131.9 (1C), 137.1 (1C), 147.3 (1C), 163.1 (1C); IR (KBr, cm^{-1}) 743, 912, 1115, 1236, 1261, 1395, 1450, 1634, 2247, 2857, 2961; HRMS (ESI$^+$) *m*/*z* 347.0579 ([M+Na]$^+$, $C_{15}H_{17}{}^{35}ClN_2NaO_2S^+$ requires 347.0591).

The regiochemistry of **21c'** was determined by the COSY and NOESY experiments.

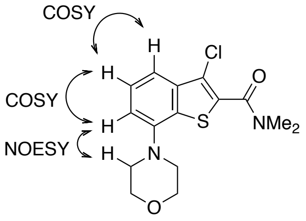

*Synthesis of EP4 antagonist analog **20c***

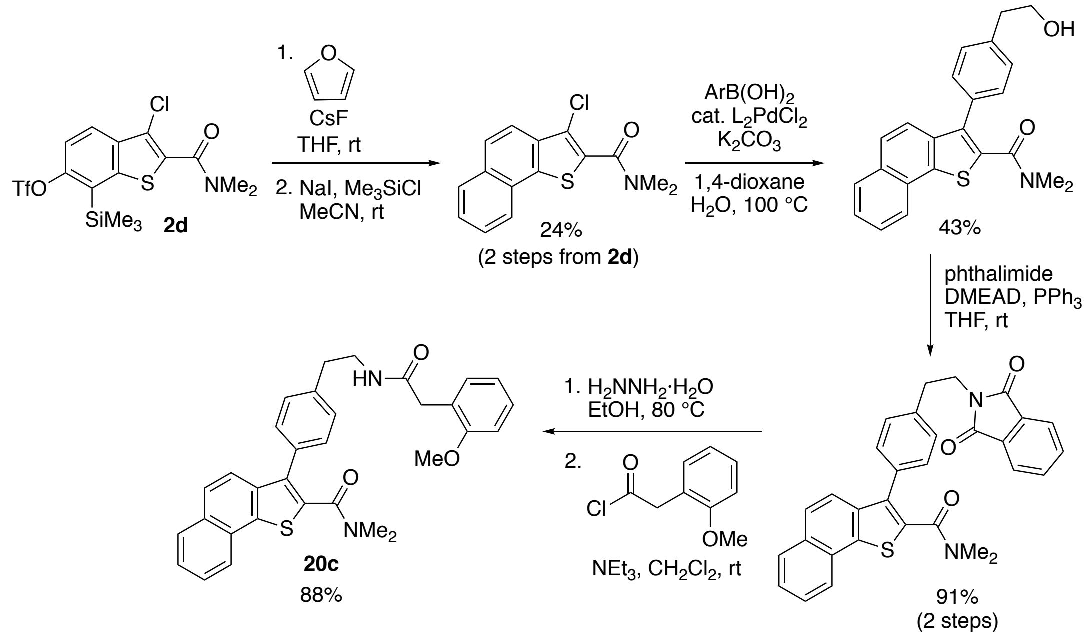

Ar = *p*-($HOCH_2CH_2$)C_6H_4; L = *p*-(Me_2N)C_6H_4P(*t*-Bu)$_2$; DMEAD = di(2-methoxyethyl) azodicarboxylate.

According to the synthetic procedure for **21a** and **21a'**, 3-chloro-2-(dimethylaminocarbnyl)-6,9-dihydro-6,9-epoxynaphtho[1,2-*b*]thiophene (**21b**) was prepared from **2d** (276 mg, 0.600 mmol) using furan (203 mg, 2.98 mmol) instead of trimethylsilylmethyl azide. The mixture was filtrated through a short-pad of silica gel (*n*-hexane/EtOAc = 1/1), and the filtrate was concentrated under reduced pressure. The colorless residue containing 3-chloro-2-dimethylaminocarbonyl-6,9-dihydro-6,9-epoxynaphtho[1,2-*b*]thiophene (**21b**) (154 mg, ca. 0.50 mmol) was used in the next step without further purification.

To a mixture of crude 3-chloro-2-dimethylaminocarbonyl-6,9-dihydro-6,9-epoxynaphtho[1,2-*b*]thiophene (**21b**) (154 mg, ca. 0.50 mmol) and NaI (229 mg, 1.53 mmol, 3.04 equiv) in MeCN (9.2 mL) was added Me_3SiCl (0.20 mL, 1.6 mmol, 3.1 equiv) at room temperature. After stirring for 40 min at the same temperature, to the mixture was added aqueous saturated $Na_2S_2O_3$ (9.2 mL) and the mixture was extracted with EtOAc (10 mL × 3). The combined organic extract was washed with brine (20 mL × 1), dried (Na_2SO_4), and after filtration, the filtrate

was concentrated under reduced pressure. The residue was purified by flash column chromatography (Biotage® SNAP Ultra HP-sphere cartridge 10 g, *n*-hexane/EtOAc = 100/0 to 80/20) to give 3-chloro-2-(dimethylaminocarbonyl)naphtho[1,2-*b*]thiophene (40.9 mg, 0.141 mmol, 23.5% from **2d**) as a colorless solid.

Colorless solid; Mp 154–155 °C; TLC R_f 0.28 (*n*-hexane/EtOAc = 4/1); ^{1}H NMR ($CDCl_3$, 500 MHz) δ 3.14 (br s, 3H, CH_3), 3.20 (br s, 3H, CH_3), 7.58 (ddd, 1H, J = 8.0, 8.0, 0.8 Hz, aromatic), 7.62 (ddd, 1H, J = 8.0, 8.0, 1.5 Hz, aromatic), 7.82 (d, 1H, J = 9.0 Hz, aromatic), 7.85 (d, 1H, J = 9.0 Hz, aromatic), 7.97 (dd, 1H, J = 8.0, 1.5 Hz, aromatic), 8.01 (dd, 1H, J = 8.0, 0.8 Hz, aromatic); ^{13}C NMR ($CDCl_3$, 126 MHz) δ 35.5 (1C), 38.8 (1C), 119.9 (1C), 120.4 (1C), 123.0 (1C), 126.7 (1C), 126.9 (1C), 127.3 (1C), 128.3 (1C), 129.1 (1C), 129.2 (1C), 131.6 (1C), 133.5 (1C), 136.0 (1C), 163.1 (1C); IR (KBr, cm^{-1}) 746, 810, 912, 1194, 1258, 1393, 1506, 1634, 2928; HRMS (ESI$^+$) *m*/*z* 312.0206 ([M+Na]$^+$, $C_{15}H_{12}{}^{35}ClNNaOS^+$ requires 312.0220).

To a mixture of 3-chloro-2-(dimethylaminocarbonyl)naphtho[1,2-*b*]thiophene (25.9 mg, 89.4 μmol), 4-(2-hydroxyethyl)phenylboronic acid (22.5 mg, 0.136 mmol, 1.52 equiv), $(4\text{-}(Me_2N)C_6H_4P(t\text{-}Bu)_2)_2PdCl_2$ (6.5 mg, 9.2 μmol, 10 mol %), and K_2CO_3 (37.2 mg, 0.269 mmol, 3.01 equiv) were added 1,4-dioxane (400 μL) and H_2O (10 μL) at room temperature, and the mixture was heated at 100 °C (oil bath temperature) with stirring for 16 h. After cooling to room temperature, the mixture was filtrated through a pad of Celite® washing with EtOAc (0.5 mL × 5), and the filtrate was concentrated under reduced pressure. The residue was purified by preparative TLC (*n*-hexane/EtOAc = 1/6) to give 2-dimethylaminocarbonyl-3-(4-(2-hydroxyethyl)phenyl)naphtho[1,2-*b*]thiophene (14.6 mg, 38.9 μmol, 43.4%) as a colorless solid.

Colorless solid; Mp 65–68 °C; TLC R_f 0.20 (*n*-hexane/EtOAc = 1/2); ^{1}H NMR ($CDCl_3$, 500 MHz) δ 2.61 (br s, 3H, CH_3), 2.90–3.04 (m, 5H, CH_3+CH_2), 3.92–4.00 (m, 2H, CH_2), 7.35–7.39 (AA'BB', 2H, aromatic), 7.47–7.53 (AA'BB', 2H, aromatic), 7.56 (ddd, 1H, J = 8.0, 8.0, 1.1 Hz, aromatic), 7.61 (ddd, 1H, J = 8.0, 8.0, 1.4 Hz, aromatic), 7.75 (d, 1H, J = 8.8 Hz, aromatic), 7.77 (d, 1H, J = 8.8 Hz, aromatic), 7.94 (dd, 1H, J = 8.0, 1.4 Hz, aromatic), 8.15 (dd, 1H, J = 8.0, 1.1 Hz, aromatic) (the signal for the hydroxy proton was not observed clearly); ^{13}C NMR ($CDCl_3$, 126 MHz) δ 35.2 (1C), 38.5 (1C), 39.0 (1C), 63.6 (1C), 121.5 (1C), 123.7 (1C), 126.0 (1C), 126.4 (1C), 126.9 (1C), 128.7 (1C), 128.8 (1C), 129.4 (2C), 129.5 (2C), 131.3 (1C), 131.7 (1C), 132.6 (1C), 135.5 (1C), 136.6 (1C), 137.5 (1C), 138.6 (1C), 165.4 (1C); IR (KBr, cm^{-1}) 750, 814, 1061, 1261, 1404, 1508, 1620, 2930, 3402; HRMS (ESI$^+$) *m*/*z* 398.1182 ([M+Na]$^+$, $C_{23}H_{21}NNaO_2S^+$ requires 398.1185).

To a mixture of 2-dimethylaminocarbonyl-3-(4-(2-hydroxyethyl)phenyl)naphtho[1,2-*b*]thiophene (9.1 mg, 24 μmol), di(2-methoxyethyl) azodicarboxylate (DMEAD) (8.54 mg, 36.5 μmol, 1.5 equiv), and phthalimide (5.34 mg, 36.3 μmol, 1.5 equiv) dissolved in THF (0.24 mL) was added triphenylphosphine (9.32 mg, 35.5 μmol, 1.5 equiv) at room temperature. After stirring for 16 h at the same temperature, the mixture was concentrated under reduced pressure. The residue was purified by preparative TLC (*n*-hexane/EtOAc = 1/2) to give 2-dimethylaminocarbonyl-3-(4-(2-(1,3-dioxoisoindolin-2-yl)ethyl)phenyl)naphtho[1,2-*b*]thiophene (11.0 mg, 21.8 μmol, 91%) as a colorless solid.

Colorless solid; Mp 166–16 °C; TLC R_f 0.57 (*n*-hexane/EtOAc = 1/2); ^{1}H NMR ($CDCl_3$, 500 MHz) δ 2.50 (br s, 3H, CH_3), 2.92 (br s, 3H, CH_3), 3.10 (t, 2H, J = 7.3 Hz, CH_2), 4.02 (t, 2H, J = 7.3 Hz, CH_2), 7.33–7.38 (AA'BB', 2H, aromatic), 7.42–7.47 (AA'BB', 2H, aromatic), 7.55 (ddd, 1H, J = 7.5, 7.5, 1.2 Hz, aromatic), 7.60 (ddd, 1H, J = 7.5, 7.5, 1.1 Hz, aromatic), 7.70–7.76 (m, 4H, aromatic), 7.81–7.85 (m, 2H, aromatic), 7.93 (dd, 1H, J = 7.5, 1.1 Hz, aromatic), 8.14 (dd, 1H, J = 7.5, 1.2 Hz, aromatic); ^{13}C NMR ($CDCl_3$, 126 MHz) δ 34.3 (1C), 35.0 (1C), 38.3 (1C), 39.2 (1C), 121.4 (1C), 123.2 (2C), 123.7 (1C), 126.0 (1C), 126.4 (1C), 126.9 (1C), 128.7 (1C), 128.8 (1C), 129.38 (2C), 129.42 (2C), 131.3 (1C), 131.9 (1C), 132.0 (2C), 132.9 (1C), 134.0 (2C), 135.3 (1C), 136.5 (1C), 137.6 (1C), 138.2 (1C), 165.3 (1C), 168.1 (2C); IR (KBr, cm^{-1}) 719, 912, 1358, 1395, 1628, 1713, 1771, 2934; HRMS (ESI$^+$) *m*/*z* 527.1409 ([M+Na]$^+$, $C_{31}H_{24}N_2NaO_3S^+$ requires 527.1400).

To a solution of 2-dimethylaminocarbonyl-3-(4-(2-(1,3-dioxoisoindolin-2-yl)ethyl)phenyl)naphtho[1,2-*b*]thiophene (6.8 mg, 13 μmol) in EtOH (0.32 mL) was added hydrazine monohydrate (7.7 μL, 0.16 mmol, 12 equiv) at room temperature and the mixture was heated at 80 °C (oil bath temperature) with stirring for 2 h. After cooling to room temperature, the mixture was concentrated under reduced pressure. To the residue was added water (1 mL) and the mixture was extracted with EtOAc (1 mL × 3). The combined organic extract was washed with brine (1 mL), dried (Na_2SO_4), and after filtration, the filtrate was concentrated under reduced pressure. The residue was dissolved in CH_2Cl_2 (160 μL) and to this solution were added triethylamine (4.4 μL, 32 μmol, 2.5 equiv) and 2-(2-methoxyphenyl)acetyl chloride (0.613 M, CH_2Cl_2 solution, 35 μL, 19 μmol, 1.5 equiv) at room temperature. After stirring for 16 h at the same temperature, the mixture was concentrated under reduced pressure. The residue was purified by preparative TLC (CH_2Cl_2/MeOH = 20/1) to give 2-(dimethylaminocarbonyl)-3-(4-(2-(2-(2-methoxyphenyl)acetamido)ethyl)phenyl)naphtho[1,2-*b*]thiophene (**20c**) (6.2 mg, 12 μmol, 88% in 2 steps from 2-(dimethylaminocarbonyl)-3-(4-(2-(1,3-dioxoisoindolin-2-yl)ethyl)phenyl)naphtho[1,2-*b*]thiophene)

as a colorless solid.

Colorless solid; Mp 59–62 °C; TLC R_f 0.45 (CH_2Cl_2/MeOH = 10/1); ^{1}H NMR ($CDCl_3$, 500 MHz) δ 2.57 (br s, 3H, CH_3), 2.80 (t, 2H, J = 6.8 Hz, CH_2), 2.96 (br s, 3H, CH_3), 3.52 (dt, 2H, J = 6.8, 6.8 Hz, CH_2), 3.58 (s, 2H, CH_2), 3.78 (s, 3H, CH_3), 5.81 (br t, 1H, J = 6.8 Hz, NH), 6.88 (d, 1H, J = 8.0 Hz, aromatic), 6.94 (dd, 1H, J = 8.0, 8.0 Hz, aromatic), 7.15–7.20 (AA'BB', 2H, aromatic), 7.22 (dd, 1H, J = 8.0, 1.6 Hz, aromatic), 7.27 (ddd, 1H, J = 8.0, 8.0, 1.6 Hz, aromatic), 7.39–7.44 (AA'BB', 2H, aromatic), 7.57 (dd, 1H, J = 8.0, 8.0 Hz, aromatic), 7.62 (dd, 1H, J = 8.0, 8.0 Hz, aromatic), 7.72 (d, 1H, J = 8.5 Hz, aromatic), 7.77 (d, 1H, J = 8.5 Hz, aromatic), 7.95 (d, 1H, J = 8.0 Hz, aromatic), 8.16 (d, 1H, J = 8.0 Hz, aromatic); ^{13}C NMR ($CDCl_3$, 126 MHz) δ 35.1 (1C), 35.5 (1C), 38.4 (1C), 38.7 (1C), 40.5 (1C), 55.4 (1C), 110.8 (1C), 121.1 (1C), 121.4 (1C), 123.6 (1C), 123.7 (1C), 125.9 (1C), 126.4 (1C), 127.0 (1C), 128.7 (1C), 128.8 (1C), 128.9 (1C), 129.1 (2C), 129.4 (2C), 131.3 (1C+1C, two signals overlapped), 131.7 (1C), 132.5 (1C), 135.4 (1C), 136.5 (1C), 137.6 (1C), 139.1 (1C), 157.1 (1C), 165.3 (1C), 171.2 (1C); IR (KBr, cm^{-1}) 752, 1246, 1495, 1531, 1628, 2928, 3306; HRMS (ESI$^+$) m/z 545.1865 ([M+Na]$^+$, $C_{32}H_{30}N_2NaO_3S^+$ requires 545.1869).

*Synthesis of EP4 antagonist analog **20d***

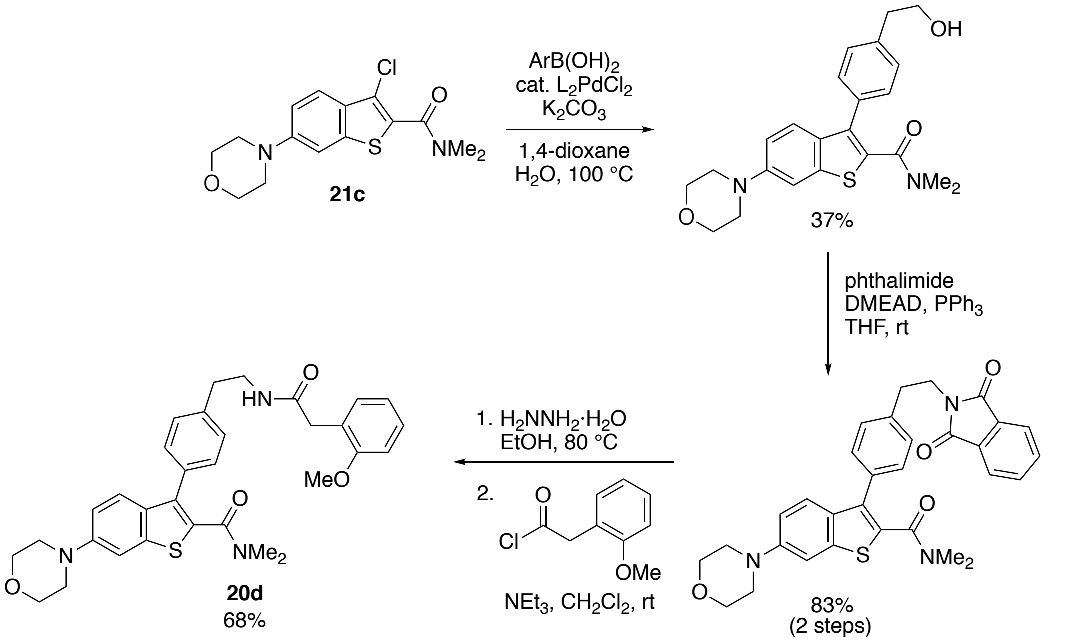

Ar = p-($HOCH_2CH_2$)C_6H_4; L = p-(Me_2N)C_6H_4P(t-Bu)$_2$; DMEAD = di(2-methoxyethyl) azodicarboxylate.

To a mixture of 3-chloro-2-dimethylaminocarbonyl-6-morpholinobenzo[*b*]thiophene (**21c**)(35.2 mg, 0.108 mmol), 4-(2-hydroxyethyl)phenylboronic acid (28.0 mg, 0.169 mmol, 1.56 equiv), (4-(Me_2N)C_6H_4P(t-Bu)$_2$)$_2PdCl_2$ (8.2 mg, 12 μmol, 11 mol %), and K_2CO_3 (47.2 mg, 0.342 mmol, 3.17 equiv) were added 1,4-dioxane (0.43 mL) and H_2O (10 μL) at room temperature and the mixture was heated at 100 °C (oil bath temperature) with stirring for 16 h. After cooling to room temperature, the mixture was filtrated through a pad of Celite® washing with EtOAc (0.5 mL × 10), and the filtrate was concentrated under reduced pressure. The residue was purified by preparative TLC (n-hexane/EtOAc = 1/5) to give 2-dimethylaminocarbonyl-3-(4-(2-hydroxyethyl)phenyl)-6-morpholinobenzo[*b*]thiophene (16.5 mg, 40.2 μmol, 37.2%) as a colorless solid.

Colorless solid; Mp 82–84 °C; TLC R_f 0.13 (n-hexane/EtOAc = 1/6); ^{1}H NMR ($CDCl_3$, 500 MHz) δ 2.56 (br s, 3H, CH_3), 2.87–2.98 (m, 5H, CH_3+CH_2), 3.20–3.26 (AA'BB', 4H, aliphatic), 3.86–3.92 (AA'BB', 4H, aliphatic), 3.90–3.96 (br, 2H, CH_2), 7.05 (dd, 1H, J = 9.0, 2.0 Hz, aromatic), 7.31 (d, 1H, J = 2.0 Hz, aromatic), 7.29–7.35 (AA'BB', 2H, aromatic), 7.41–7.46 (AA'BB', 2H, aromatic), 7.66 (d, 1H, J = 9.0 Hz, aromatic) (the signal for the hydroxy proton was not observed clearly); ^{13}C NMR ($CDCl_3$, 126 MHz) δ 35.1 (1C), 38.4 (1C), 39.0 (1C), 49.7 (2C), 63.5 (1C), 66.8 (2C), 107.5 (1C), 115.7 (1C), 124.0 (1C), 129.2 (2C), 129.4 (2C), 129.6 (1C), 131.4 (1C), 132.7 (1C), 134.9 (1C), 138.5 (1C), 141.4 (1C), 149.7 (1C), 165.6 (1C); IR (KBr, cm^{-1}) 731, 951, 1034, 1121, 1233, 1398, 1449, 1537, 1601, 2241, 2857, 2926, 3406; HRMS (ESI$^+$) m/z 433.1544 ([M+Na]$^+$, $C_{23}H_{26}N_2NaO_3S^+$ requires 433.1556).

To a solution of 2-dimethylaminocarbonyl-3-(4-(2-hydroxyethyl)phenyl)-6-morpholinobenzo[*b*]thiophene (16.5 mg, 40.3 μmol), di(2-methoxyethyl) azodicarboxylate (DMEAD) (14.8 mg, 63.2 μmol, 1.57 equiv), and

phthalimide (8.9 mg, 61 μmol, 1.5 equiv) dissolved in THF (0.40 mL) was added triphenylphosphine (17.0 mg, 64.8 μmol, 1.61 equiv) at room temperature. After stirring for 16 h at the same temperature, the mixture was concentrated under reduced pressure. The residue was purified by preparative TLC (*n*-hexane/EtOAc = 1/5) to give 2-dimethylaminocarbonyl-3-(4-(2-(1,3-dioxoisoindolin-2-yl)ethyl)phenyl)-6-morpholinobenzo[*b*]thiophene (18.1 mg, 33.5 μmol, 83.1%) as a pale yellow solid.

Pale yellow solid; Mp 82–84 °C; TLC R_f 0.38 (*n*-hexane/EtOAc = 1/6); ^{1}H NMR ($CDCl_3$, 500 MHz) δ 2.46 (br s, 3H, CH_3), 2.87 (br s, 3H, CH_3), 3.08 (t, 2H, J = 7.3 Hz, CH_2), 3.20–3.27 (AA'BB', 4H, aliphatic), 3.85–3.93 (AA'BB', 4H, aliphatic), 4.00 (t, 2H, J = 7.3 Hz, CH_2), 7.05 (dd, 1H, J = 9.0, 2.0 Hz, aromatic), 7.28–7.33 (m, 3H, aromatic), 7.36–7.40 (AA'BB', 2H, aromatic), 7.62 (d, 1H, J = 9.0 Hz, aromatic), 7.68–7.74 (m, 2H, aromatic), 7.79–7.85 (m, 2H, aromatic); ^{13}C NMR ($CDCl_3$, 126 MHz) δ 34.2 (1C), 34.9 (1C), 38.2 (1C), 39.2 (1C), 49.7 (2C), 66.8 (2C), 107.5 (1C), 115.7 (1C), 123.2 (2C), 123.9 (1C), 129.2 (2C), 129.3 (2C), 129.8 (1C), 131.3 (1C), 132.0 (2C), 133.0 (1C), 134.0 (2C), 134.8 (1C), 138.0 (1C), 141.4 (1C), 149.7 (1C), 165.4 (1C), 168.1 (2C); IR (KBr, cm^{-1}) 741, 912, 1121, 1233, 1395, 1599, 1620, 1713, 2857, 2957; HRMS (ESI$^+$) *m/z* 562.1756 ([M+Na]$^+$, $C_{31}H_{29}N_3NaO_4S^+$ requires 562.1771).

To a solution of 2-dimethylaminocarbonyl-3-(4-(2-(1,3-dioxoisoindolin-2-yl)ethyl)phenyl)-6-morpholino-benzo[*b*]thiophene (8.22 mg, 15.2 μmol) in EtOH (0.31 mL) was added hydrazine monohydrate (7.4 μL, 0.15 mmol, 10 equiv) at room temperature and the mixture was heated at 80 °C (oil bath temperature) with stirring for 2 h. After cooling to room temperature, the mixture was concentrated under reduced pressure. To the residue was added water (1 mL) and the mixture was extracted with EtOAc (2 mL × 5). The combined organic extract was washed with brine (1 mL × 3), dried (Na_2SO_4), and after filtration, the filtrate was concentrated under reduced pressure. The residue was dissolved in CH_2Cl_2 (0.15 mL) and to this solution were added triethylamine (4.2 μL, 30 μmol, 2.0 equiv) and 2-(2-methoxyphenyl)acetyl chloride (0.613 M, CH_2Cl_2 solution, 34 μL, 18 μmol, 1.2 equiv) at room temperature. After stirring for 6 h at the same temperature, the mixture was concentrated under reduced pressure. The residue was purified by preparative TLC (CH_2Cl_2/MeOH = 20/1) to give 2-dimethylamino-carbonyl-3-(4-(2-(2-(2-methoxyphenyl)acetamido)ethyl)phenyl)-6-morpholinobenzo[*b*]thiophene (**20d**) (5.8 mg, 10 μmol, 68% in 2 steps from 2-dimethylaminocarbonyl-3-(4-(2-(1,3-dioxoisoindolin-2-yl)ethyl)phenyl)-6-morpholinobenzo[*b*]thiophene) as a colorless solid.

Colorless solid; Mp 72–75 °C; TLC R_f 0.54 (CH_2Cl_2/MeOH = 10/1); ^{1}H NMR ($CDCl_3$, 500 MHz) δ 2.52 (br s, 3H, CH_3), 2.77 (t, 2H, J = 6.8 Hz, CH_2), 2.92 (br s, 3H, CH_3), 3.23–3.28 (AA'BB', 4H, aliphatic), 3.50 (dt, 2H, J = 6.8, 6.8 Hz, CH_2), 3.56 (s, 2H, CH_2), 3.76 (s, 3H, CH_3), 3.87–3.94 (AA'BB', 4H, aliphatic), 5.79 (br t, 1H, J = 6.8 Hz, NH), 6.87 (dd, 1H, J = 7.8, 1.0 Hz, aromatic), 6.93 (ddd, 1H, J = 7.8, 7.8, 1.0 Hz, aromatic), 7.07 (dd, 1H, J = 9.0, 2.0 Hz, aromatic), 7.11–7.16 (AA'BB', 2H, aromatic), 7.20 (dd, 1H, J = 7.8, 1.6 Hz, aromatic), 7.26 (ddd, 1H, J = 7.8, 7.8, 1.6 Hz, aromatic), 7.32 (d, 1H, J = 2.0 Hz, aromatic), 7.33–7.38 (AA'BB', 2H, aromatic), 7.62 (d, 1H, J = 9.0 Hz, aromatic); ^{13}C NMR ($CDCl_3$, 126 MHz) δ 35.0 (1C), 35.5 (1C), 38.3 (1C), 38.7 (1C), 40.5 (1C), 49.8 (2C), 55.4 (1C), 66.8 (2C), 107.5 (1C), 110.7 (1C), 115.6 (1C), 121.1 (1C), 123.6 (1C), 123.9 (1C), 128.8 (1C), 129.06 (2C), 129.13 (2C), 129.7 (1C), 131.3 (1C), 131.4 (1C), 132.7 (1C), 134.8 (1C), 138.9 (1C), 141.4 (1C), 149.7 (1C), 157.1 (1C), 165.5 (1C), 171.2 (1C); IR (KBr, cm^{-1}) 743, 912, 1233, 1246, 1601, 1622, 2241, 2857, 2926, 3308; HRMS (ESI$^+$) *m/z* 580.2234 ([M+Na]$^+$, $C_{32}H_{35}N_3NaO_4S^+$ requires 580.2240).

Affinity determination of benzothiophene derivatives 20a–d to human EP4 receptor

(a) Specific binding of [^{3}H]PGE$_2$ to the membrane of HEK293 cells expressing human EP4 receptor. Curve fitting shows that K_d value is 6.6 nM. (b) Competition binding of [^{3}H]PGE$_2$ by benzothiophene derivatives **20a**–**d**. The K_i values were calculated using the K_d value determined in (a). Data are presented as mean ± s.e.m. of triplicate experiments.

(a)

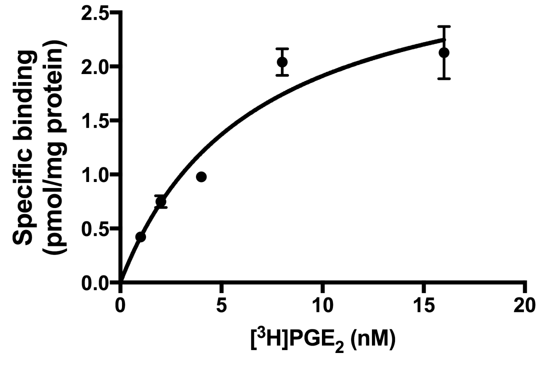

(b)

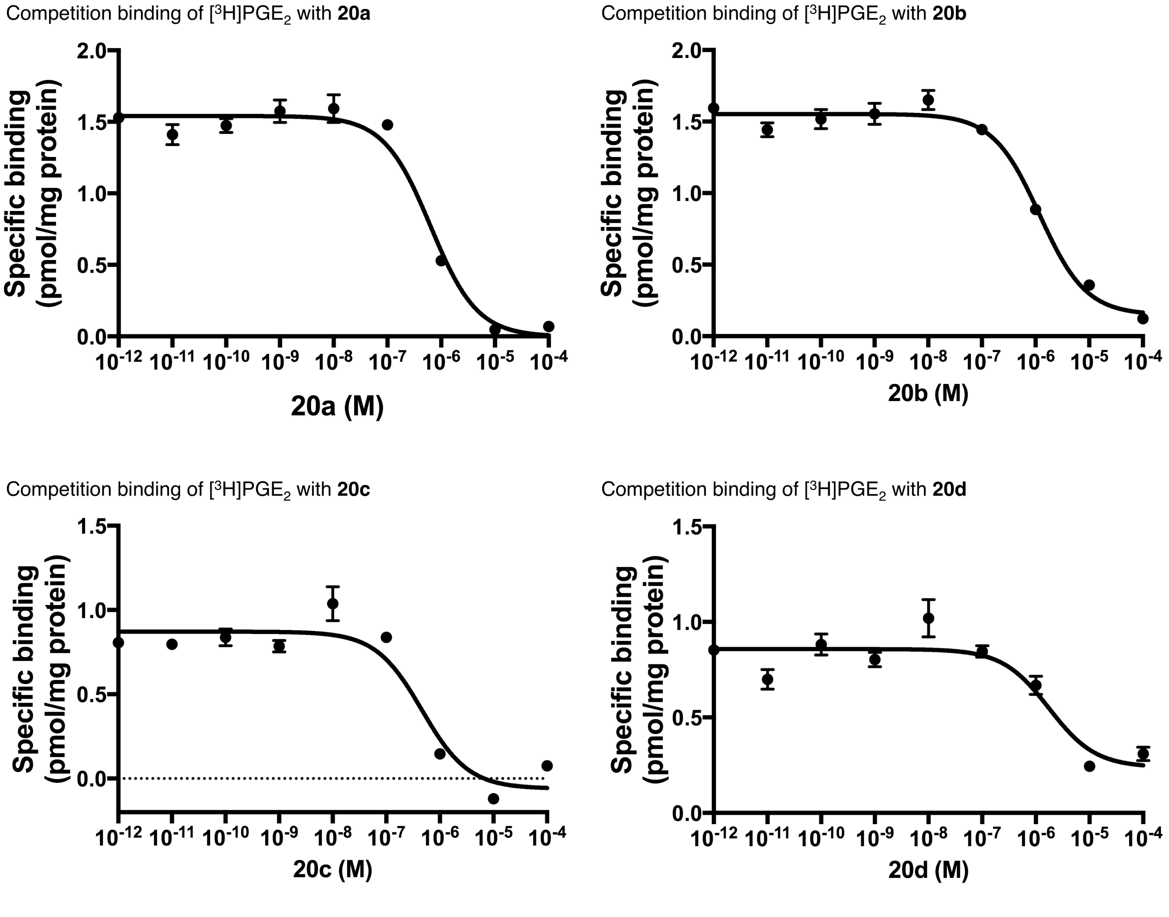

Materials and methods of radioligand binding assays

Wild-type human EP4 cDNA was subcloned into the mammalian expression vector pcDNA3.1 (Thermo Fisher Scientific), and the resultant plasmid was transfected into HEK293 cells using Lipofectamine 2000 (Thermo Fisher Scientific). After culture for 24 h, the cells were harvested, and homogenized in a buffer comprising 25 mM Tris-HCl (pH 7.5), containing 0.25 M sucrose, 10 mM $MgCl_2$, 1 mM EDTA, and 0.1 mM phenylmethylsulfonyl fluoride. The homogenate was centrifuged at 100,000 g for 30 min, and the pellet was suspended in binding buffer (20 mM MES [pH 6.0], 10 mM $MgCl_2$ and 1 mM EDTA), and used as crude membranes in binding assays. Protein concentrations were determined using the BCA assay (Thermo Fisher Scientific).

[^{3}H]PGE_2 (PerkinElmer) affinity (K_d) was determined by saturation biding assay, performed by incubating varying concentrations of [^{3}H]PGE_2 with 50 μg of crude membranes at 30 ºC for 1 h, in a final volume of 100 μL binding buffer. Non-specific binding was determined using a 1,000-fold excess of non-radiolabelled PGE_2. Radioligand inhibition binding assays were performed by co-incubating 50 μg of crude membranes with 10 nM [^{3}H]PGE_2 and varying concentrations of non-radiolabelled test compounds. After the reaction, the mixture was rapidly filtered through Whatman GF/B glass filters presoaked in 0.3% (v/v) polyethylenimine. The filter was then washed twice with 5 mL of ice-cold K–P buffer (1.32 mM K_2HPO_4, 8.68 mM KH_2PO_4, 10 mM $MgCl_2$, 1 mM EDTA). The radioactivity associated with filter was measured in 2.5 mL of Clearsol I scintillation liquid (Nacalai Tesque) by an AccuFLEX LCS-8000 liquid scintillation counter (Hitachi). Specific binding was calculated by subtracting nonspecific binding from total binding. All binding assay measurements were analyzed using Prism (GraphPad).

References for Supporting Information

S1 D. E. Bergbreiter and E. Pendergrass, *J. Org. Chem.*, 1981, **46**, 219.

S2 T. Morita, S. Yoshida, M. Kondo, T. Matsushita and T. Hosoya, *Chem. Lett.*, 2017, **46**, 81.

S3 E. A. Wydysh, S. M. Medghalchi, A. Vadlamudi and C. A. Townsend, *J. Med. Chem.*, 2009, **52**, 3317.

S4 S. Yoshida, H. Nakajima, K. Uchida, T. Yano, M. Kondo, T. Matsushita and T. Hosoya, *Chem. Lett.*, 2017, **46**, 77.

S5 T. Matsuzawa, K. Uchida, S. Yoshida and T. Hosoya, *Org. Lett.*, 2017, **19**, 5521.

^{1}H and ^{13}C NMR Spectra of Compounds

^{1}H NMR (500 MHz) and ^{13}C NMR (126 MHz) spectra of 2,3-dibutyl-6-(isopropylaminocarbonyloxy)-benzo[*b*]thiophene ($CDCl_3$)

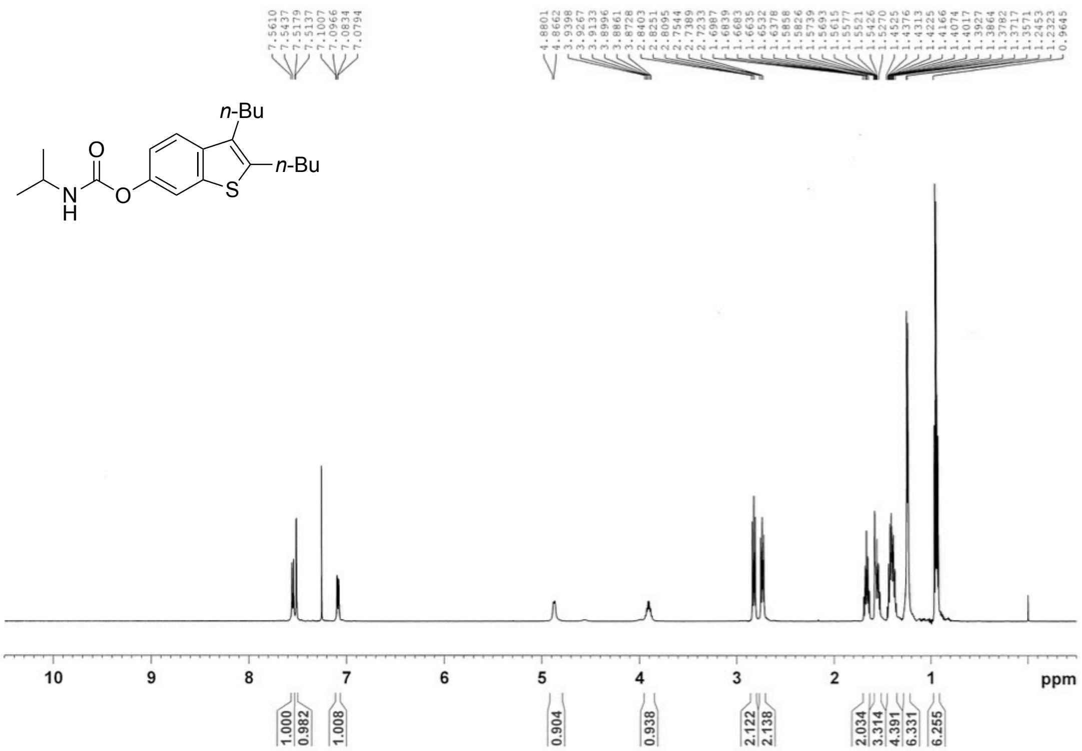

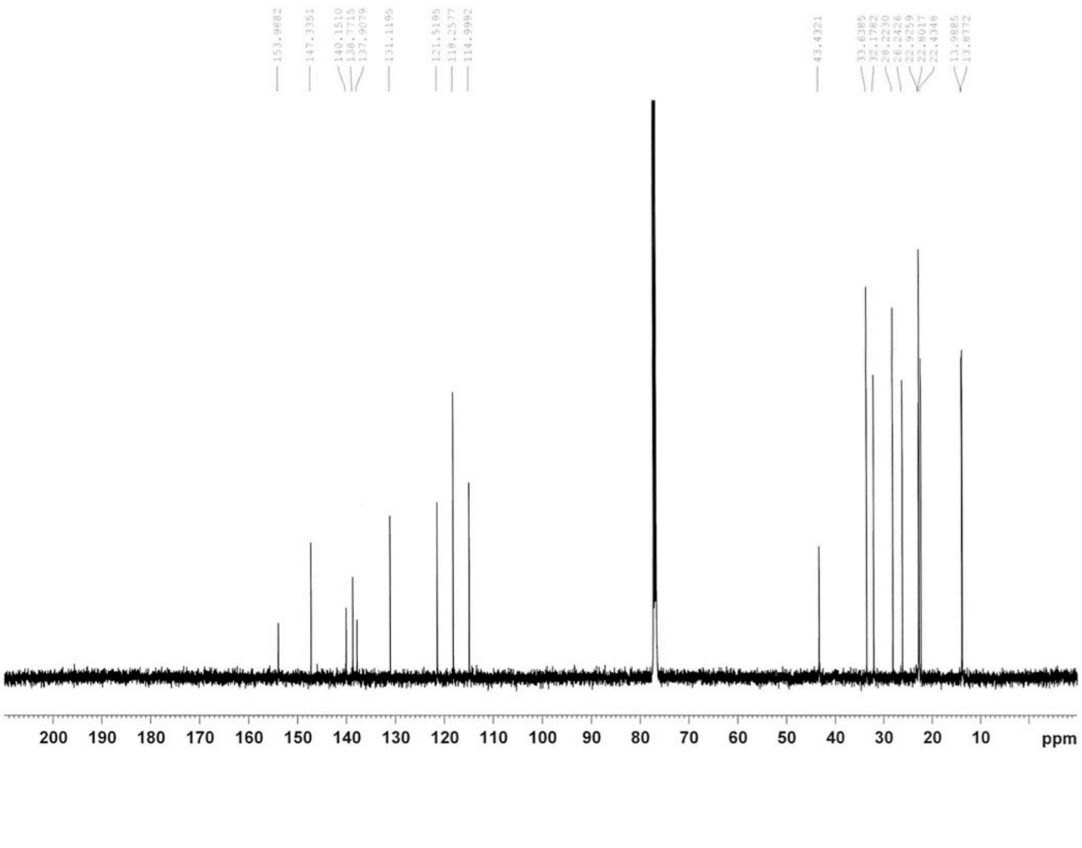

^{1}H NMR (500 MHz) and ^{13}C NMR (126 MHz) spectra of 2,3-dibutyl-6-isopropylaminocarbonyloxy-7-(trimethylsilyl)benzo[*b*]thiophene ($CDCl_3$)

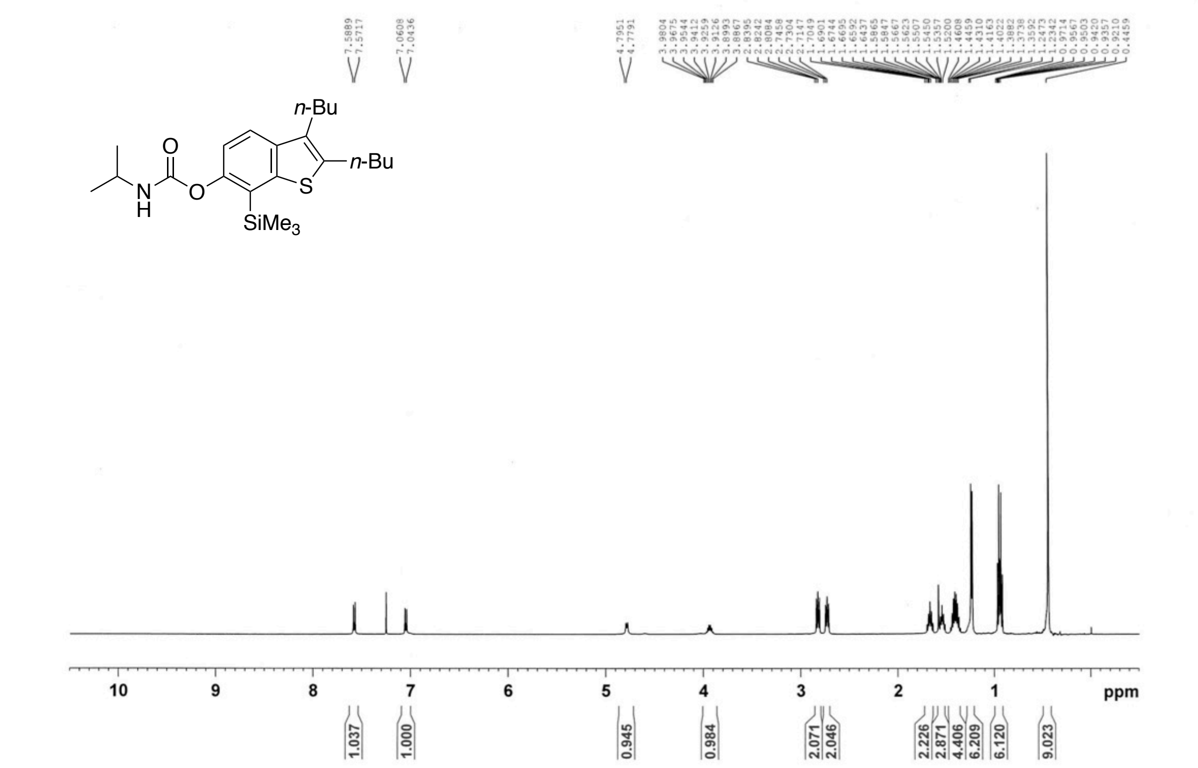

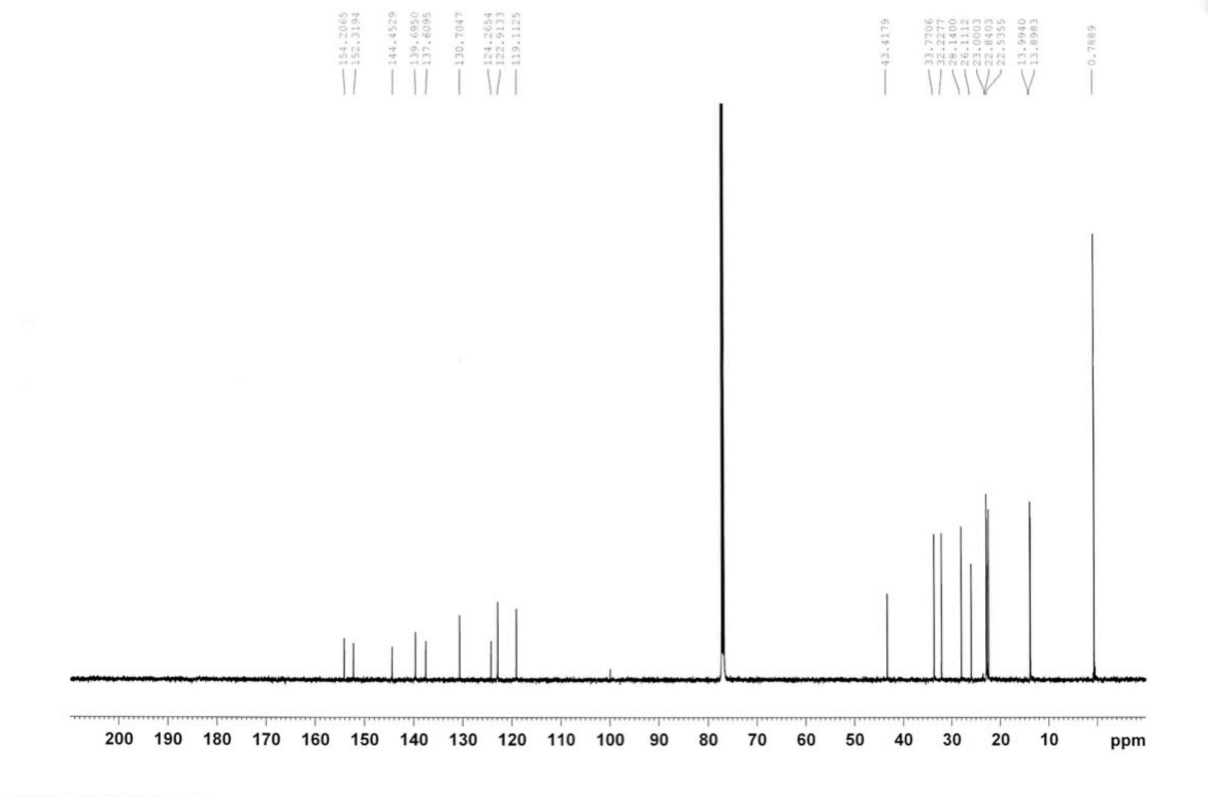

^{1}H NMR (500 MHz) and ^{13}C NMR (126 MHz) spectra of **2a** ($CDCl_3$)

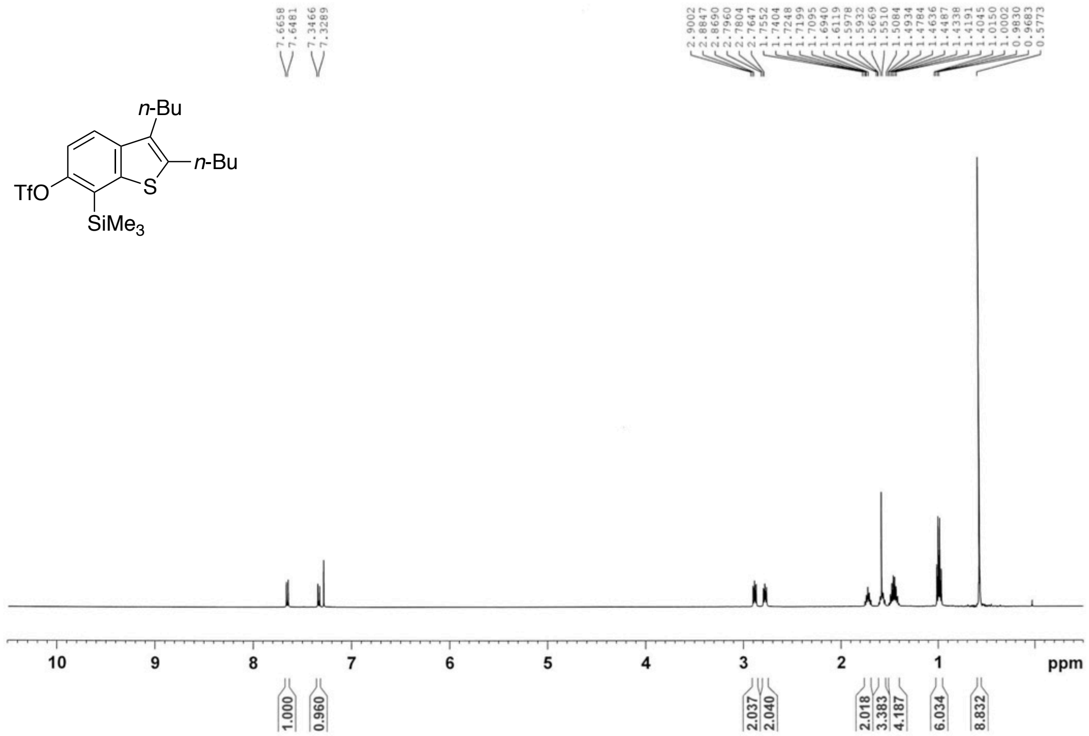

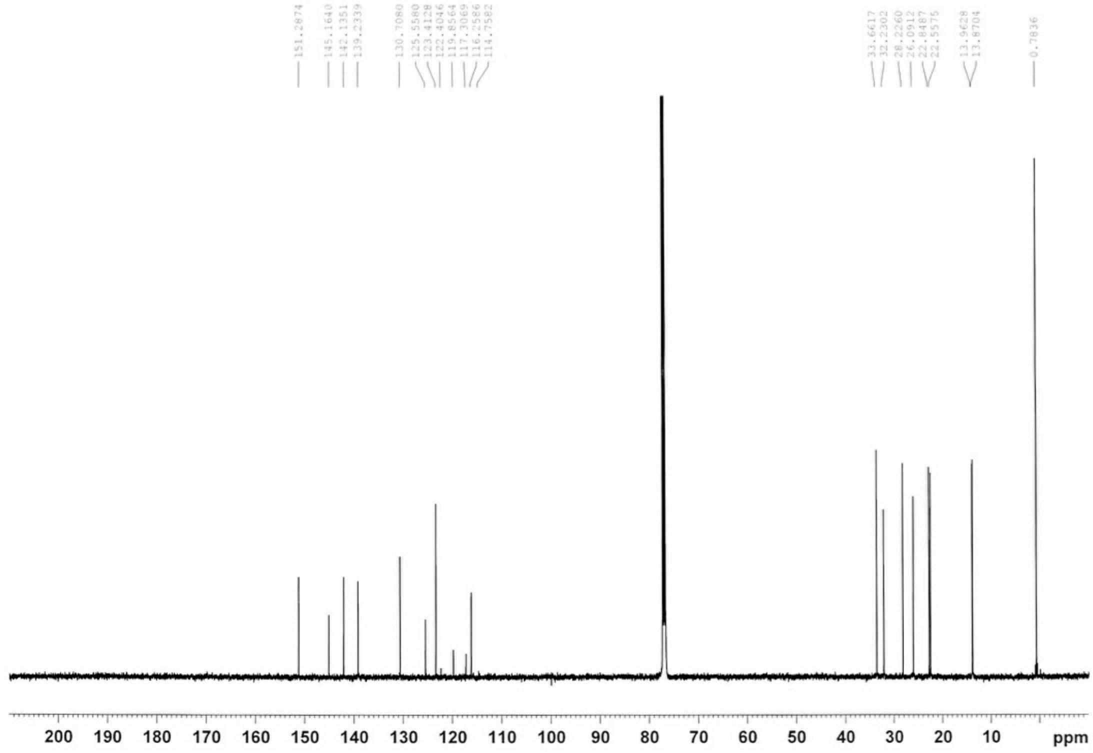

^{1}H NMR (500 MHz) and ^{13}C NMR (126 MHz) spectra of 3-methyl-6-isopropylaminocarbonyloxy-2-phenyl-benzo[*b*]thiophene ($CDCl_3$)

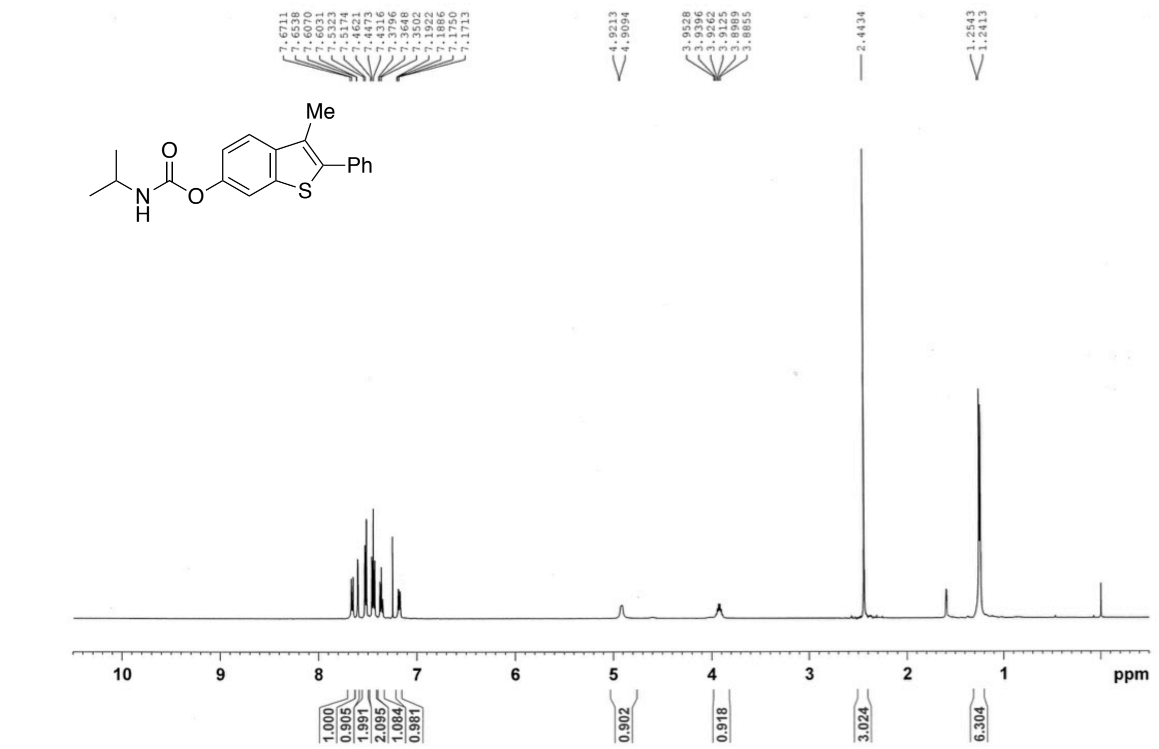

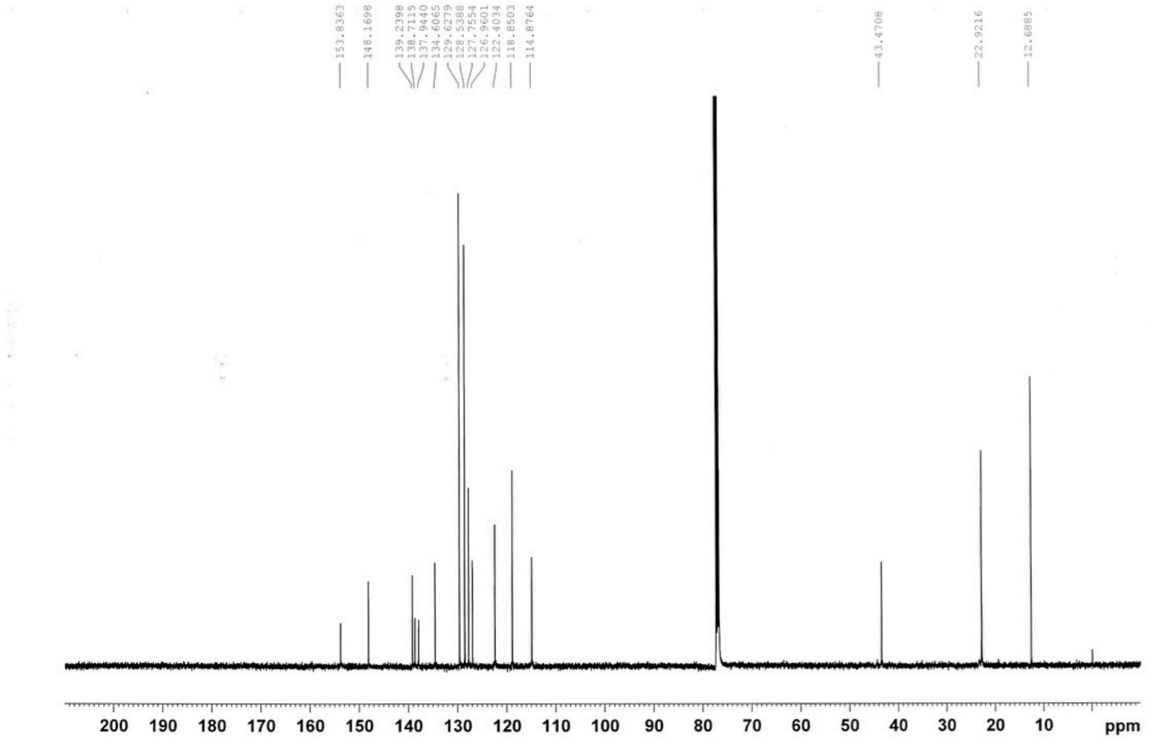

^{1}H NMR (500 MHz) and ^{13}C NMR (126 MHz) spectra of 3-methyl-6-isopropylaminocarbonyloxy-2-phenyl-7-(trimethylsilyl)benzo[*b*]thiophene ($CDCl_3$)

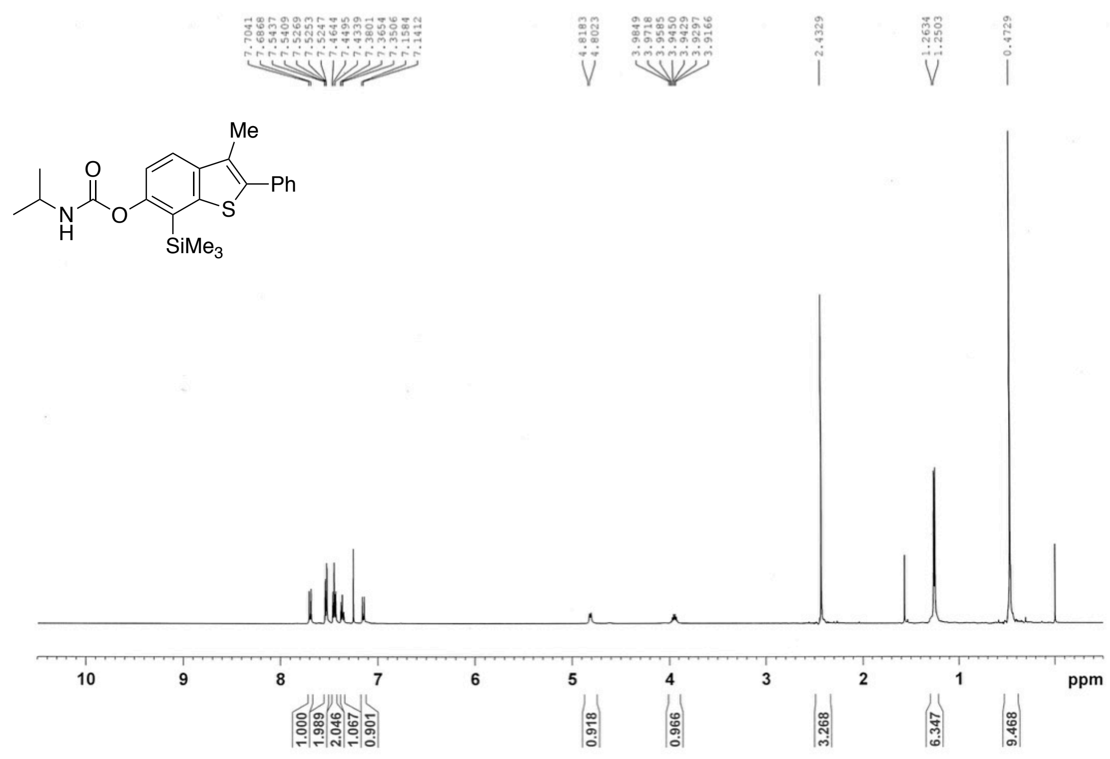

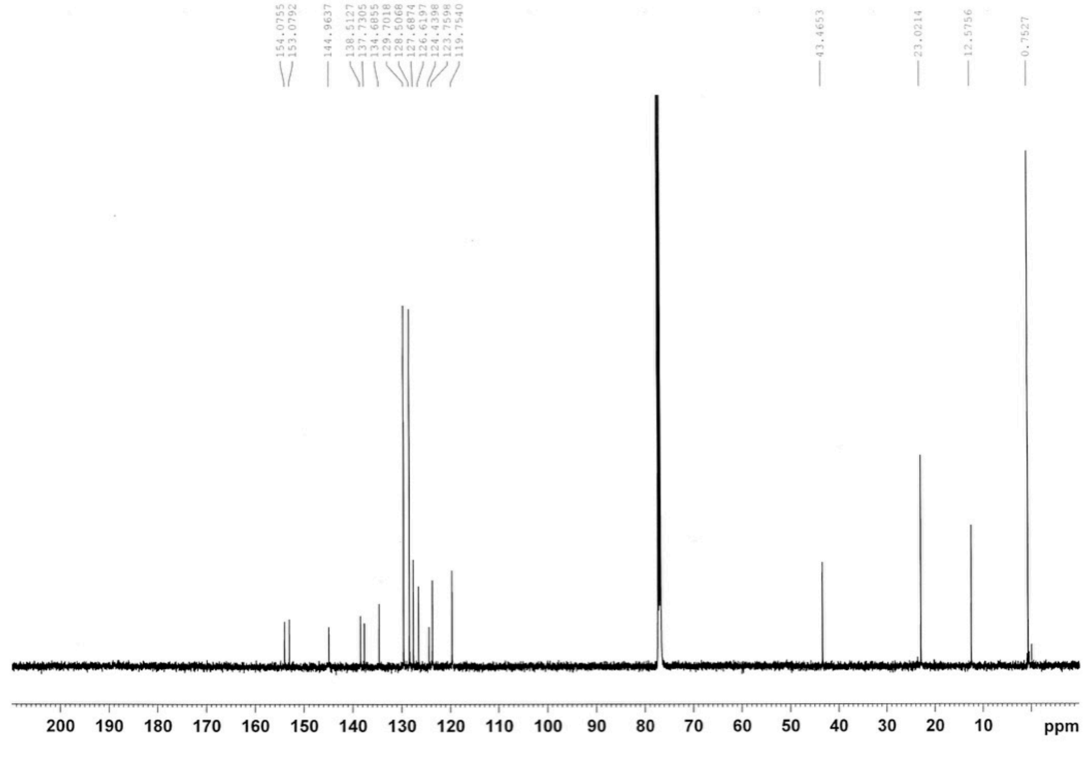

^{1}H NMR (500 MHz) and ^{13}C NMR (126 MHz) spectra of **2b** ($CDCl_3$)

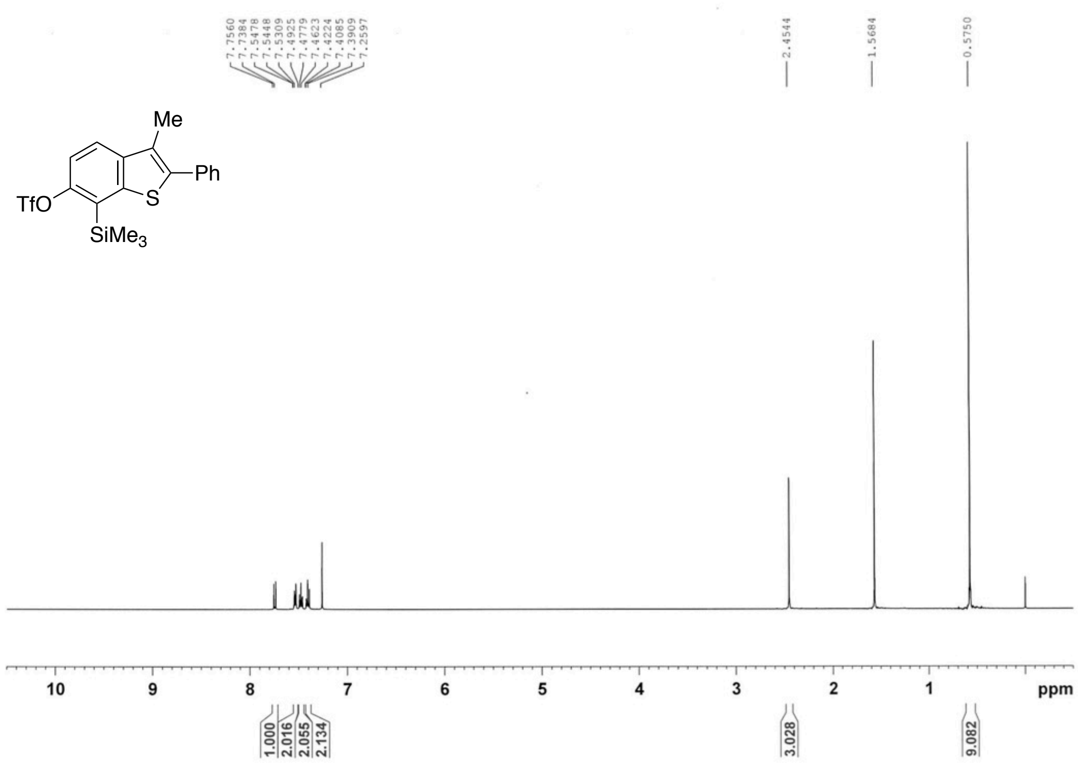

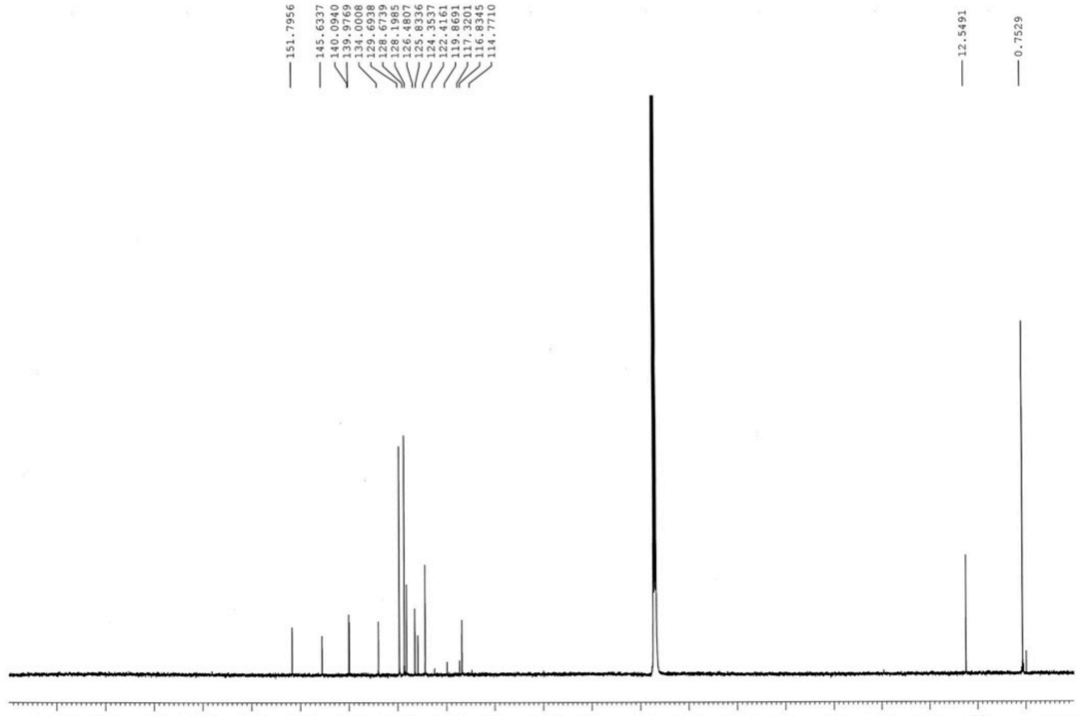

^{1}H NMR (500 MHz) and ^{13}C NMR (126 MHz) spectra of 6-isopropylaminocarbonyloxy-2-methylthio-3-(trifluoromethyl)benzo[*b*]thiophene (CDCl3)

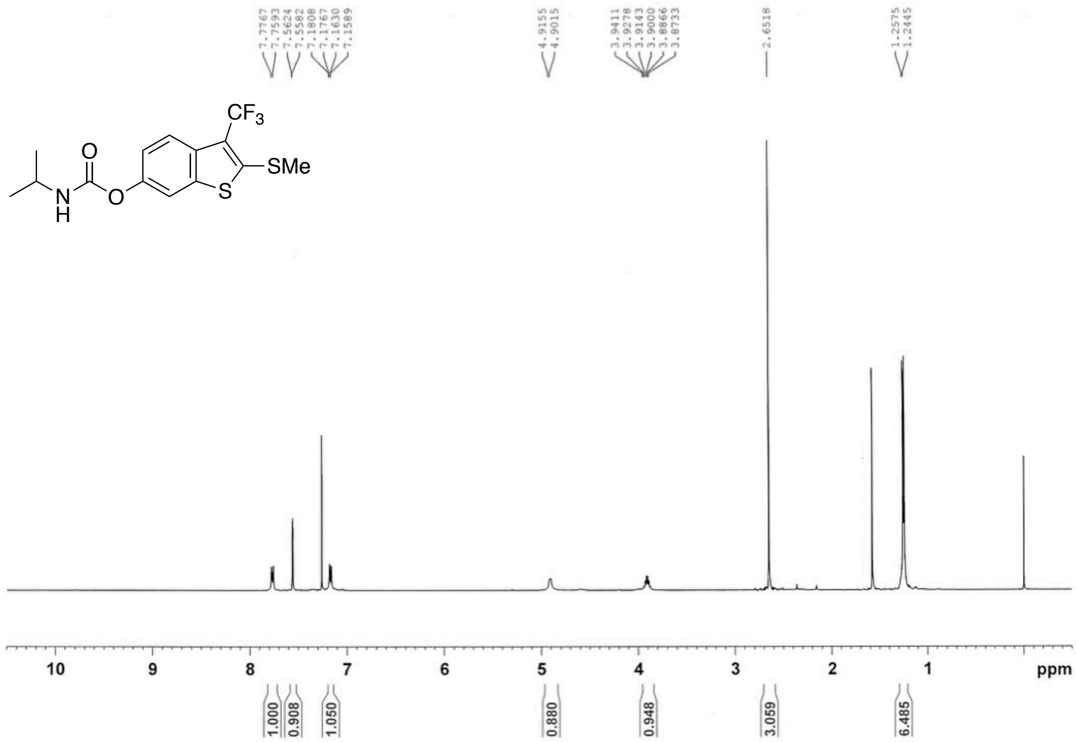

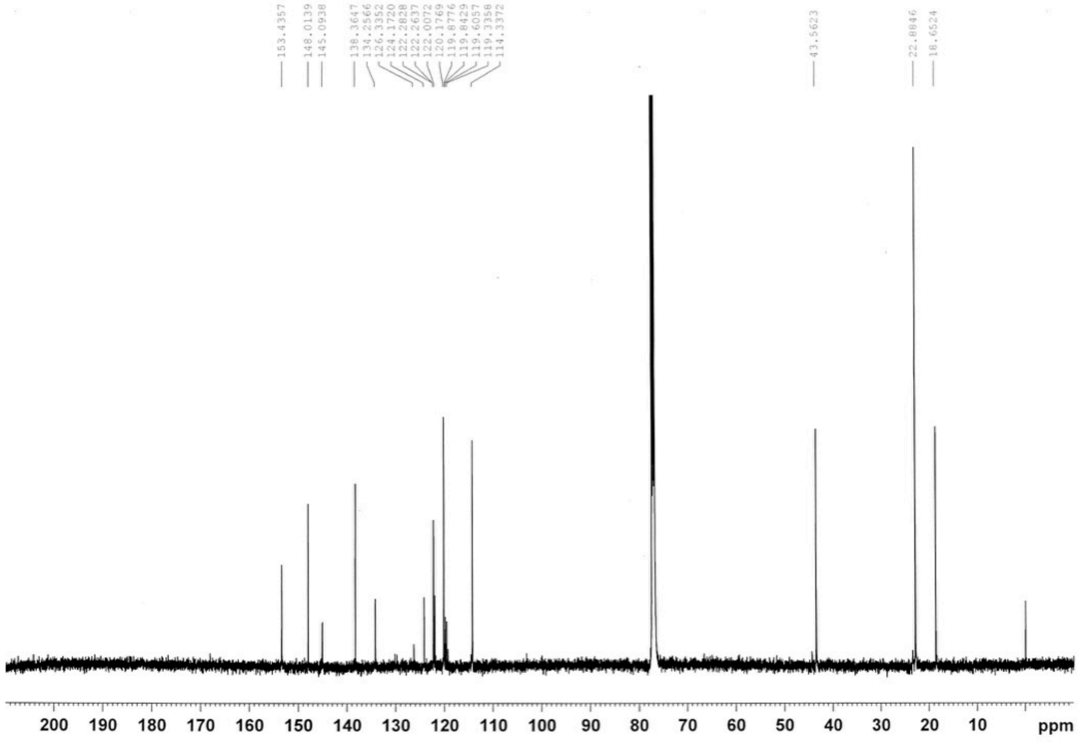

^{1}H NMR (500 MHz) and ^{13}C NMR (126 MHz) spectra of 6-isopropylaminocarbonyloxy-2-methylthio-3-trifluoromethyl-7-(trimethylsilyl)benzo[*b*]thiophene ($CDCl_3$)

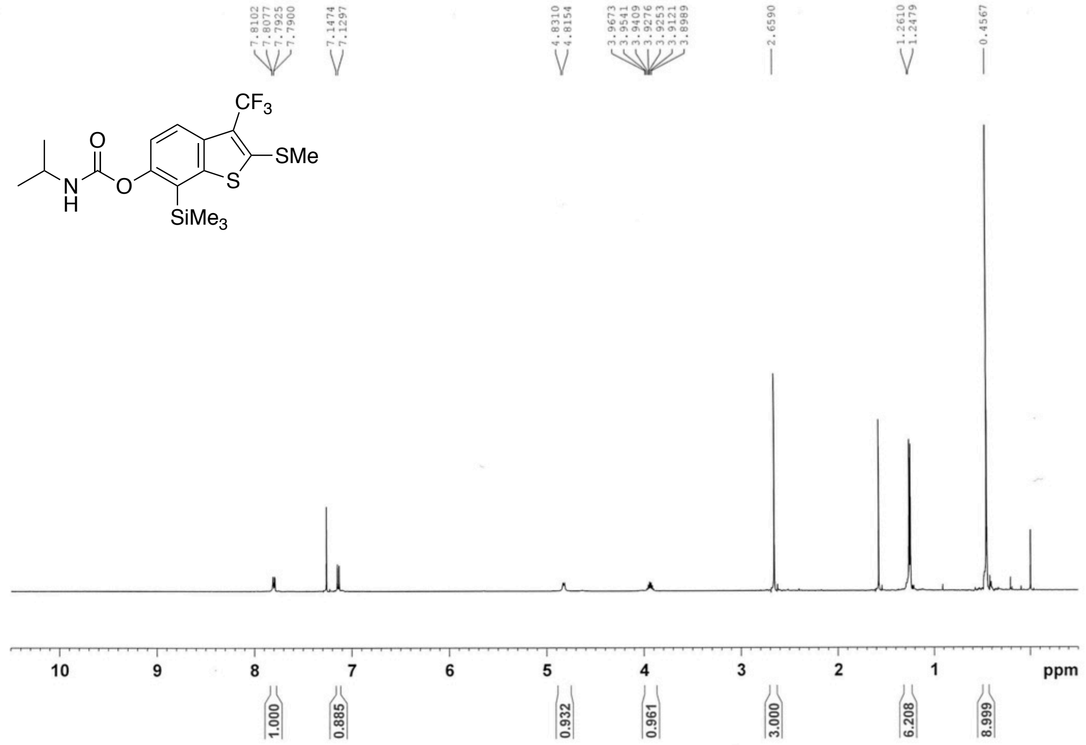

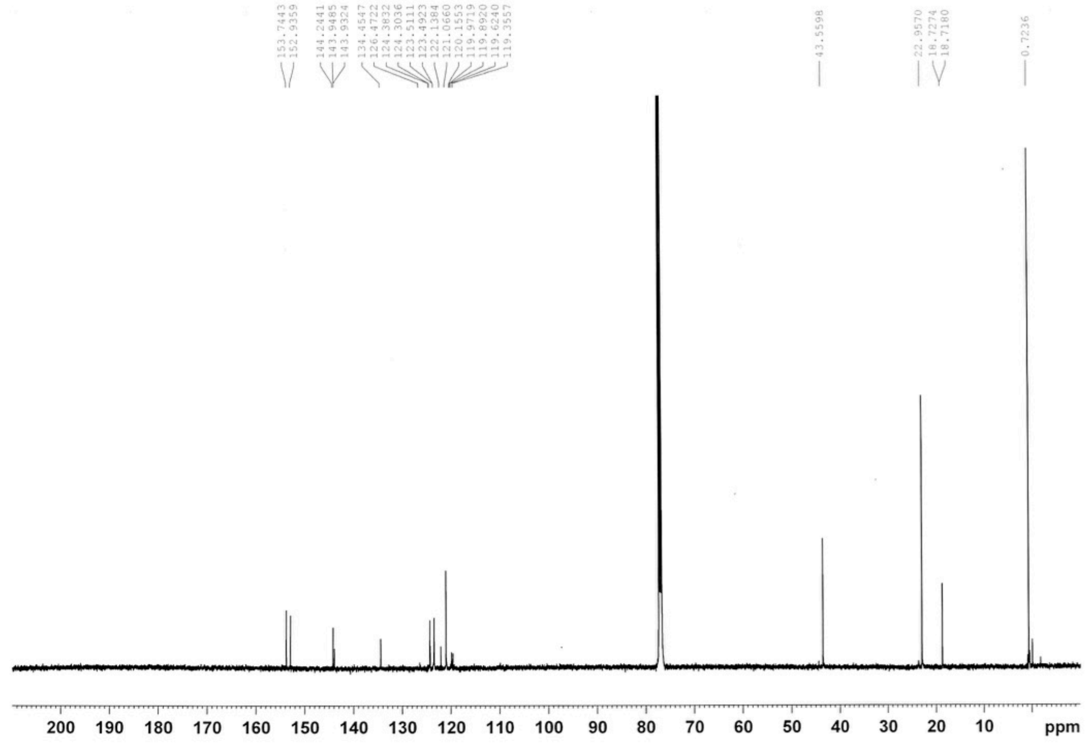

^{1}H NMR (500 MHz) and ^{13}C NMR (126 MHz) spectra of **2c** ($CDCl_3$)

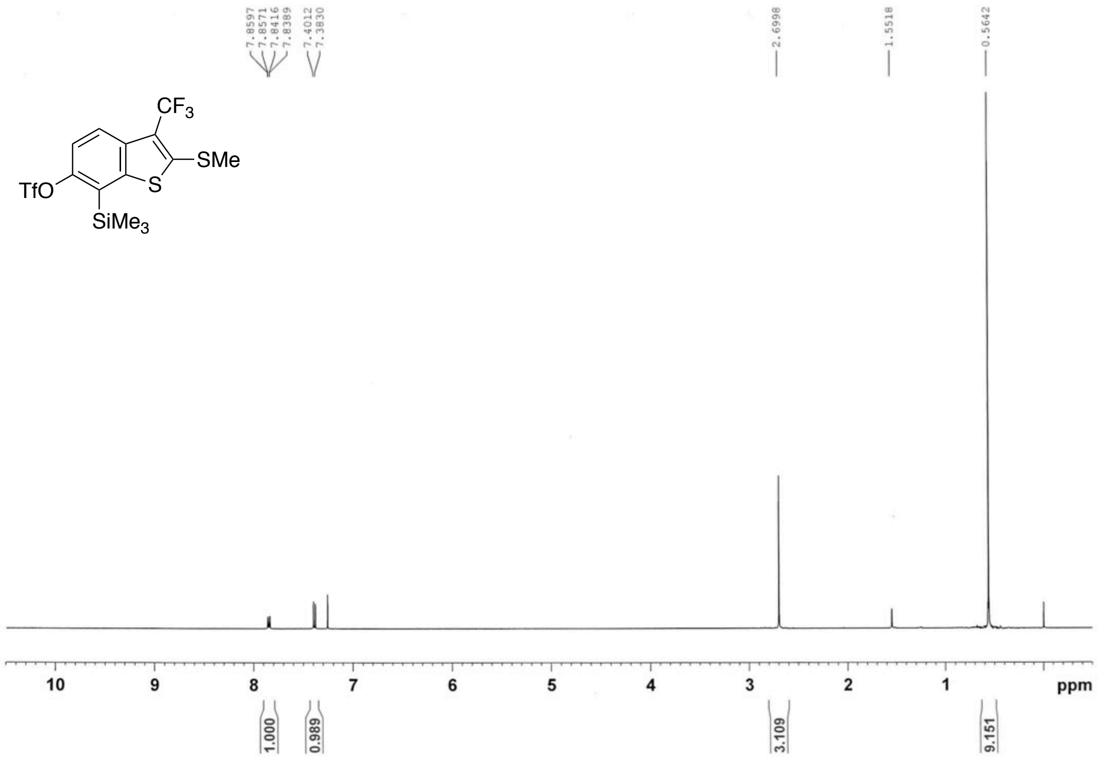

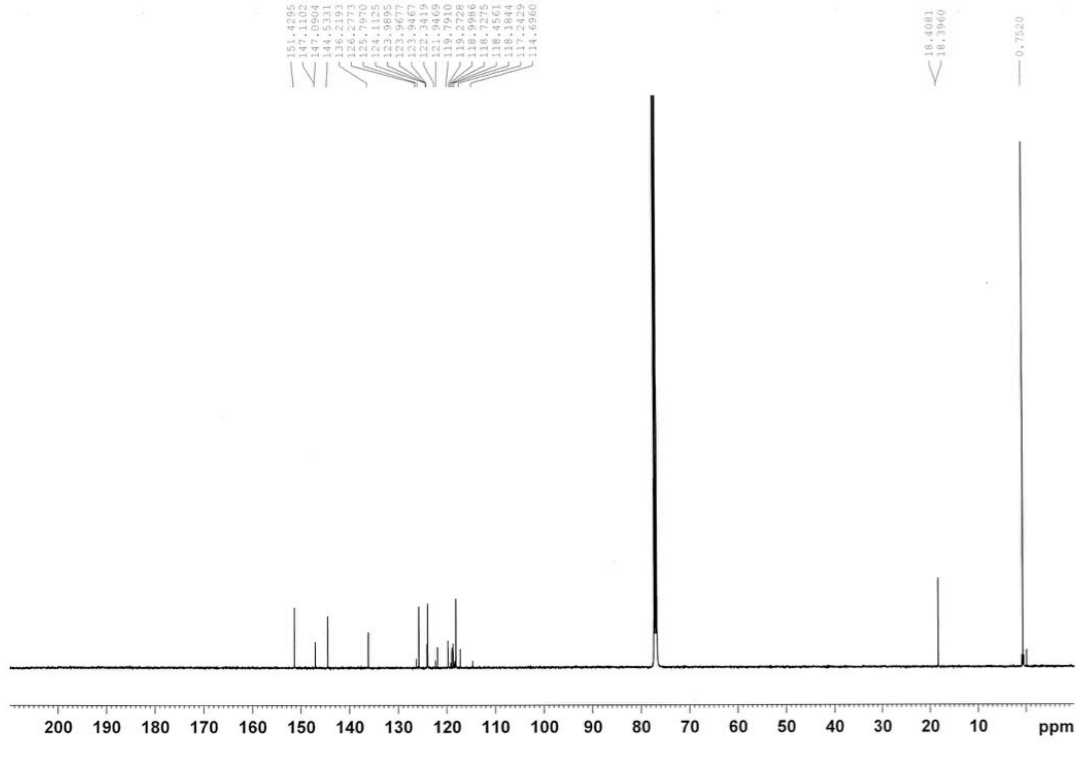

^{1}H NMR (500 MHz) and ^{13}C NMR (126 MHz) spectra of **3** (DMSO-d_6)

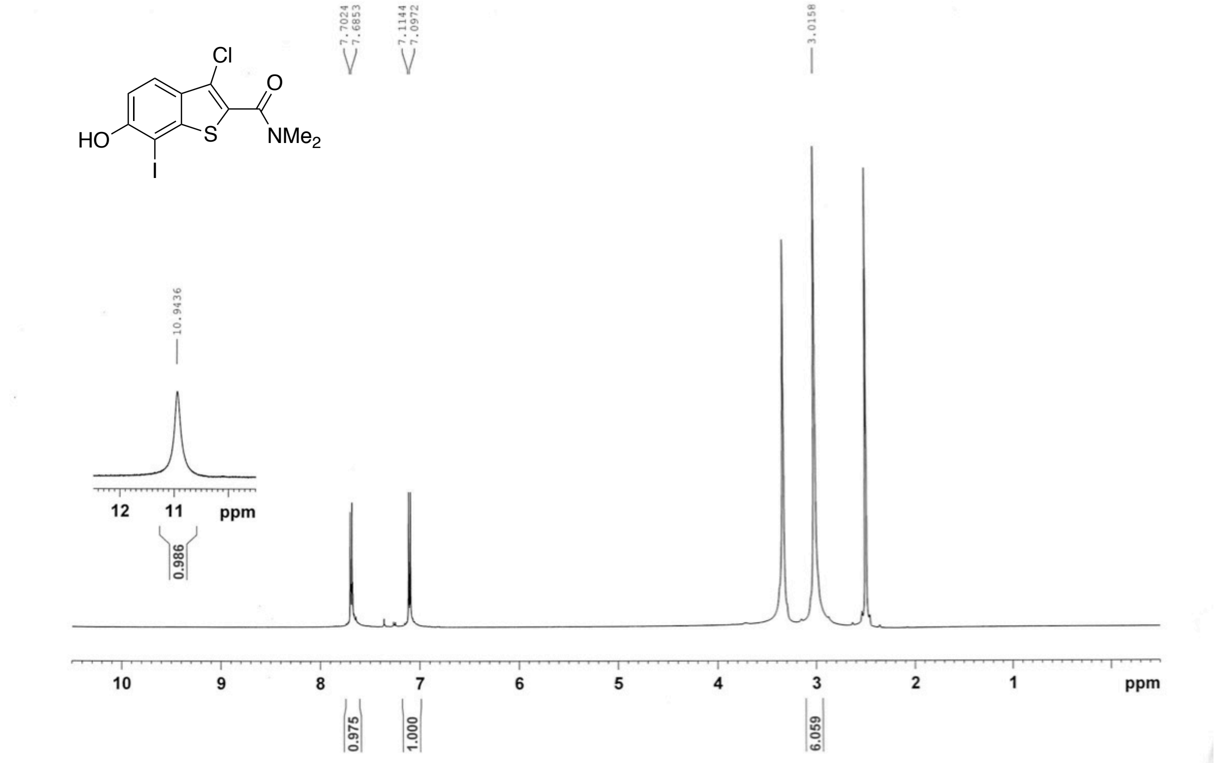

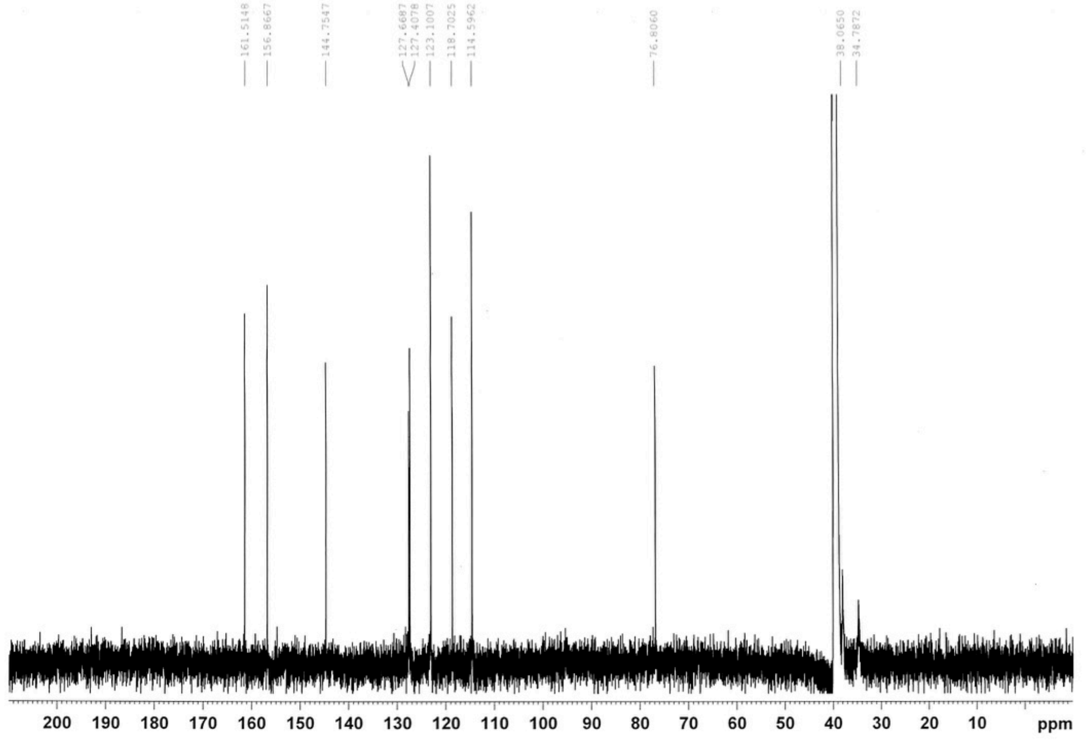

^{1}H NMR (500 MHz) and ^{13}C NMR (126 MHz) spectra of **4** ($CDCl_3$)

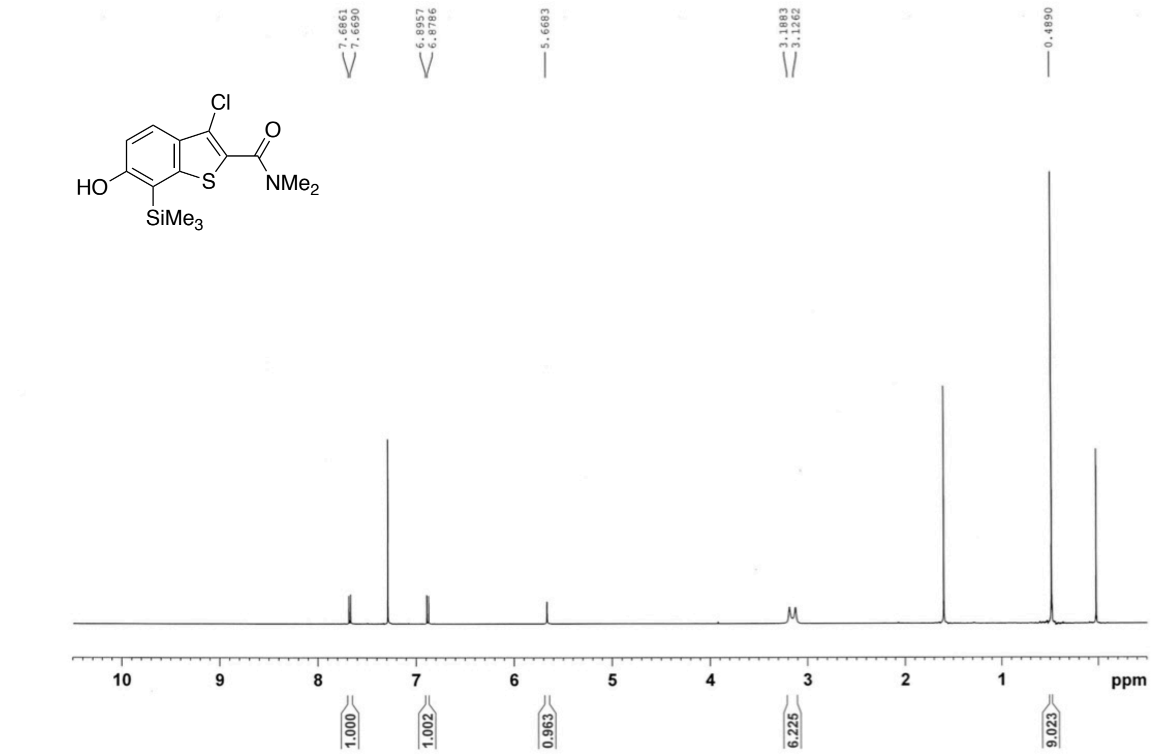

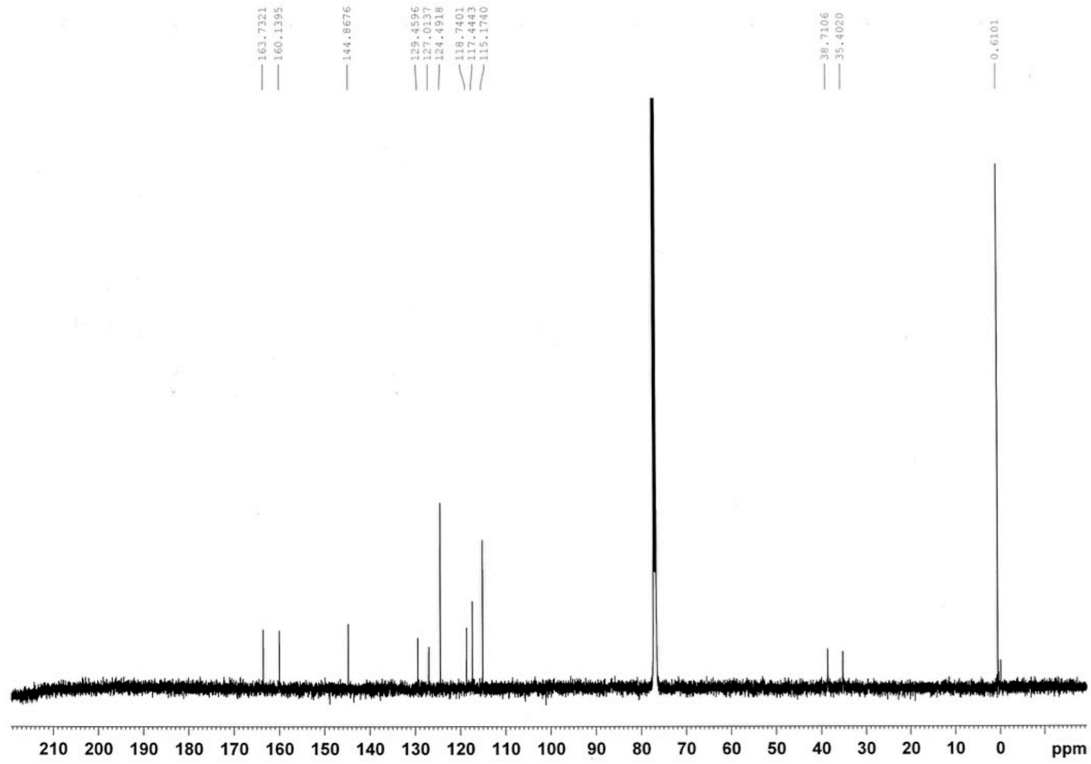

^{1}H NMR (500 MHz) and ^{13}C NMR (126 MHz) spectra of **2d** ($CDCl_3$)

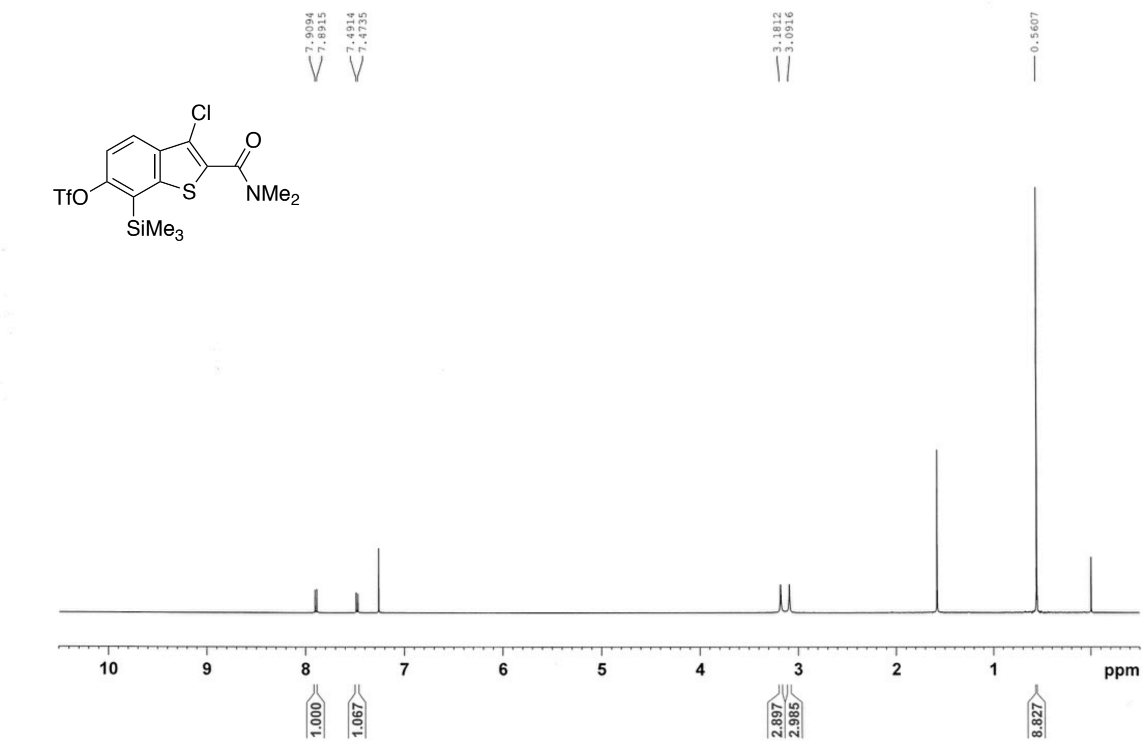

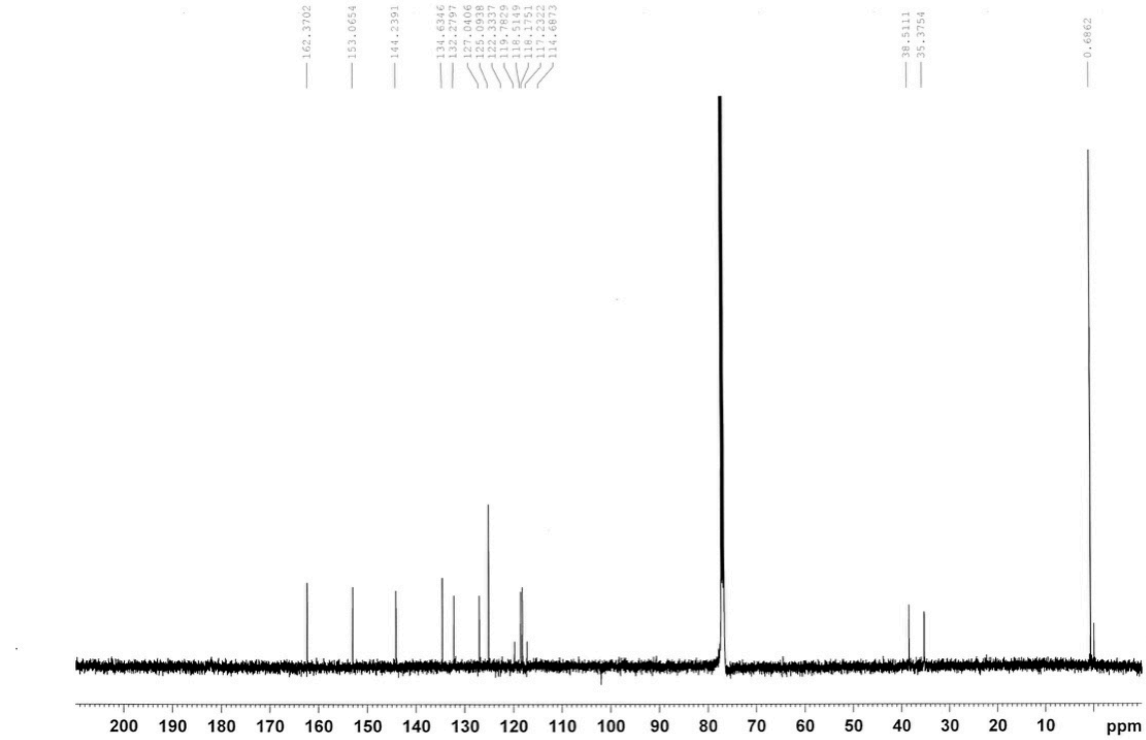

^{1}H NMR (500 MHz) and ^{13}C NMR (126 MHz) spectra of **6b’** ($CDCl_3$)

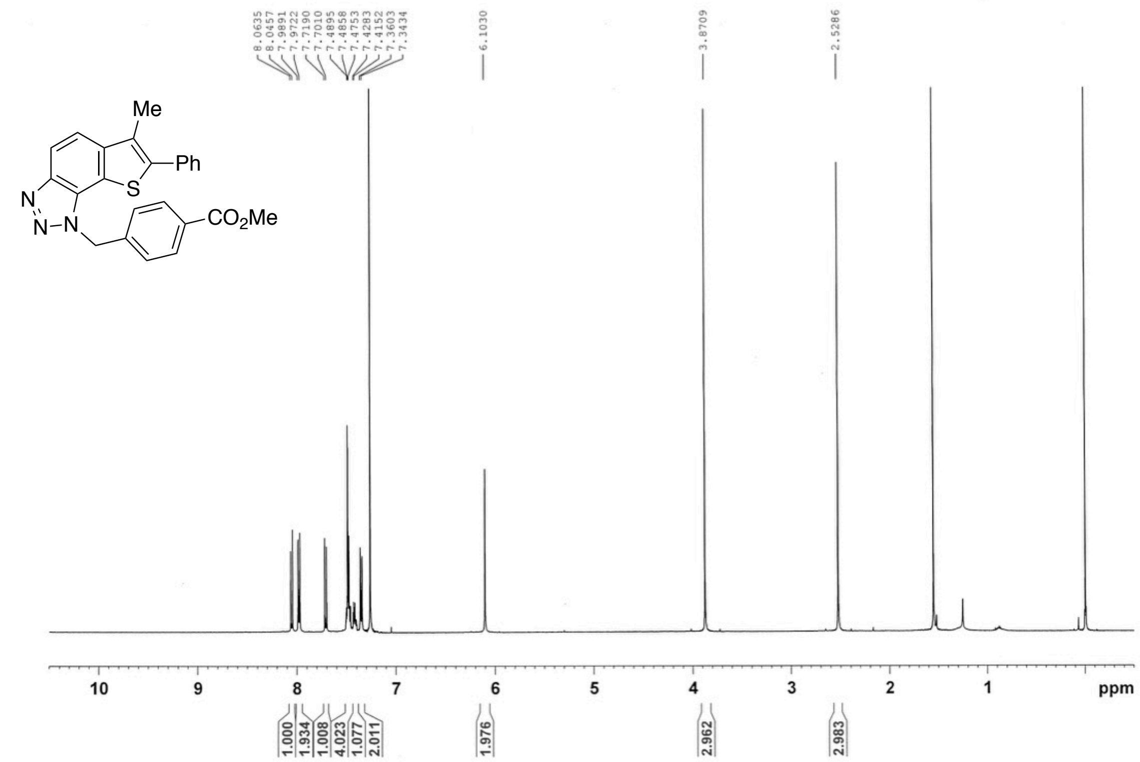

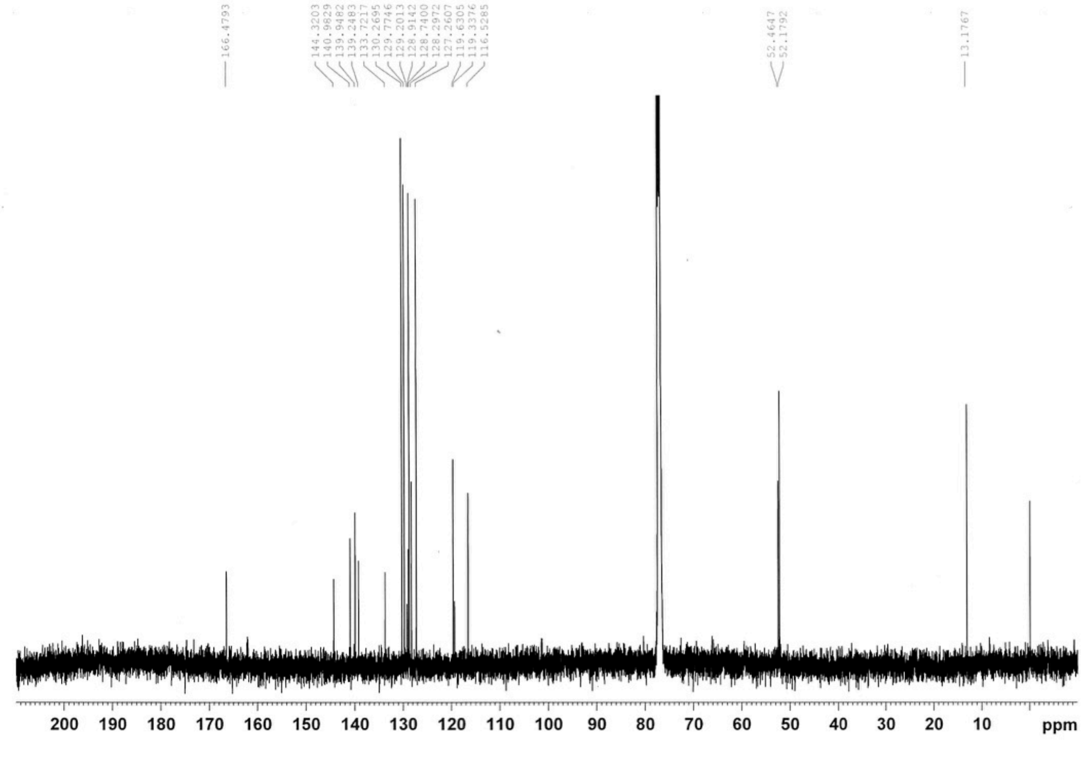

^{1}H NMR (500 MHz) and ^{13}C NMR (126 MHz) spectra of **13** ($CDCl_3$)

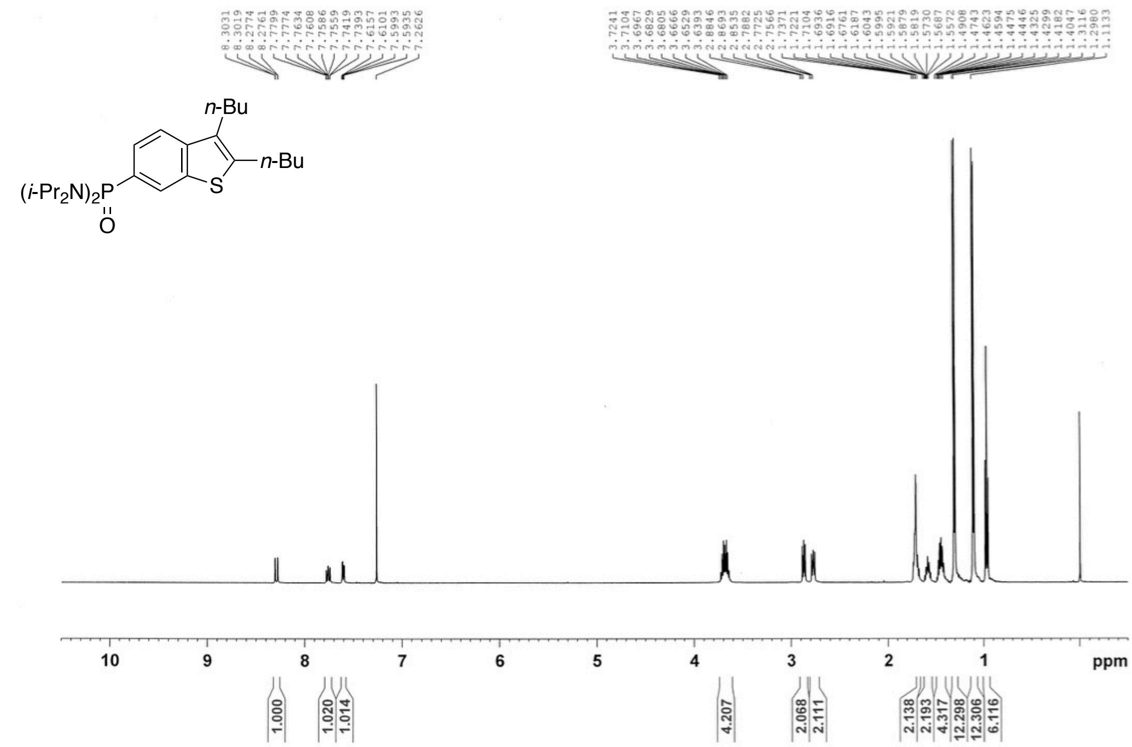

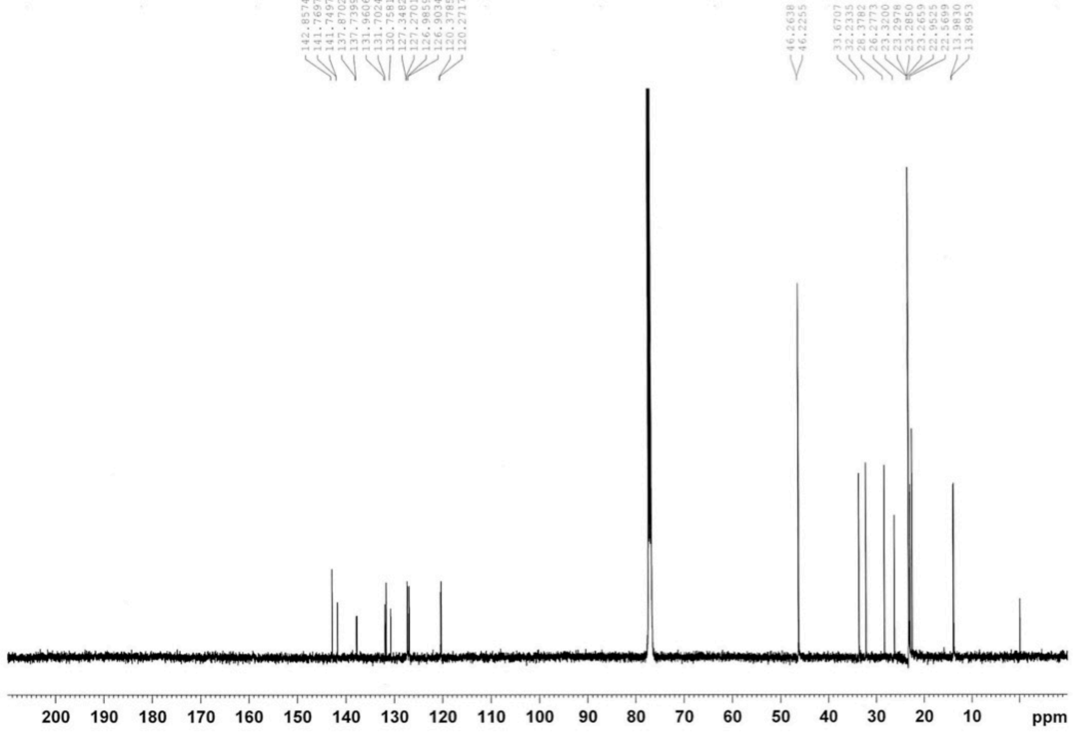

^{1}H NMR (500 MHz) and ^{13}C NMR (126 MHz) spectra of **15** ($CDCl_3$)

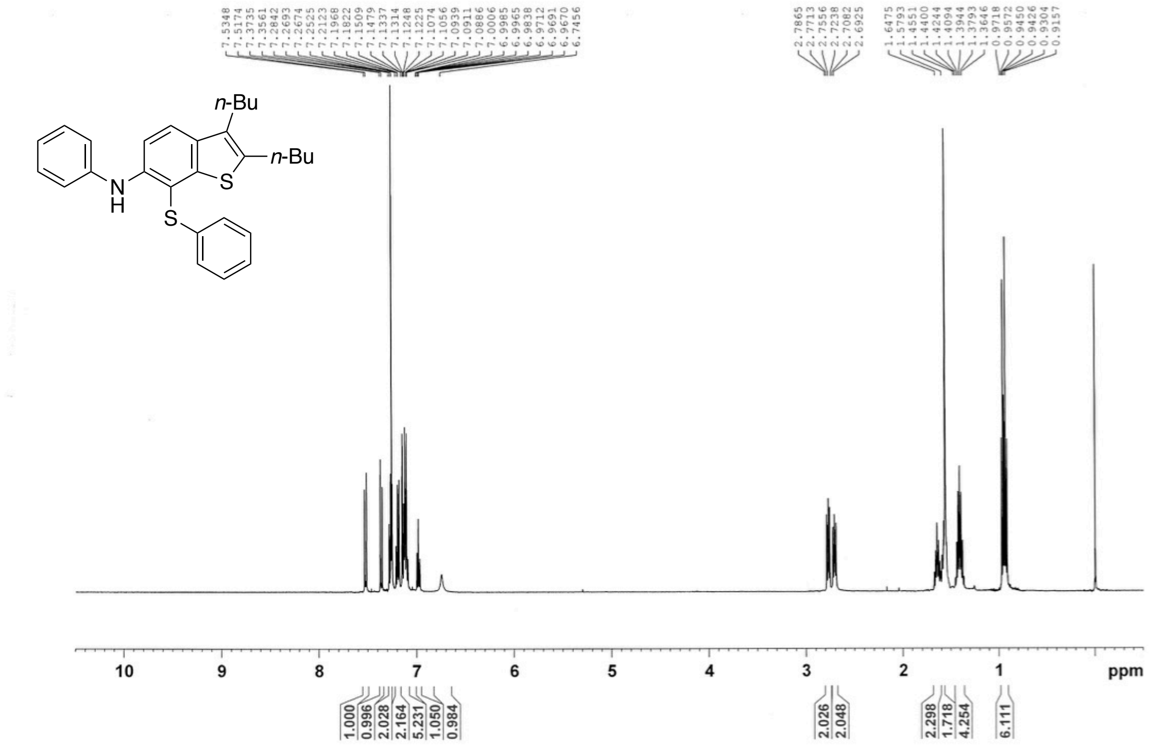

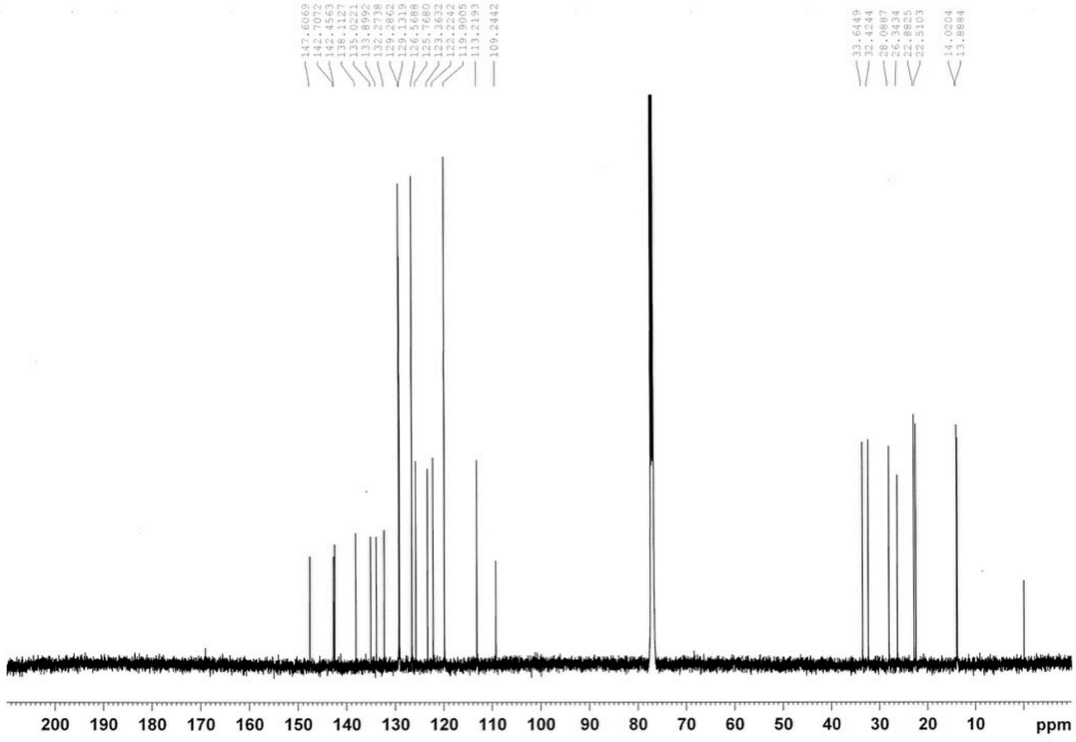

^{1}H NMR (500 MHz) and ^{13}C NMR (126 MHz) spectra of **15’** ($CDCl_3$)

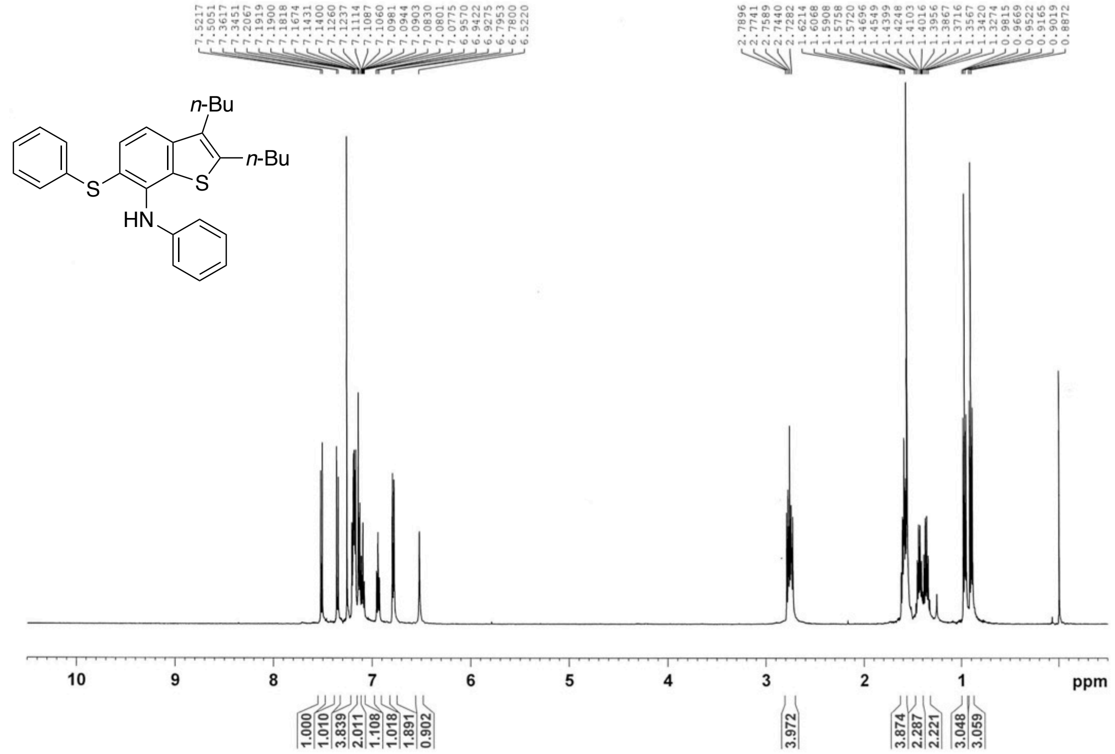

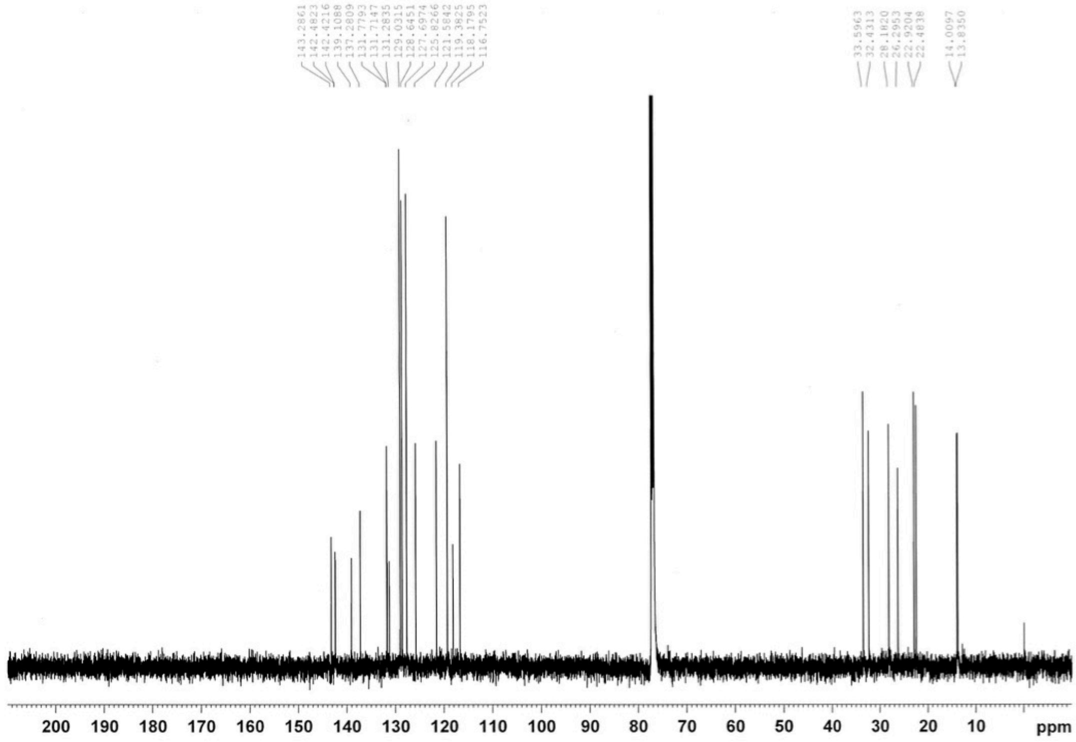

^{1}H NMR (500 MHz) and ^{13}C NMR (126 MHz) spectra of **17** ($CDCl_3$)

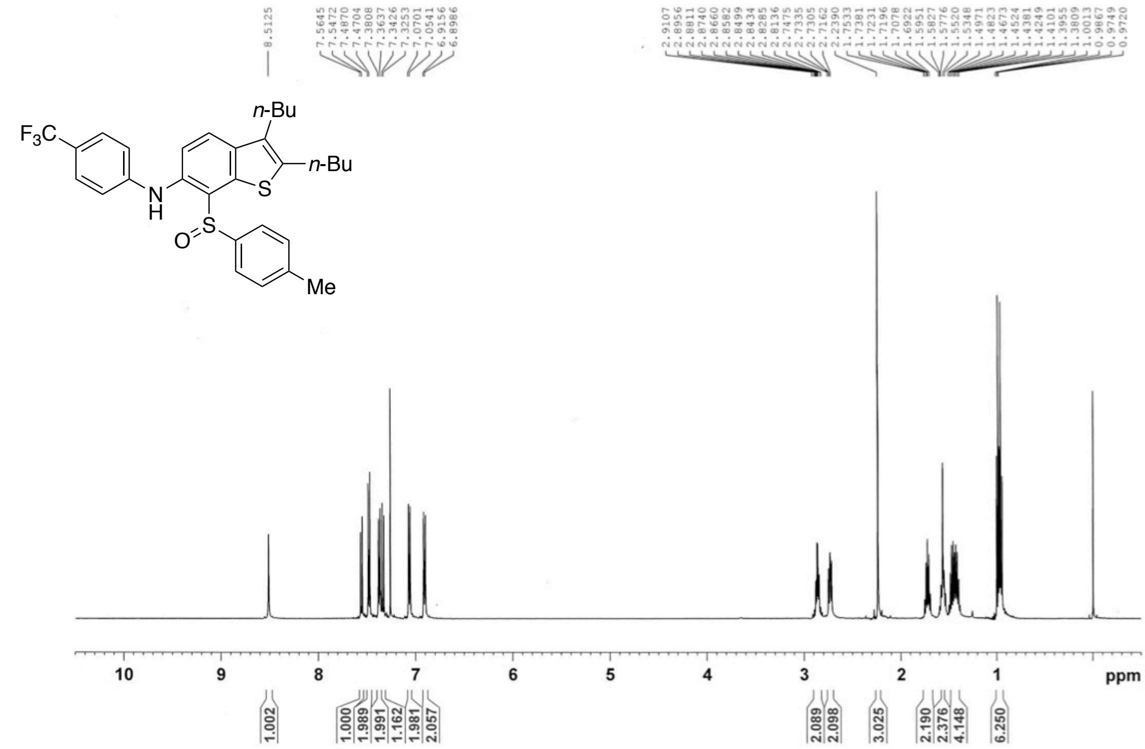

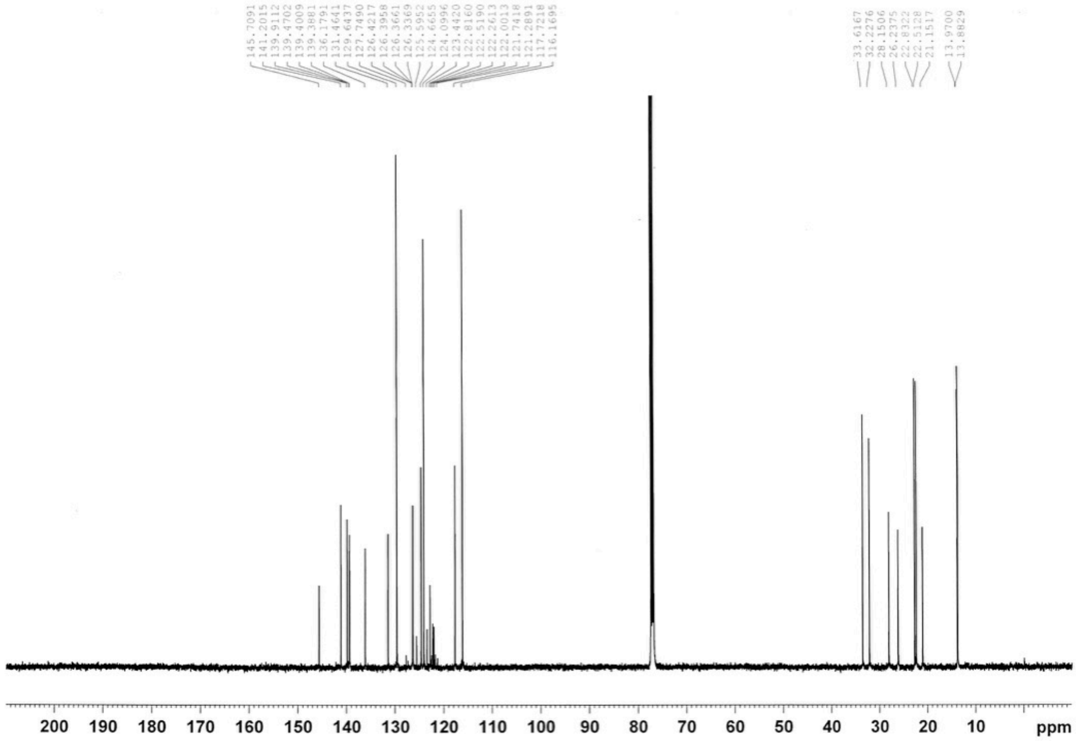

^{1}H NMR (500 MHz) and ^{13}C NMR (126 MHz) spectra of **19** ($CDCl_3$)

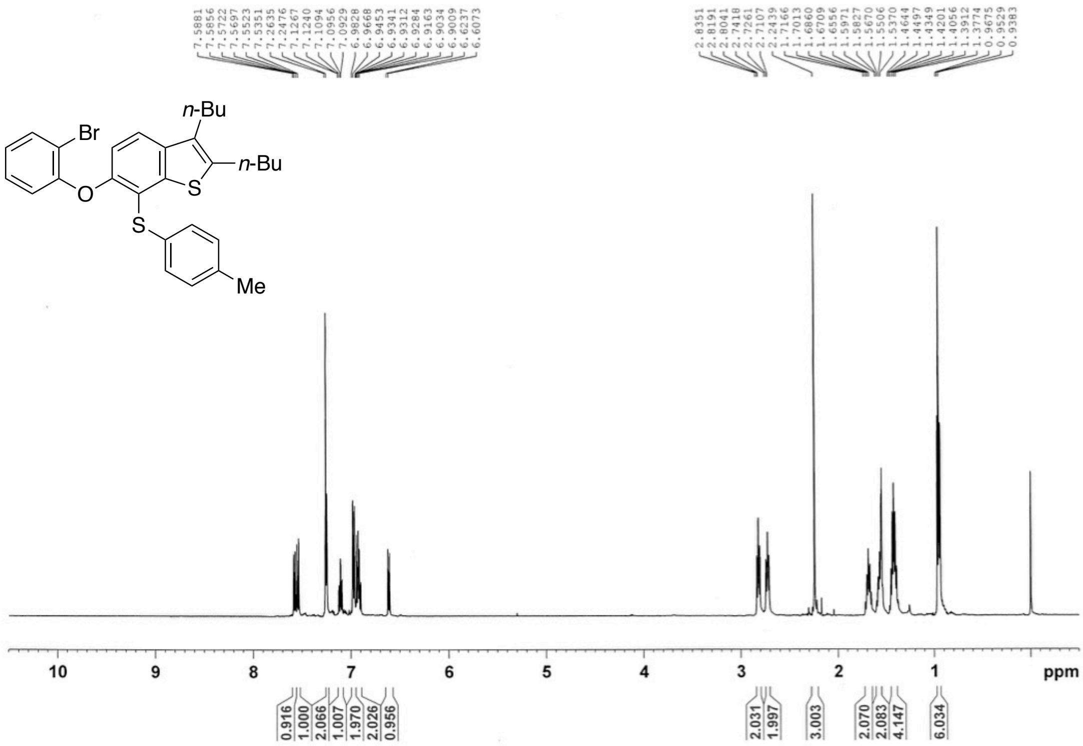

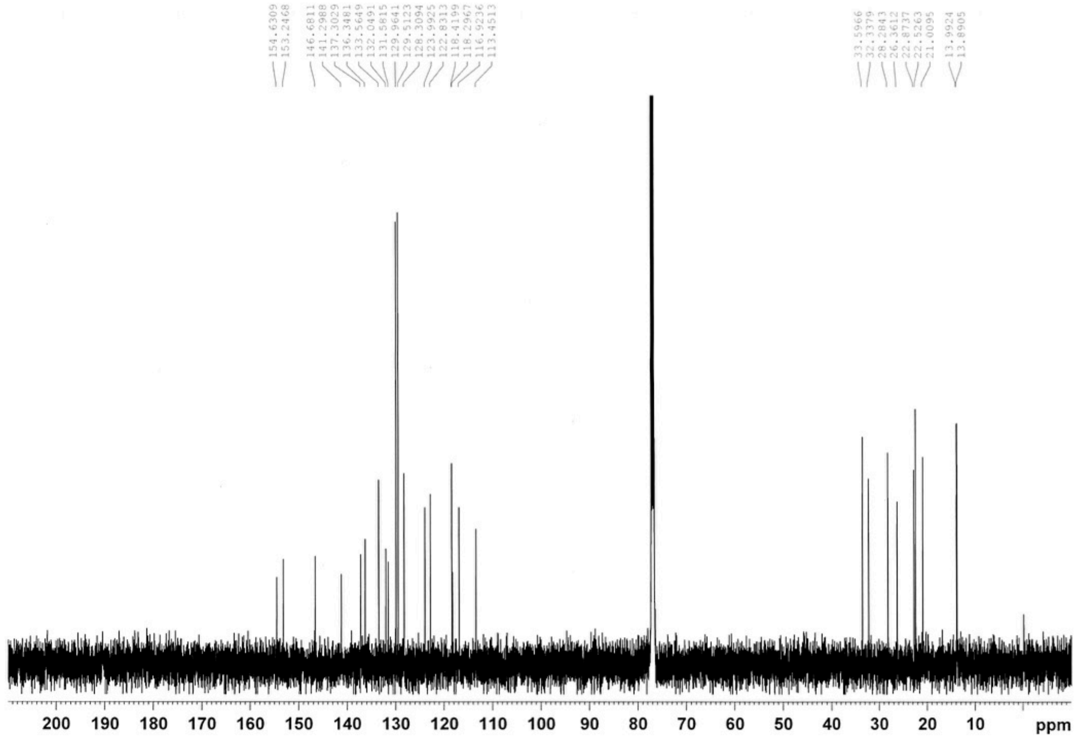

^{1}H NMR (500 MHz) and ^{13}C NMR (126 MHz) spectra of **21b** ($CDCl_3$)

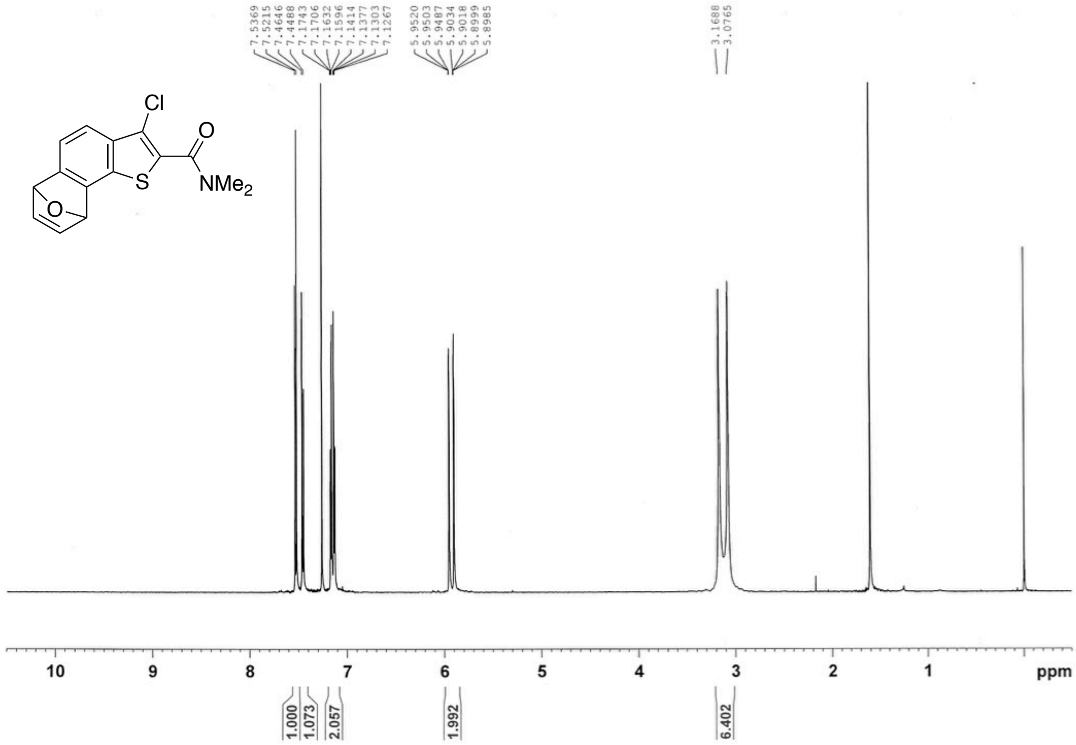

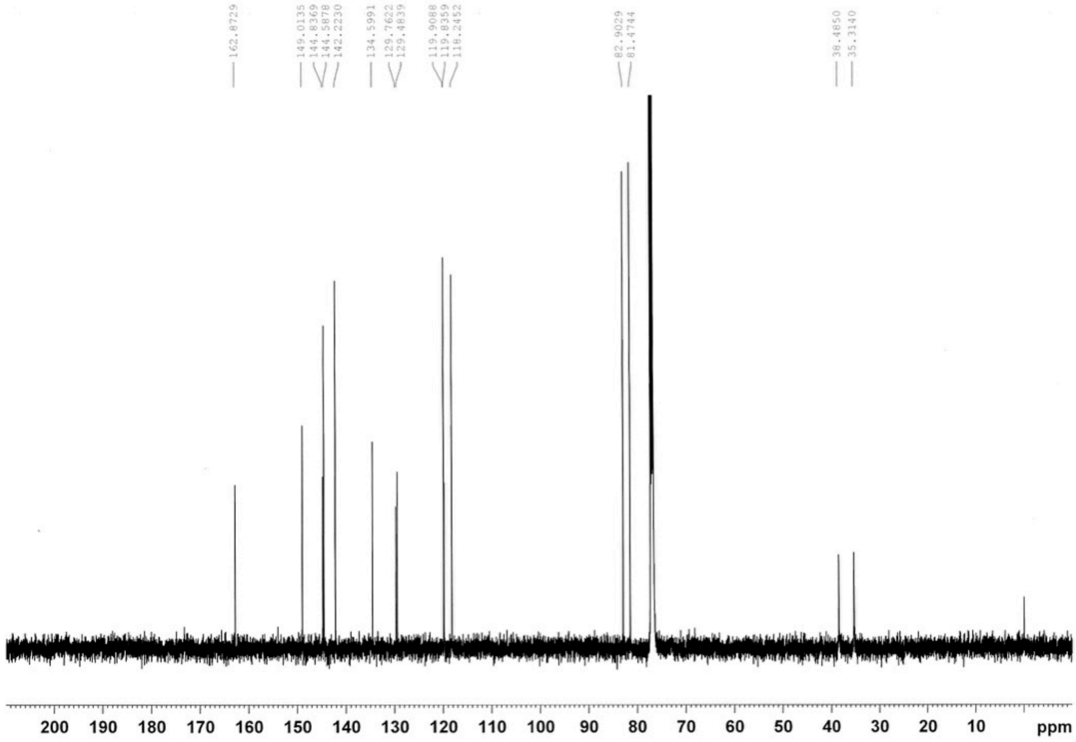

^{1}H NMR (500 MHz) and ^{13}C NMR (126 MHz) spectra of 3-chloro-2-(dimethylaminocarbonyl)naphtho[1,2-*b*]thiophene ($CDCl_3$)

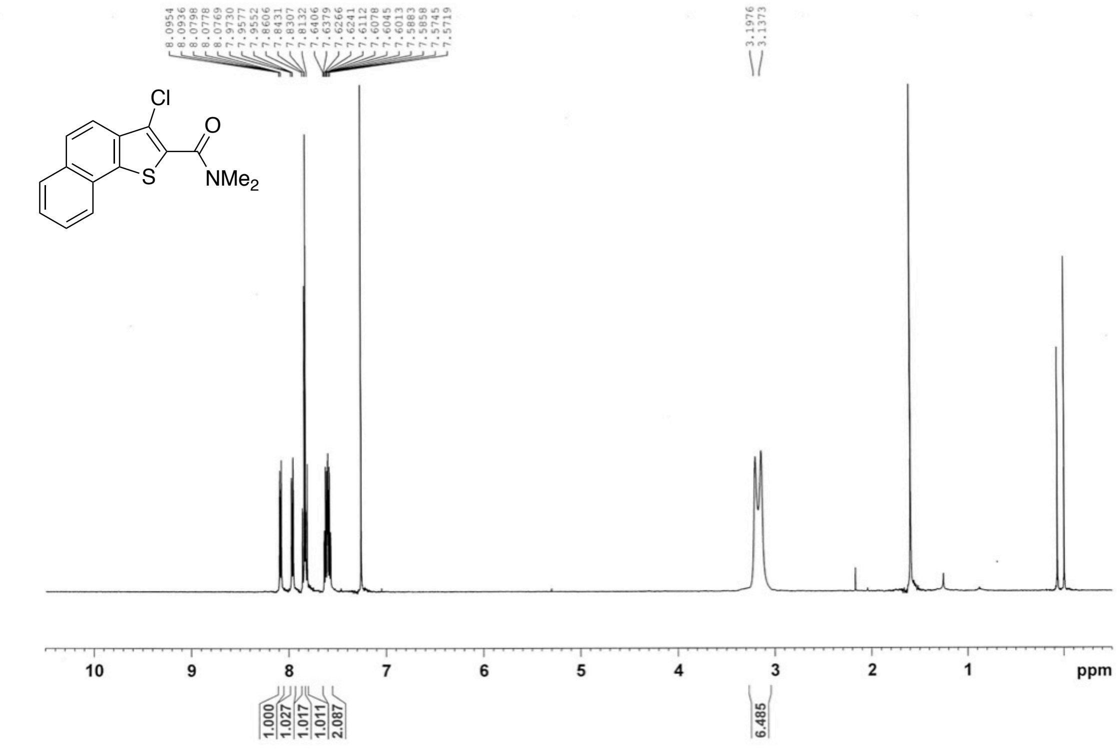

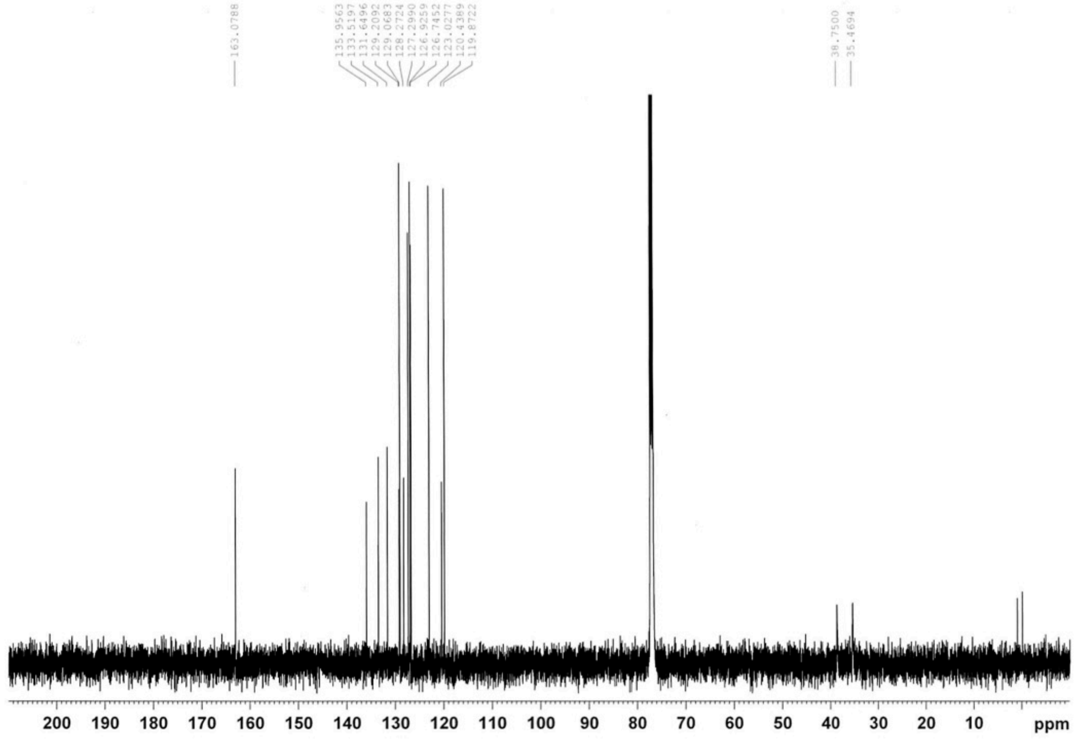

^{1}H NMR (500 MHz) and ^{13}C NMR (126 MHz) spectra of 2-(dimethylaminocarbonyl)-3-(4-(2-hydroxyethyl)-phenyl)naphtho[1,2-*b*]thiophene ($CDCl_3$)

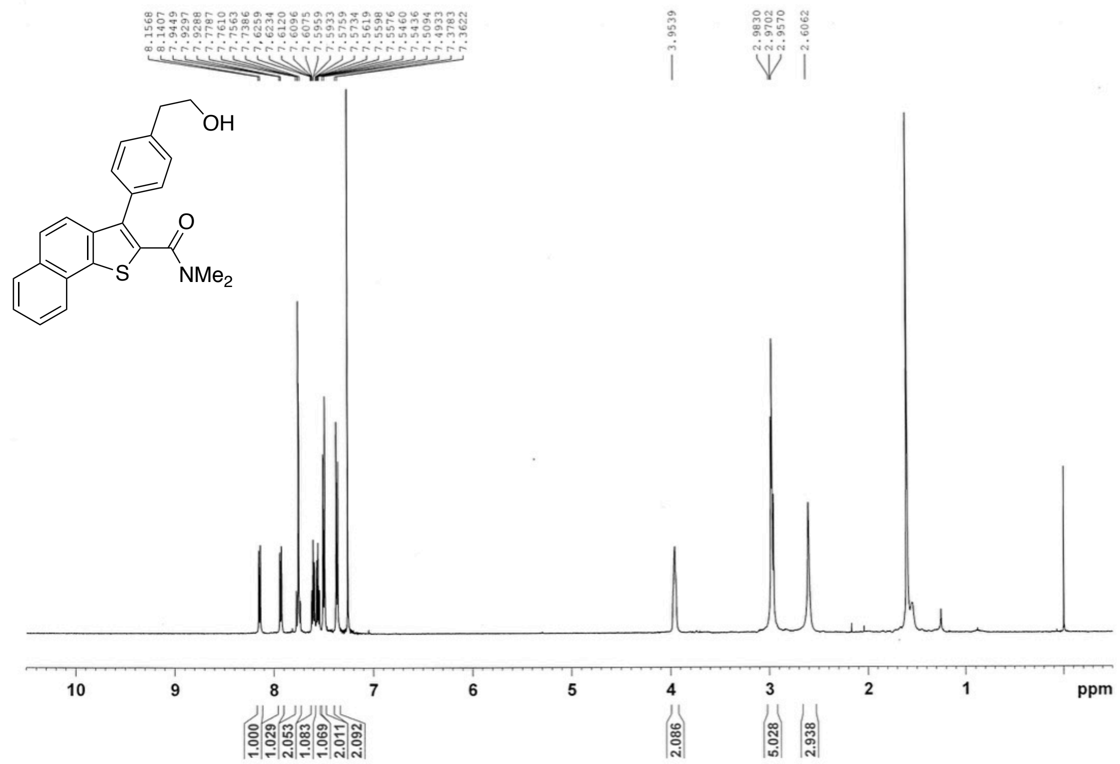

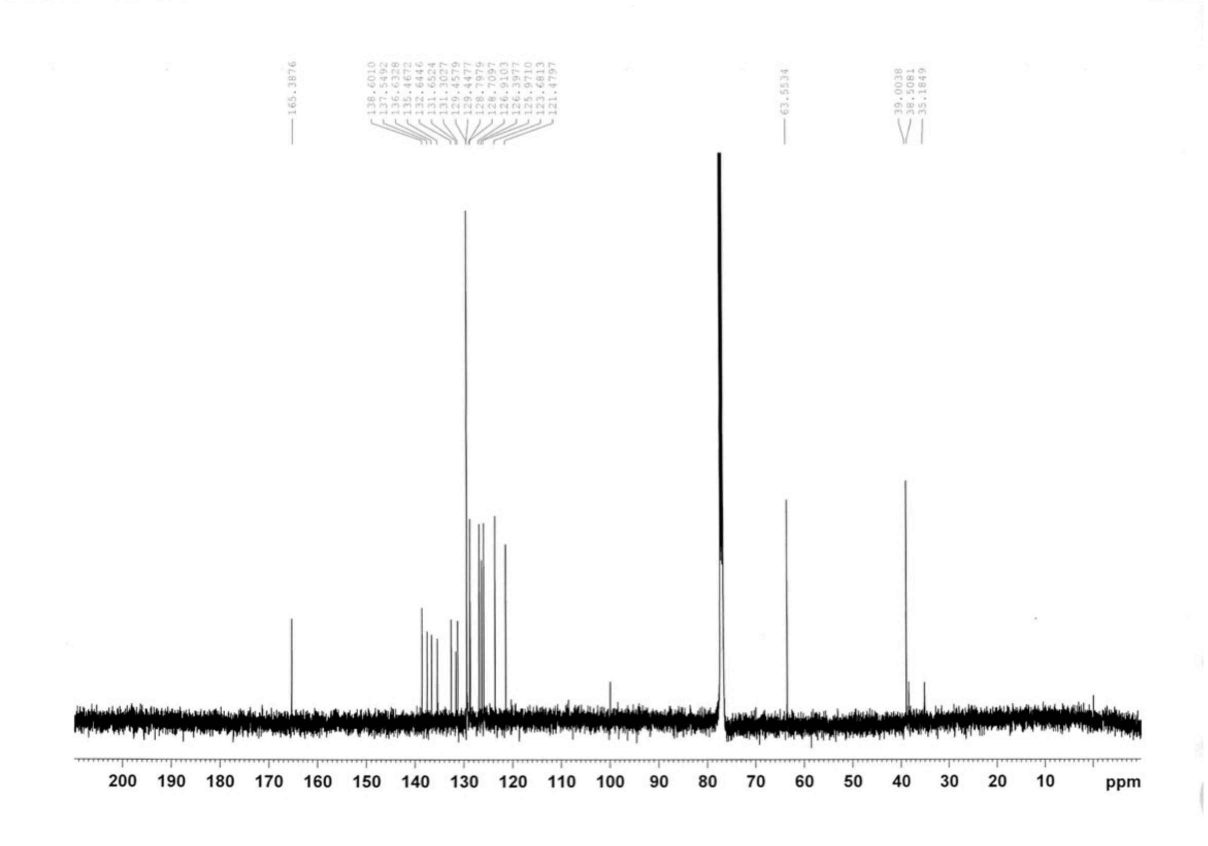

^{1}H NMR (500 MHz) and ^{13}C NMR (126 MHz) spectra of 2-dimethylaminocarbonyl-3-(4-(2-(1,3-dioxoiso-indolin-2-yl)ethyl)phenyl)naphtho[1,2-*b*]thiophene ($CDCl_3$)

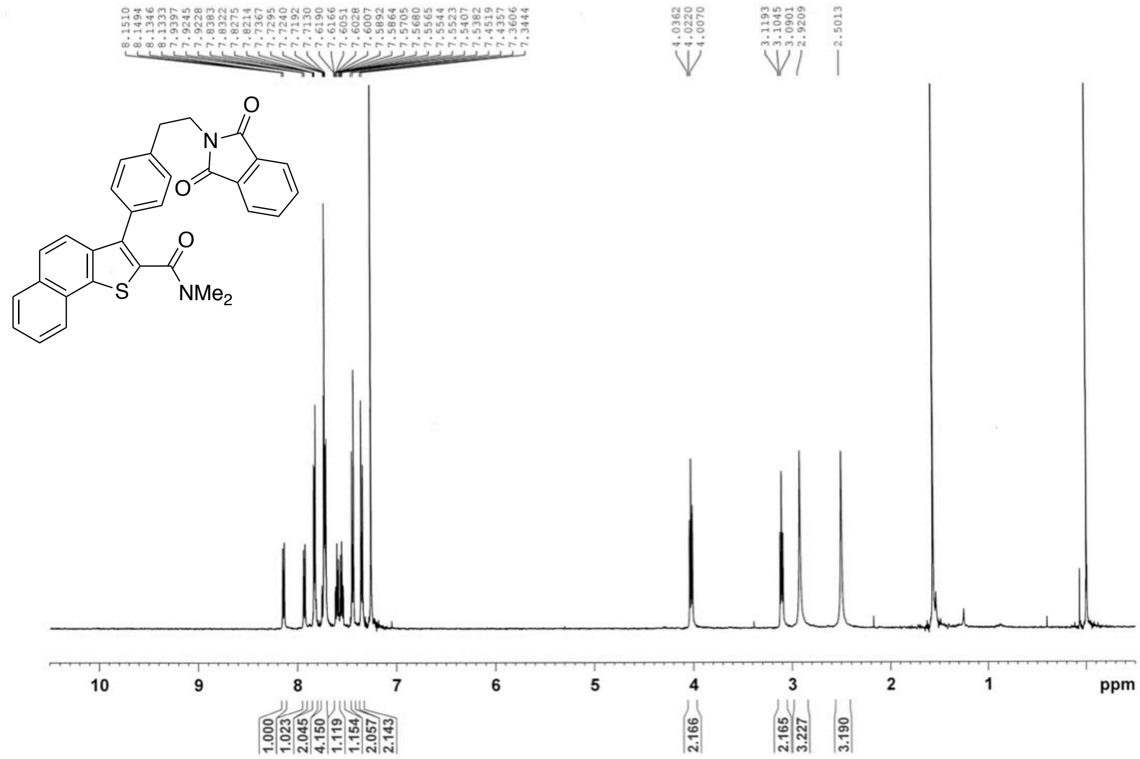

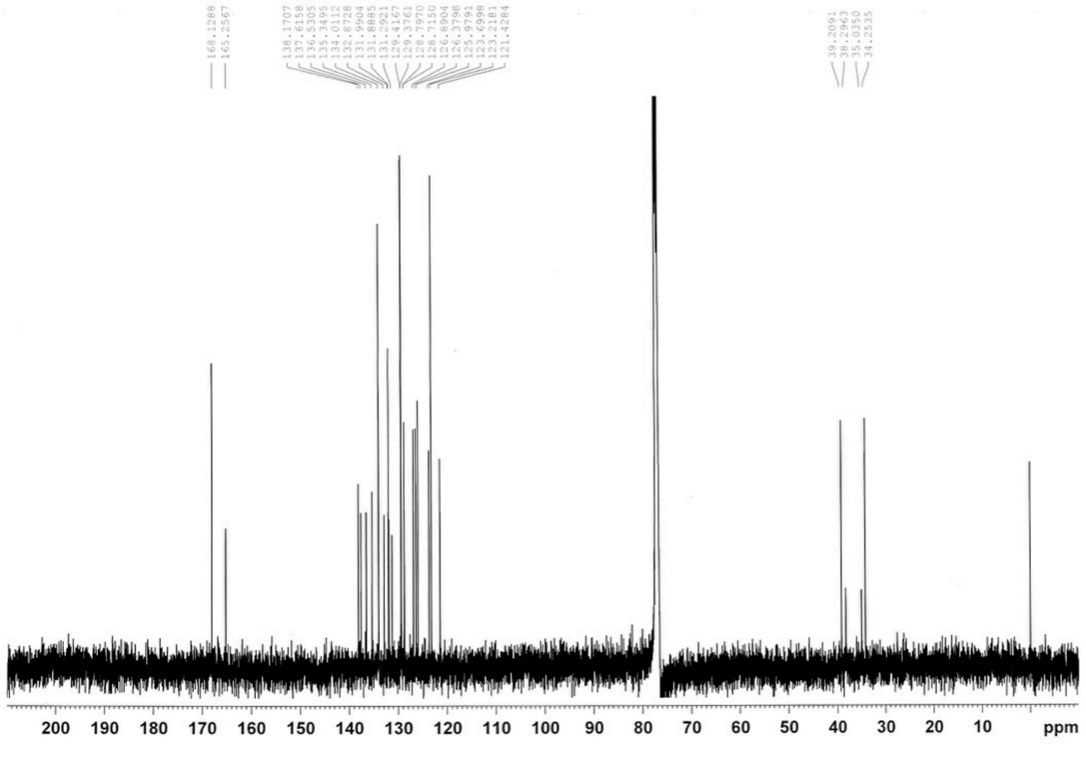

^{1}H NMR (500 MHz) and ^{13}C NMR (126 MHz) spectra of **20c** ($CDCl_3$)

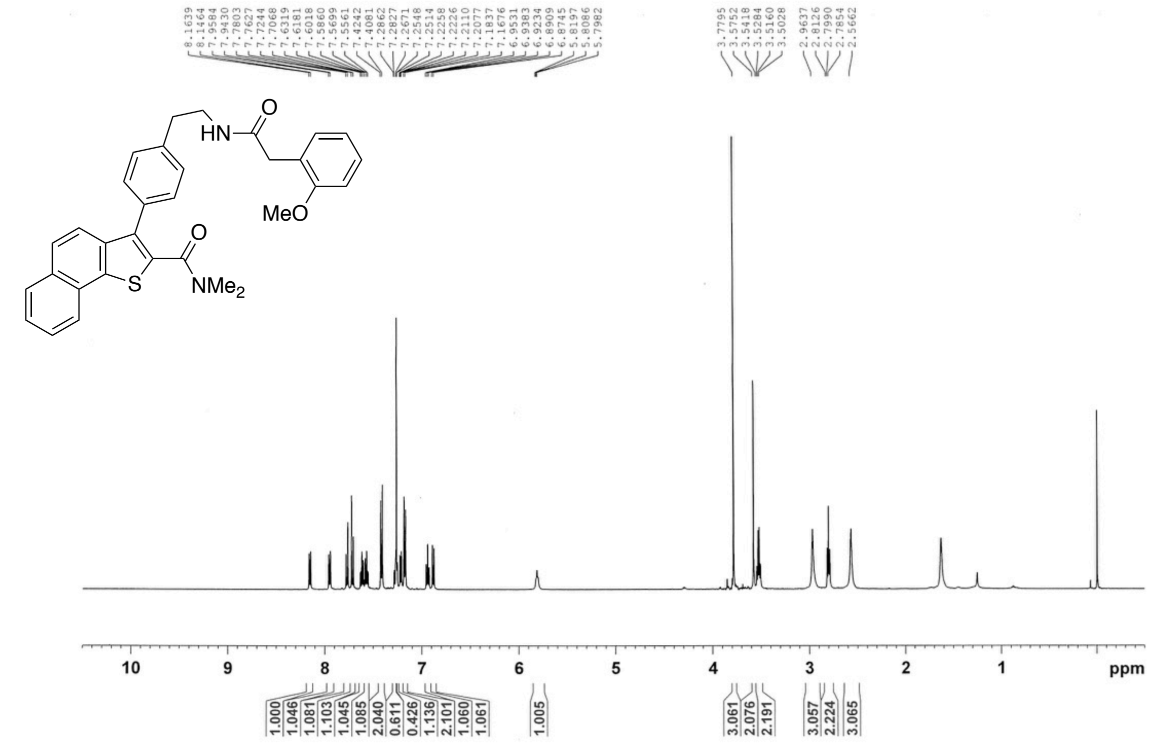

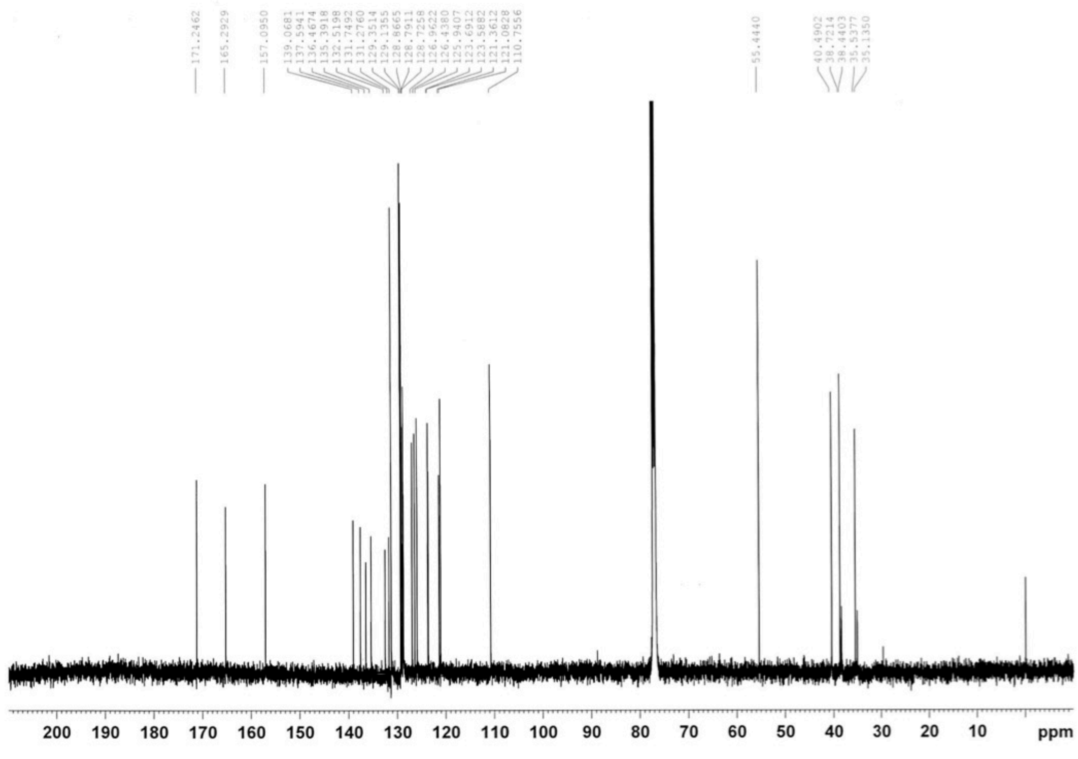

^{1}H NMR (500 MHz) and ^{13}C NMR (126 MHz) spectra of **21c** ($CDCl_3$)

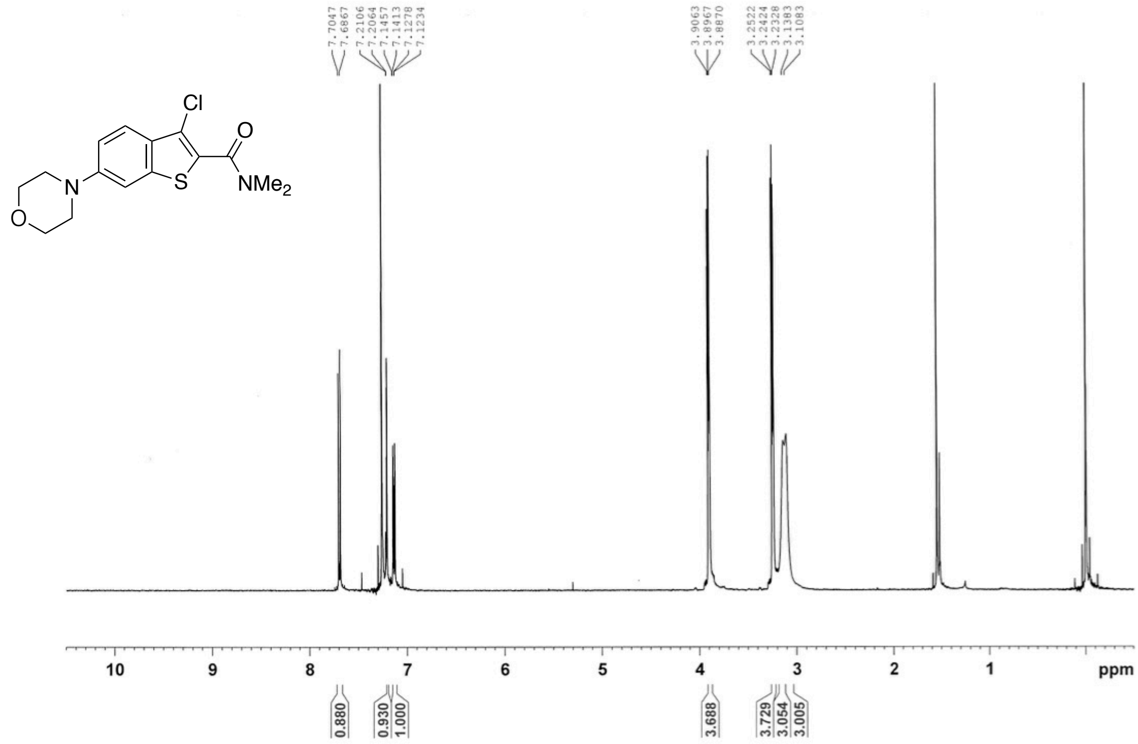

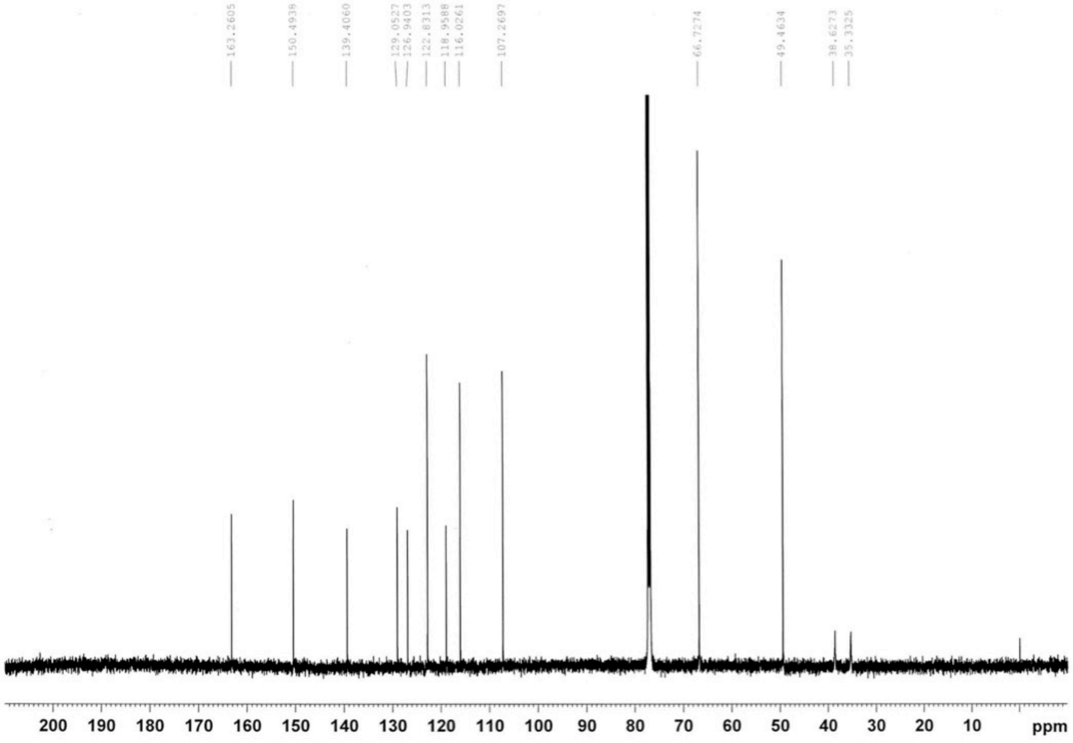

^{1}H NMR (500 MHz) and ^{13}C NMR (126 MHz) spectra of **21c'** ($CDCl_3$)

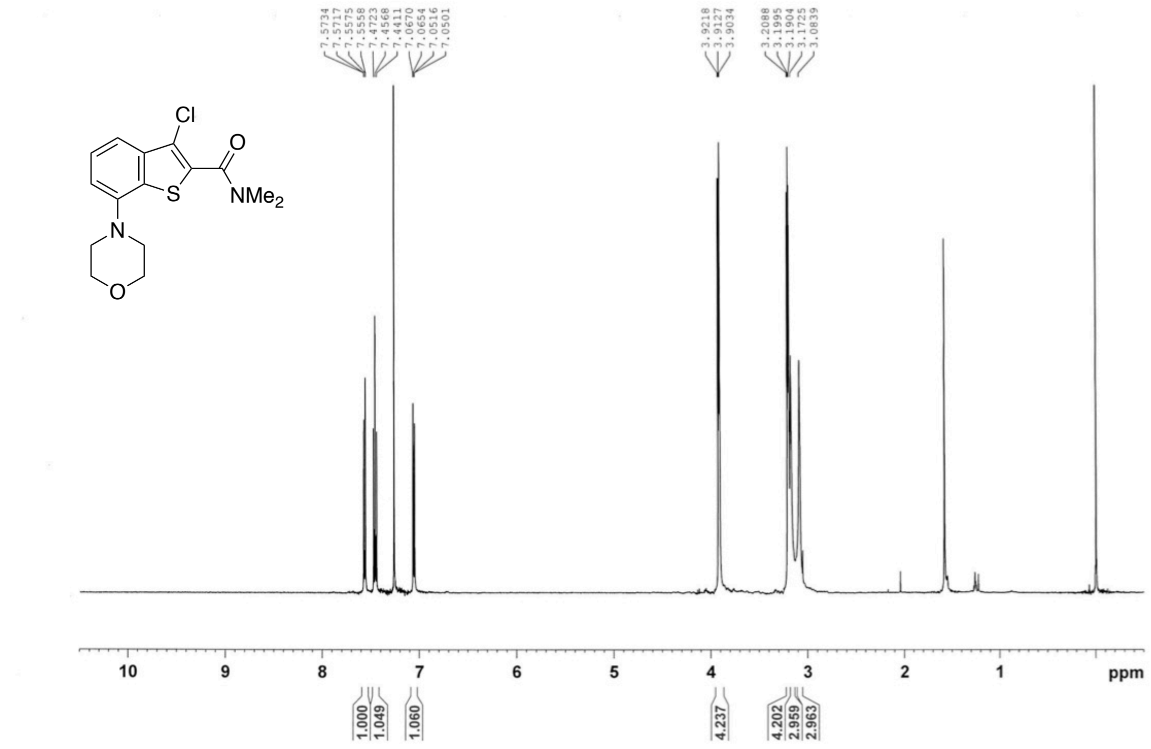

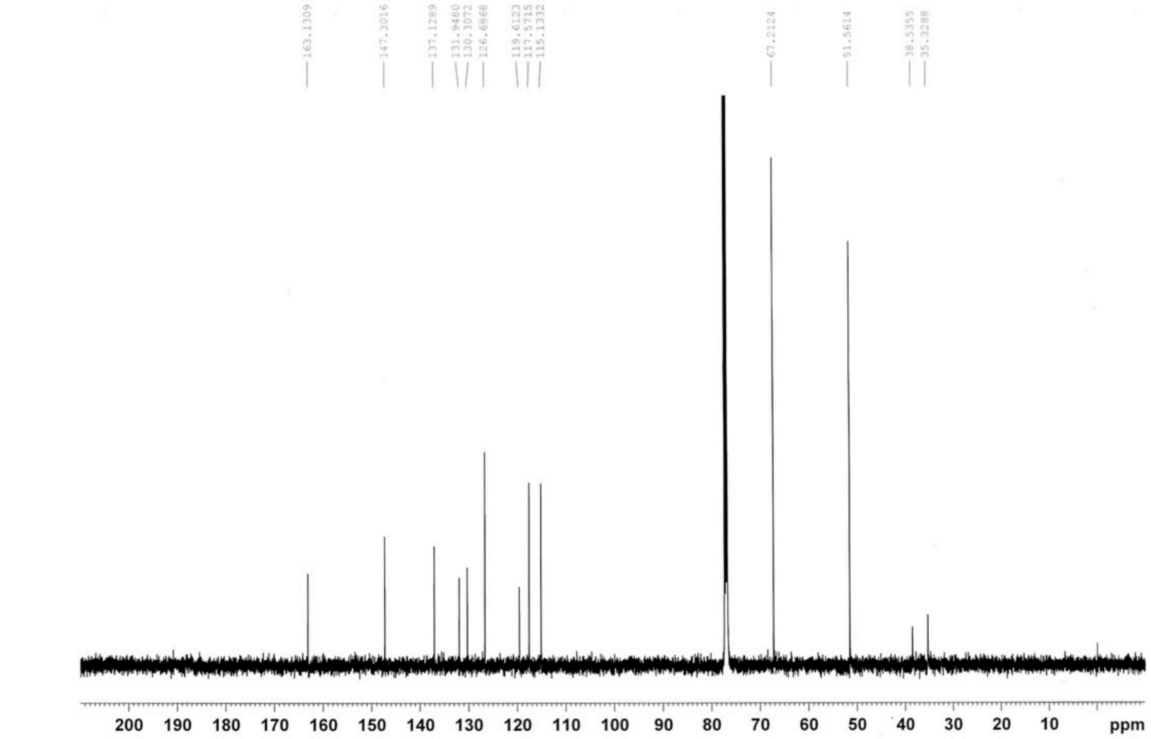

^{1}H NMR (500 MHz) and ^{13}C NMR (126 MHz) spectra of 2-dimethylaminocarbonyl-3-(4-(2-hydroxyethyl)-phenyl)-6-morpholinobenzo[*b*]thiophene ($CDCl_3$)

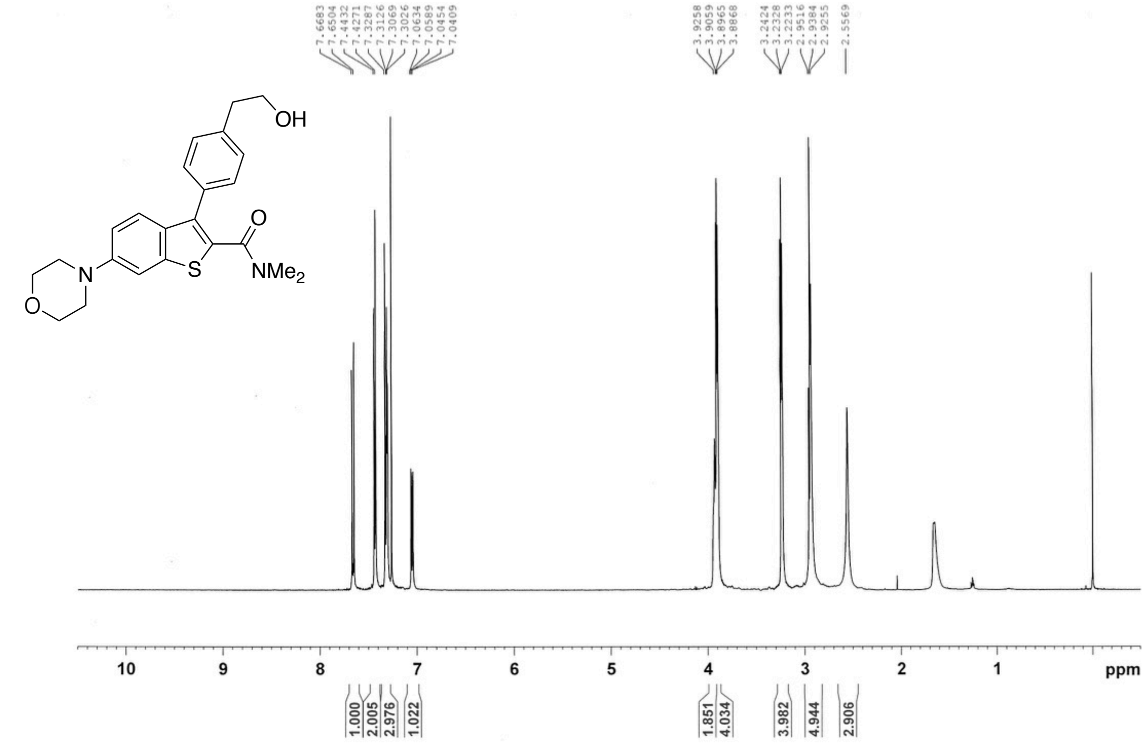

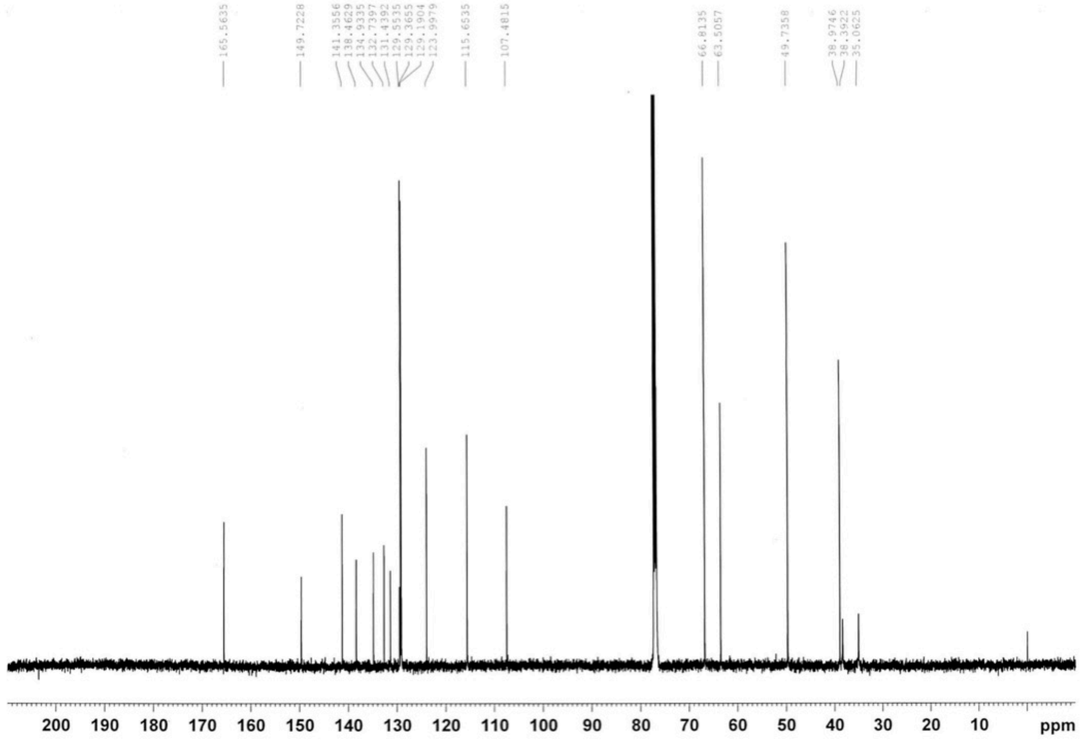

^{1}H NMR (500 MHz) and ^{13}C NMR (126 MHz) spectra of 2-dimethylaminocarbonyl-3-(4-(2-(1,3-dioxoiso-indolin-2-yl)ethyl)phenyl)-6-morpholinobenzo[*b*]thiophene ($CDCl_3$)

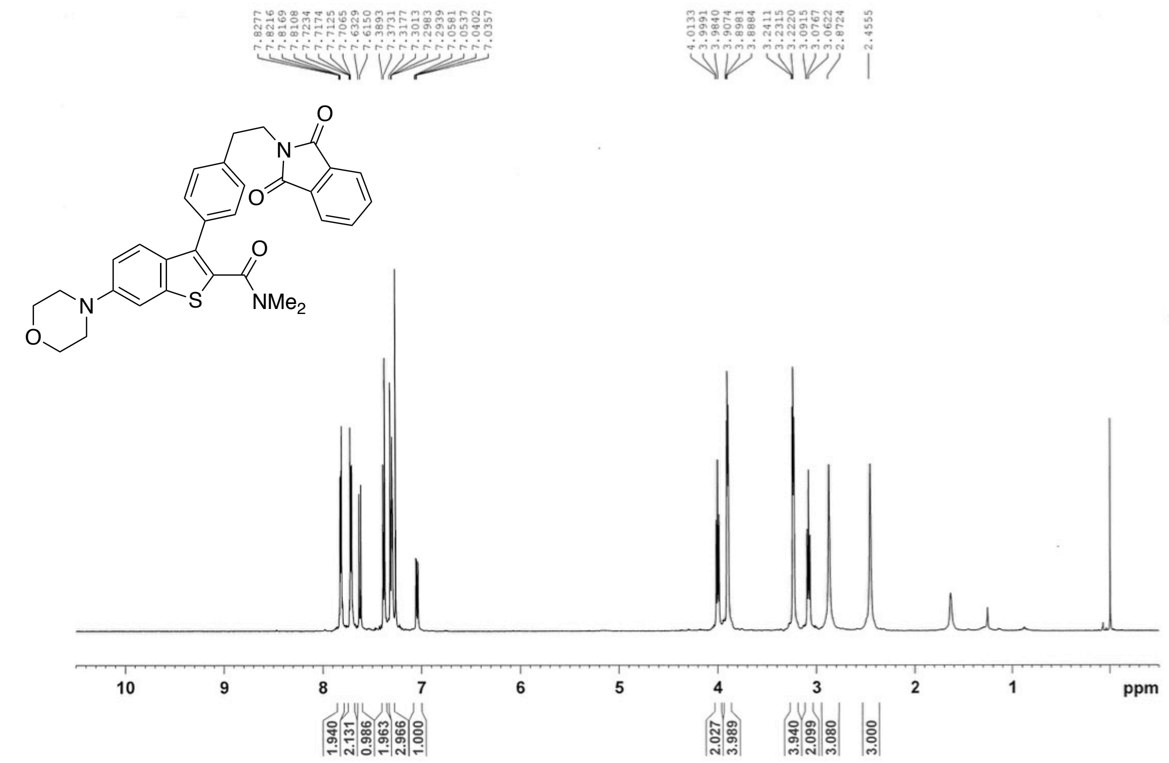

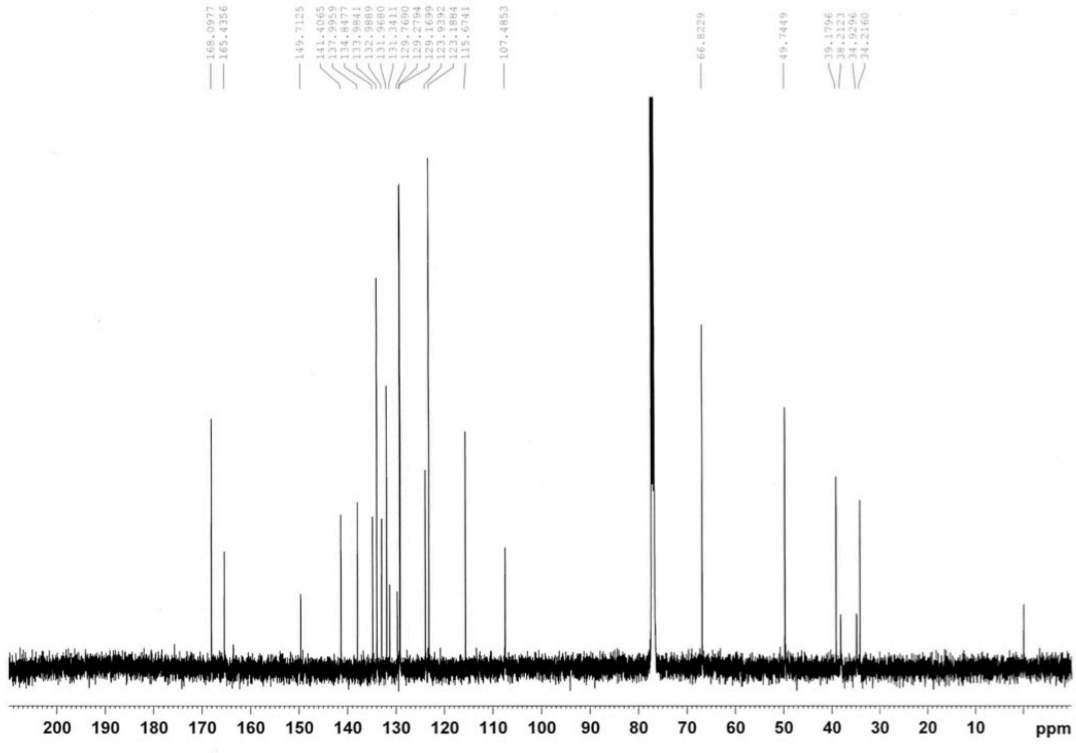

^{1}H NMR (500 MHz) and ^{13}C NMR (126 MHz) spectra of **20d** ($CDCl_3$)

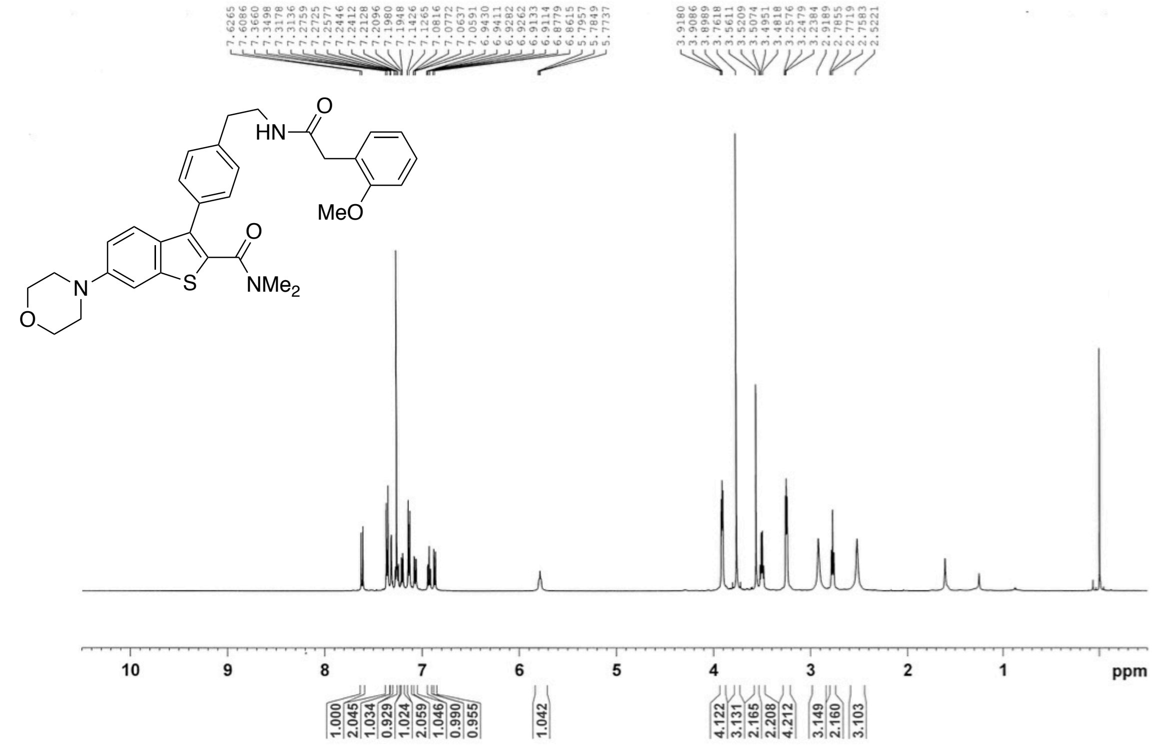

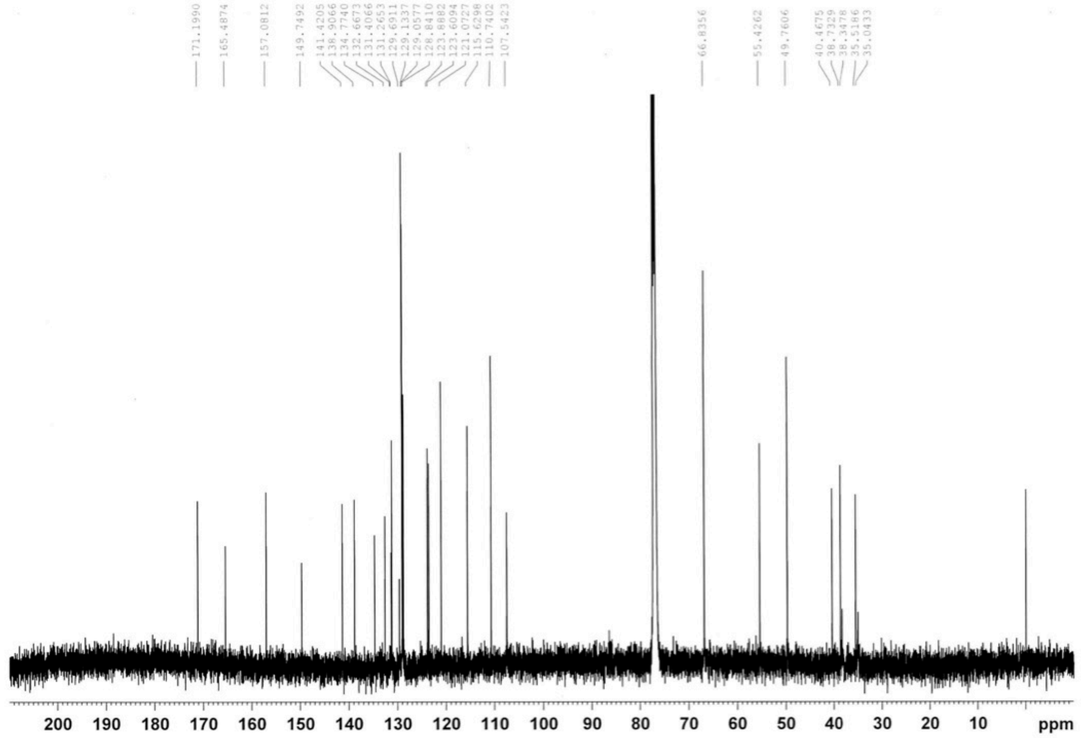

Supplement: RA-008-C8RA04035D-s001 [file RA-008-C8RA04035D-s001.pdf]
